# Co/NHPI-mediated aerobic oxygenation of benzylic C–H bonds in pharmaceutically relevant molecules†
†Electronic supplementary information (ESI) available: Screening data, experimental protocols, characterization data. See DOI: 10.1039/c6sc03831j



**DOI:** 10.1039/c6sc03831j

**Published:** 2016-10-07

**Authors:** Damian P. Hruszkewycz, Kelsey C. Miles, Oliver R. Thiel, Shannon S. Stahl

**Affiliations:** a Department of Chemistry , University of Wisconsin-Madison , 1101 University Avenue , Madison , Wisconsin 53706 , USA . Email: stahl@chem.wisc.edu; b Process Development, Drug Substance Technologies , Amgen Inc. , One Amgen Center Drive, Thousand Oaks , California 91320 , USA

## Abstract

A simple cobalt(ii)/*N*-hydroxyphthalimide catalyst system has been identified for selective conversion of benzylic methylene groups in pharmaceutically relevant (hetero)arenes to the corresponding (hetero)aryl ketones. The radical reaction pathway tolerates electronically diverse benzylic C–H bonds, contrasting recent oxygenation reactions that are initiated by deprotonation of a benzylic C–H bond. The reactions proceed under practical reaction conditions (1 M substrate in BuOAc or EtOAc solvent, 12 h, 90–100 °C), and they tolerate common heterocycles, such as pyridines and imidazoles. A cobalt-free, electrochemical, NHPI-catalyzed oxygenation method overcomes challenges encountered with chelating substrates that inhibit the chemical reaction. The utility of the aerobic oxidation method is showcased in the multigram synthesis of a key intermediate towards a drug candidate (**AMG 579**) under process-relevant reaction conditions.

## Abstract 2

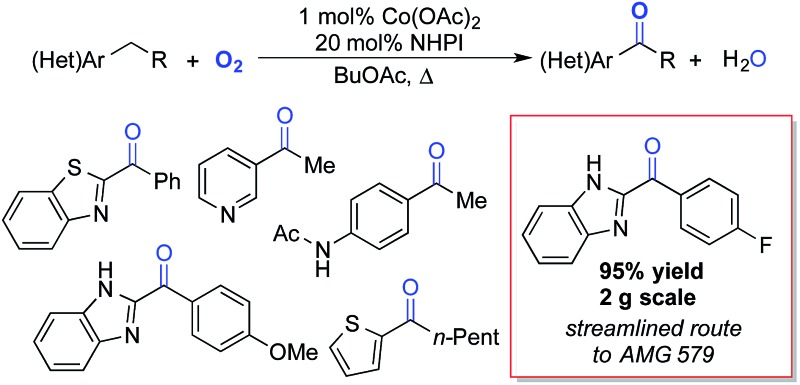
A new method for Co/NHPI-catalyzed aerobic C–H oxygenation shows excellent tolerance of electronically diverse heterocycles.

## Introduction

**Scheme 1:**
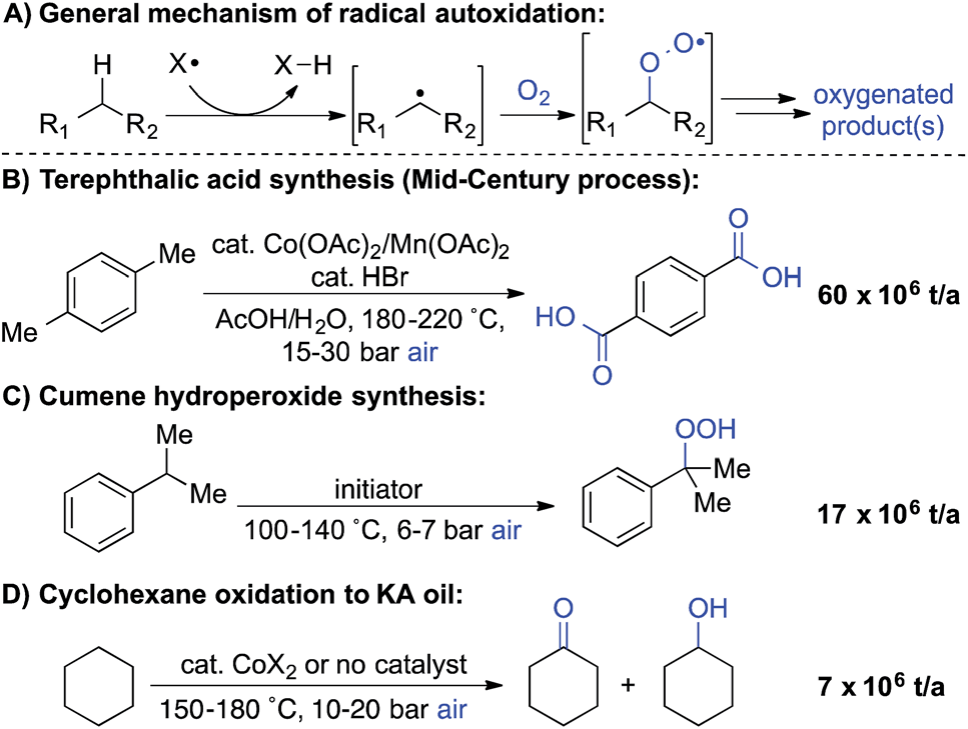
Summary of major industrial radical autoxidation processes.

Liquid-phase radical-chain autoxidation reactions are amongst the largest-scale oxidation reactions performed in industry (Scheme 1).^1^ Prominent examples include the Co/Mn/Br-catalyzed oxidation of *p*-xylene to terephthalic acid in variations of the Mid-Century process (Scheme 1B),^2^ autoxidation of cumene *en route* to phenol and acetone in the Hock process (Scheme 1C)^3^ and radical-chain autoxidation of cyclohexane to a mixture of cyclohexanone and cyclohexanol (“KA oil”, Scheme 1D).^4^ In contrast to these prominent large-scale applications, aerobic oxidations and radical autoxidation reactions, in particular, are rarely used for the production of pharmaceuticals or related complex molecules.

**Scheme 2:**
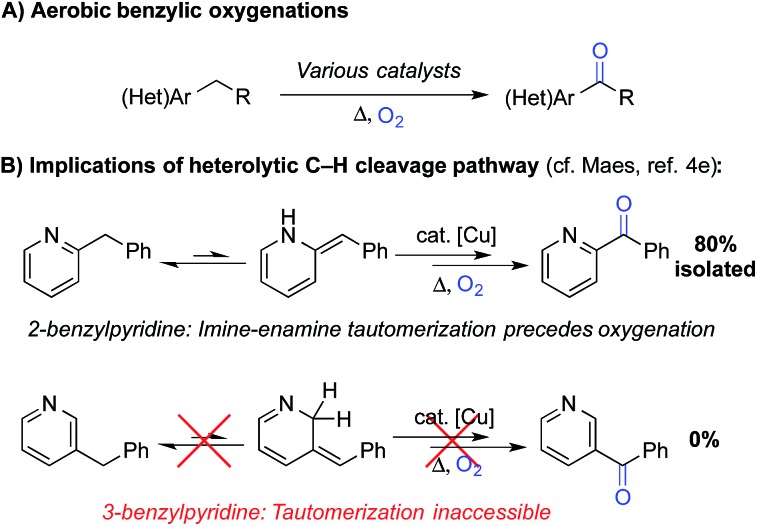
Recent work on aerobic benzylic oxygenation.

A number of groups have recently reported methods for aerobic oxygenation of benzylic C–H bonds^5,6^ (Scheme 2A). The reactions are often compatible with heterocycles and other heteroatom-containing functional groups, suggesting they could be well suited for use in pharmaceutical applications. Further studies have shown that heterocycles can play an important role in promoting the reaction by facilitating formation of an enamine or related tautomer that is more susceptible to aerobic oxygenation (Scheme 2B).^7^ In a representative example, Maes and coworkers developed a copper-catalysed oxygenation method, in which they contrasted successful oxygenation of 2- and 4-benzylpyridine with the poor reactivity of 3-benzylpyridine.^6*e*^ A subsequent mechanistic study provided evidence for a heterolytic C–H cleavage pathway and the involvement of the benzylpyridine enamine tautomer (Scheme 2B).^6*l*^

**Scheme 3:**
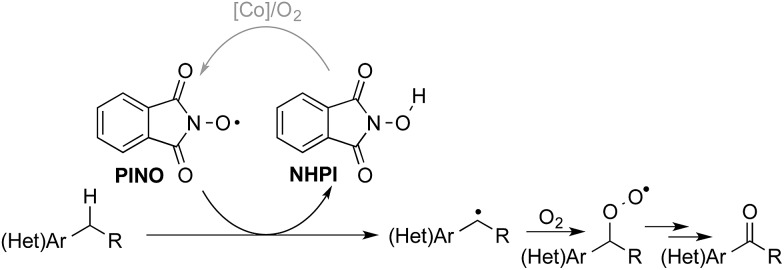
Simplified mechanism depicting C–H abstraction by phthalimido-*N*-oxyl (PINO) and radical oxygenation by O_2_.

The “heterolytic” C–H oxygenation reactions show broad synthetic appeal, but the mechanism limits their utility to substrates with relatively acidic benzylic C–H bonds. In this context, we sought an alternative method that could bypass this limitation, and our attention was drawn to the *N*-hydroxyphthalimide (NHPI)-based C–H oxygenation reactions.^8^ In the 1990s, Ishii and coworkers showed that use of redox-active metal salts, such as Co(OAc)_2_, in combination with O_2_ enable efficient conversion of NHPI into the phthalimido-*N*-oxyl (PINO) radical.^9^ PINO then mediates selective abstraction of weak C–H bonds (Scheme 3) to generate an organic radical that can react with O_2_ to afford the oxygenated products.^10^ This methodology has been demonstrated in numerous oxidation reactions, including industrial applications,^8^ but the vast majority of studies have focused on simple hydrocarbon substrates.^11^

Here, we explore Co/NHPI-catalyzed benzylic^12^ oxygenation reactions of substrates bearing common heterocycles, such as pyridines, benzimidazoles and thiophenes, and benzylic ketones are often obtained in good-to-excellent yield. Notably, the radical autoxidation pathway enables oxygenation of non-acidic substrates that are ineffective with catalyst systems that mediate oxygenation *via* a heterolytic pathway. For reactions that appear to be inhibited by heterocycle chelation to the Co co-catalyst, we show that a Co-free, electrochemical NHPI-mediated method offers a promising alternative approach. Finally, we demonstrate the practicality and scalability of the Co/NHPI catalytic conditions in the multigram synthesis of a pharmaceutical intermediate.

## Results and discussion

We began our studies by evaluating the oxygenation of 3-ethylpyridine (**1a**) to 3-acetylpyridine (**2a**) (Table 1). This reaction is expected to be problematic for many of the recently reported oxygenation methods. First, we used Co(OAc)_2_ as the cobalt source and compared solvents that have been considered previously in Ishii-type oxidation reactions (entries 1–4).^13^ A superior yield was obtained using EtOAc (entry 1), and a similar yield was obtained with the higher boiling ester solvent, *n*-BuOAc (entry 5). The identity of the counterion in the CoX_2_ co-catalyst had a marked effect on catalytic efficiency (entries 5–10). The best yields were obtained with Co(OAc)_2_·4H_2_O and other Co^II^-carboxylate salts (see Tables S1 and S2 in the ESI† for full screening data). Use of BuOAc solvent enabled access to higher reaction temperatures (entries 9 and 10), and an excellent yield of **2a** was achieved at 90 °C (94%, entry 10).

**Table 1:** Optimization of reaction conditions^*a*^

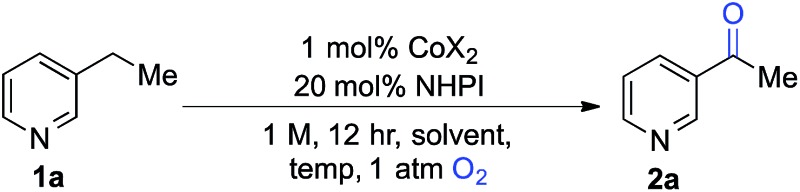					
Entry	CoX_2_	Solvent	Temp (°C)	Conv.^*b*^ (%)	Yield^*b*^ (%)
1	Co(OAc)_2_·4H_2_O	EtOAc	70	60	59
2	—	EtOAc	70	<1	<1
3	Co(OAc)_2_·4H_2_O	MeCN	70	50	45
4	Co(OAc)_2_·4H_2_O	AcOH	70	35	30
5	Co(OAc)_2_·4H_2_O	BuOAc	70	61	59
6	Co(NO_3_)_2_·6H_2_O	BuOAc	70	35	35
7	Co(acac)_2_	BuOAc	70	<1	<1
8	CoCl_2_·6H_2_O	BuOAc	70	14	14
9	Co(OAc)_2_·4H_2_O	BuOAc	80	95	89
**10**	**Co(OAc)**_**2**_ **·4H**_**2**_ **O**	**BuOAc**	**90**	**99**	**94**

^*a*^1 mmol scale, orbital mixing.

^*b*^GC yields with benzonitrile as an internal standard.

A comparison of the optimized Co/NHPI reaction conditions with previously reported benzylic oxygenation methods in Table 2 highlights the potential utility of the new conditions. Poor yields and mass balances were obtained using two Mid-Century-type radical autoxidation methods (entries 1 and 2),^14^ in which a bromine radical is proposed to participate in the H-atom abstraction step. We also tested representative catalyst systems^6*e*^ for “heterolytic” aerobic C–H oxygenation, corresponding to the methods noted in Scheme 2. As expected from the precedents, 3-ethylpyridine **1a** is not a viable substrate with these catalyst systems (entries 3 and 4).^15^

**Table 2:** Comparison of catalyst systems^*a*^

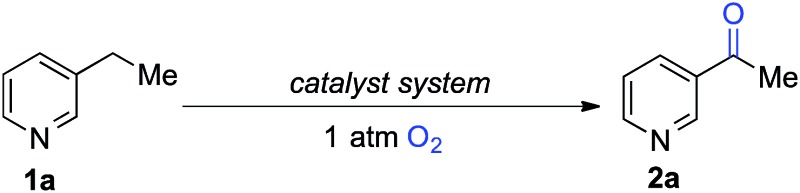				
Entry	Catalyst system	Ref.	Conv.^*b*^ (%)	Yield^*b*^ (%)
1	Co/Br^*c*^	14	51	19
2	Co/Mn/Br^*d*^	14	20	<1
3	CuI, 1 eq. AcOH^*e*^	6*e*	5	<1
4	FeCl_2_·4H_2_O, 1 eq. AcOH^*f*^	6*e*	7	<1
**5**	**Co/NHPI** ^*g*^	**This work**	**99**	**94**

^*a*^1 mmol scale, orbital mixing.

^*b*^GC yields with benzonitrile as an internal standard.

^*c*^1 mmol **1a**, 1 mL AcOH, 10 mol% Co(OAc)_2_·4H_2_O, 10 mol% HBr, 12 h, 100 °C.

^*d*^1 mmol **1a**, 1 mL AcOH, 5 mol% Co(OAc)_2_·4H_2_O, 5 mol% Mn(OAc)_2_·4H_2_O, 10 mol% HBr, 12 h, 100 °C.

^*e*^0.5 mmol **1a**, 1 mL DMSO, 10 mol% CuI, 1 eq. AcOH, 1 atm O_2_, 24 h, 100 °C.

^*f*^0.5 mmol **1a**, 1 mL DMSO, 10 mol% FeCl_2_·4H_2_O, 1 eq. AcOH, 24 h, 100 °C.

^*g*^1 mmol **1a**, 1 mL BuOAc, 1 mol% Co(OAc)_2_·4H_2_O, 20 mol% NHPI, 12 h, 90 °C.

The optimized reaction conditions were then tested for aerobic oxygenation of a number of pharmaceutically relevant (hetero)arene substrates (Table 3).^16^ In general, pyridines are well tolerated, with good-to-excellent yields obtained for products **2a–f**. This collection of substrates includes 3-substituted pyridine derivatives (**1a**, **1c**, **1f**), which are ineffective with heterolytic C–H oxygenation methods, as well as 2- and 4-substituted pyridines, which are compatible with the heterolytic methods. Excellent yields were obtained for the benzimidazole derivatives **1g** and **1h**. Ethylbenzene derivatives bearing remote heteroatom-containing functional groups, including a pyridyl, an imidazole and an acetamide group (**1i**, **1j** and **1k**), also underwent successful oxygenation.

**Table 3:** Oxygenation of polar (hetero)arenes^*a*^

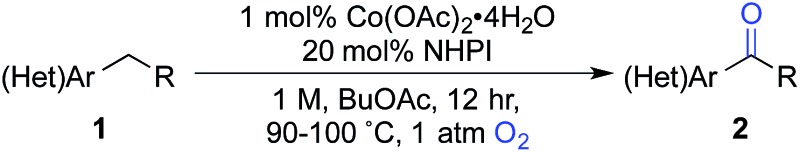
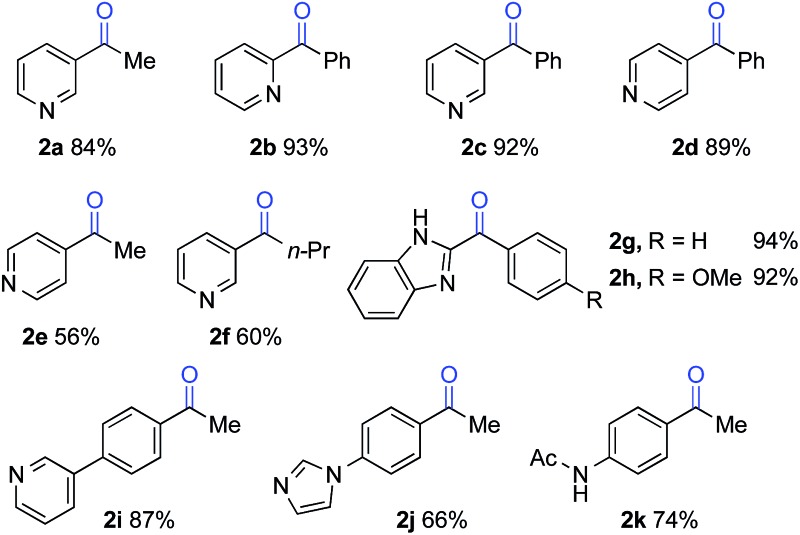

^*a*^1 mmol scale, isolated yields.

The standard reaction conditions in Table 3 proved to be ineffective for certain substrates,^17^ and two different approaches were identified to address some of these limitations. In the first case, the simple hydrocarbon, *n*-butylbenzene (**1l**) (Table 4), as well as the sulphur-containing heterocyclic substrates **1m**, **1n** and **1o** (Table 5) led to poor results (42%, 3%, 7% and 31% yields, respectively). In the reaction of **1l**, benzoic acid was observed as the major side product, arising from cleavage of the alkyl chain. Subsequent empirical studies showed that use of pyridine as a co-solvent attenuated this alkyl chain cleavage and enabled higher yields and mass balance in the oxygenation of **1l**. The optimal 7 : 3 BuOAc : pyr solvent mixture (77% yield of **2l**, entry 4) proved to be similarly beneficial for the oxygenation of the sulphur-heterocycle substrates **1m**, **1n** and **1o**, as well as the *N*-methylated benzimidazole **1p** (Table 5).^18^ The latter substrates underwent oxygenation in 65%, 50%, 72% and 94% yields, respectively. The reactions with pyridine co-solvent have a dark red color, consistent with pyridine coordination to cobalt.^19^ We speculate that this coordination might attenuate unproductive cobalt-mediated side reactions in these substrates.

**Table 4:** Enhanced selectivity for formation of **2l** in the presence of pyridine cosolvent^*a*^

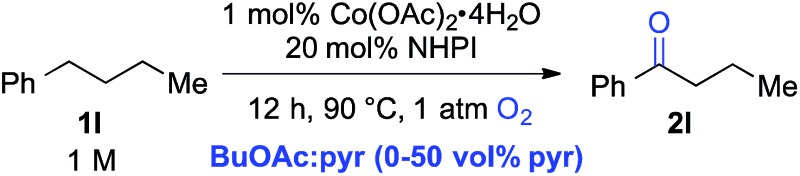			
Entry	Vol% pyr	Conv.^*b*^ (%)	Yield^*b*^ (%)
1	0	>99	42
2	10	98	74
3	20	95	76
**4**	**30**	**90**	**77**
5	40	84	73
6	50	74	66

^*a*^1 mmol scale, orbital mixing.

^*b*^GC yields with benzonitrile as an internal standard.

**Table 5:** Use of pyridine co-solvent for improved selectivity^*a*^

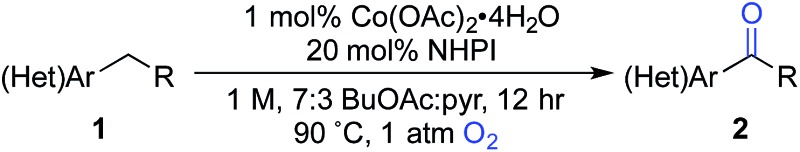
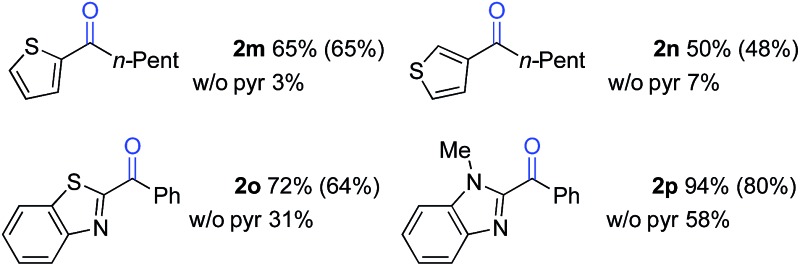

^*a*^1 mmol scale. Yields were determined by ^1^H NMR spectroscopy. Isolated yields in parentheses.

**Scheme 4:**
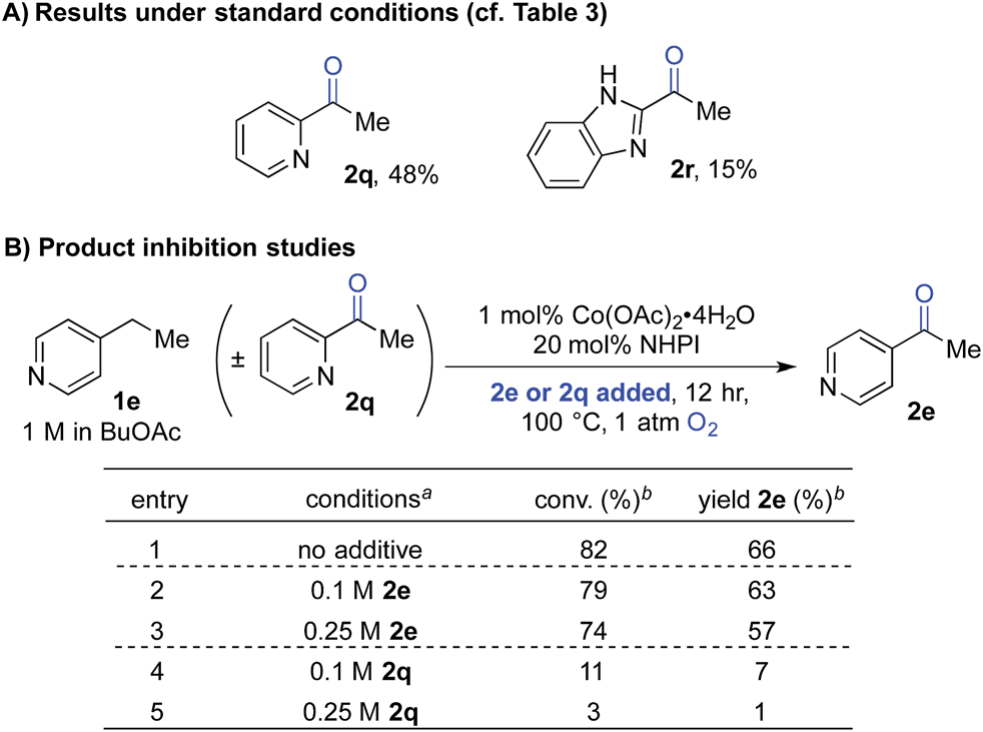
Product-inhibition studies in Co/NHPI-catalyzed oxygenation. ^*a*^1 mmol scale, orbital mixing. ^*b*^GC yields with chlorobenzene as an internal standard.

The second case involved 2-ethylpyridine (**1q**) and 2-ethylbenzimidazole (**1r**), in which the benzylic C–H bonds of the ethyl substituent is directly adjacent to a coordinating group. Under the standard conditions, the benzylic ketones **2q** and **2r** were obtained in only 48% and 15% yields, respectively (Scheme 4A). This poor reactivity contrasts the good reactivity observed with analogous doubly benzylic substrates **1b**, **1g** and **1h** in Table 3. We hypothesized that, in these reactions, the product could chelate the cobalt co-catalyst and inhibit further product formation. To test this hypothesis, we investigated the reaction of an effective substrate, 4-ethylpyridine **1e**, in the presence and absence of 4-acetylpyridine **2e** or 2-acetylpyridine **2q** (Scheme 4B). The results show that **2e** has minimal impact on the reaction (Scheme 4B, entries 1–3), whereas the presence of **2q** significantly lowers the yield in the oxygenation of **1e** (Scheme 4B, entries 1, 4 and 5).

**Scheme 5:**
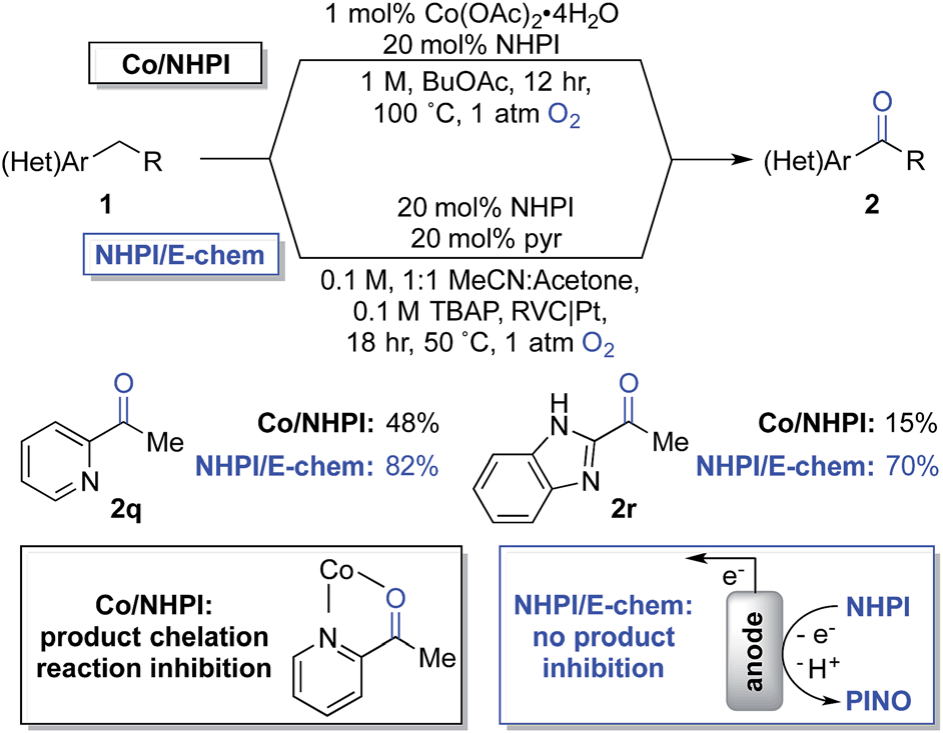
Overcoming a limitation in Co/NHPI chemistry through the electrochemical generation of PINO.

The observations in Scheme 4 prompted us to consider cobalt-free electrochemical aerobic oxygenation reactions, originally reported by Masui and coworkers in the 1980s.^20,21^ These methods generate PINO *via* electrochemical oxidation of NHPI (*i.e.*, in the absence of cobalt ions) and, therefore, should not be susceptible to chelate-inhibition by the product. Preliminary studies validated this hypothesis. Optimization of Masui's reported electrochemical conditions with **1q** and **1r** enabled formation of the ketone products **2q** and **2r** in 82% and 70% yields, respectively. These results represent substantial improvements over those obtained with the chemical conditions (Scheme 5). Further studies will be needed to explore the full scope and limitations of the electrochemical method. Preliminary studies with other substrates (*e.g.*, with **1b** and **1g**) suggest that the chemical conditions will be superior to the electrochemical conditions in other cases. These observations together with the ease of reaction set up and performance of the Co/NHPI/O_2_ oxygenations suggest that the aerobic reactions will be advantageous in most cases. Nonetheless, the results with the oxygenation of **1q** and **1r** demonstrate that electrochemical NHPI-mediated reactions could be a valuable complement to Co/NHPI reactions in strategic situations.

**Scheme 6:**
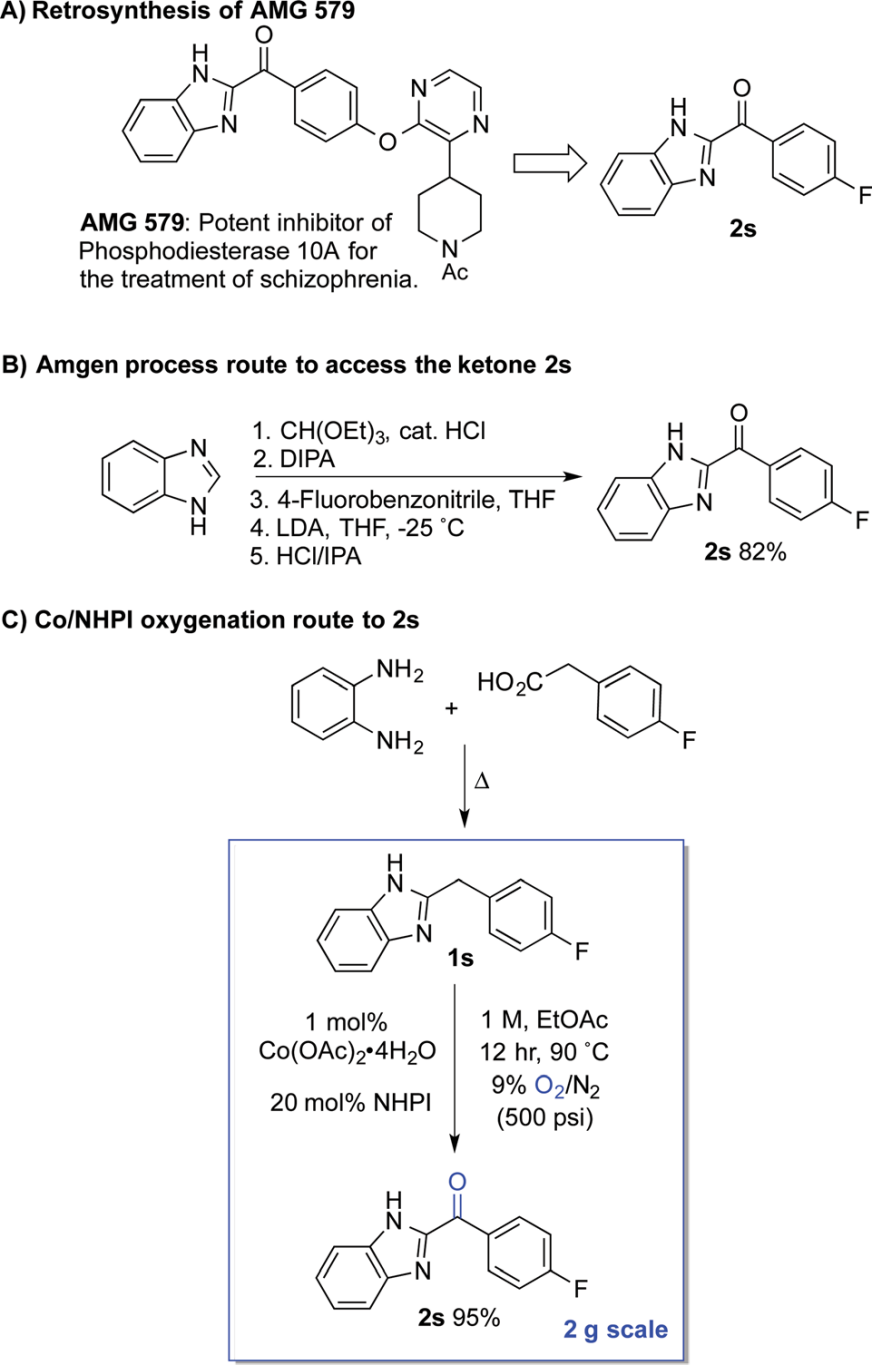
Streamlined synthetic route toward **AMG 579** *via* Co/NHPI-catalyzed benzylic oxygenation.

The standard Co/NHPI/O_2_ conditions developed here exhibit a number of features that are appealing from a process chemistry perspective, including a low cobalt catalyst loading and a high reaction concentration, which minimizes solvent volume. The relatively high NHPI loading (20 mol%) is offset by its extraordinarily low cost (<$5 per kg on commercial scale). In an effort to demonstrate the potential utility of this method for larger-scale applications, we targeted a streamlined route to the heterocyclic ketone **2s** (Scheme 6A), an intermediate *en route* to **AMG 579**.^22^ The latter compound is a clinical candidate for the treatment of neurological conditions such as schizophrenia and Huntington's disease. The reported multi-step route to **2s** is effective, but it is operationally complex and requires careful control of reaction temperature during the deprotonation and addition (Scheme 6B).^23^ We envisioned an alternative, two-step route to **2s**, starting from the inexpensive, commercially available precursors *o*-phenylene diamine and 4-fluorophenylacetic acid (Scheme 6C). The Co/NHPI-catalyzed oxygenation of **1s** was performed in EtOAc (1 M) with 500 psi of 9% O_2_ in N_2_ to maintain the O_2_ concentration below the limiting oxygen concentration (LOC) of EtOAc.^24^ Operation of this reaction on 2 g scale resulted in near quantitative isolated yield of **2r** (95%).

This route to **2s** incorporates a number established green chemistry principles:^25^ (i) waste prevention due to high volumetric efficiency, (ii) atom economy due to use of O_2_ as both the terminal oxidant and oxygen atom source, (iii) use of an environmentally benign ester solvent,^26^ (iv) use of an earth-abundant metal catalyst, (v) application of practical reaction conditions with respect to temperature and pressure, as well as (vi) application of inherently safe reaction conditions (*i.e.*, below the LOC of EtOAc). Although more thorough analysis would be required to compare the overall process viability of the two routes, the two-step condensation/oxidation sequence in Scheme 6C should have a much smaller environmental footprint, owing to the formation of water as a only stoichiometric by-product in each of the two reactions.^27^ Reflecting this feature, the route in Scheme 6B has an estimated *E*-factor of >15, while the two-step condensation/oxidation sequence can be <5.^28^

## Conclusions

The results described herein show that Co/NHPI-catalyzed, radical-mediated, benzylic aerobic oxygenation reactions are quite effective with pharmaceutically relevant heterocyclic substrates. The comparatively non-polar nature of the H-atom transfer step mediated by PINO plays an important role in expanding the scope of aerobic benzylic oxygenation to substrates that are ineffective with complementary heterolytic oxygenation methods.^5,6^ The chemistry and reaction conditions identified herein are sufficiently practical that these methods could be compelling for large-scale application. In addition to the low *E*-factors associated with these reactions, the homogeneous reaction conditions (*i.e.*, lacking solid reagents or additives) suggest that they are excellent candidates for continuous-flow applications.^29^ This opportunity warrants attention in future studies.
