# Supplementary material for: Co/NHPI-mediated aerobic oxygenation of benzylic C–H bonds in pharmaceutically relevant molecules
Source: Chem Sci. 2016 Oct 7;8(2):1282–7. doi: 10.1039/c6sc03831j (PMC5359875; doi:10.1039/c6sc03831j)
Supplement: Supplementary file 1 [file SC-008-C6SC03831J-s001.pdf]

Electronic Supplementary Material (ESI) for Chemical Science.
This journal is © The Royal Society of Chemistry 2016

Supporting Information

Co/NHPI-mediated aerobic oxygenation of benzylic C–H bonds in pharmaceutically relevant molecules

Damian P. Hruszkewycz, Kelsey C. Miles, Oliver R. Thiel, and Shannon S. Stahl

Department of Chemistry, University of Wisconsin–Madison, 1101 University Avenue, Madison, WI, 53706, United States

Table of Contents

General considerations 2
General procedure for reaction optimization (Tables 1, S1, and S2) 2
Table S1. Optimization of reaction conditions: full screening data 3
Table S2. Optimization of NHPI loading. 4
Procedures for comparison of catalyst systems (Table 2) 4
General procedures for the oxygenation of (hetero)arenes (Table 3) 5
Procedure to explore the effect of pyridine on butylbenzene oxygenation (Tables 4 and S3) 5
Table S3. Butylbenzene oxygenation with pyridine cosolvent: full screening data 6
Procedure for comparing catalysis in BuOAc vs. 7:3 BuOAc:pyr (Tables 5 and S4) 6
Procedure to study the inhibition of Co/NHPI catalysis by 2-acetylpyridine (Scheme 4) 7
Procedures for the oxygenation of 1q and 1r (Schemes 4 and 5) 7
Table S4. Representative unsuccessful substrates. 8
Table S5. Robustness screen 9
Competition experiment for oxygenation of 3-benzylpyridine vs. 3-ethylpyridine 10
Synthesis and characterization of substrates 11
Characterization data for isolated products 13
Multi-gram synthesis of 2-(4-fluorobenzoyl)benzimidazole (2s, Scheme 5) 19
E-factor analysis 20
References 23
^{1}H NMR, ^{13}C NMR, and ^{19}F NMR spectra 24

General considerations

All commercially available organic compounds and solvents were used as received from Sigma Aldrich, Alfa-Aesar, and TCI America unless otherwise specified. The following compounds and solvents were purchased from other vendors: DMSO-d_6 (Cambridge Isotopes). $Co(OAc)_2 \cdot 4H_2O$ (Sigma, ≥ 98.0%) was ground into a fine powder with a mortar and pestle prior to use. ^{1}H, ^{13}C, and ^{19}F NMR spectra were recorded on Bruker 400 and 500 MHz spectrometers. Chemical shifts are given in parts per million (ppm) relative to residual solvent peaks in the ^{1}H and ^{13}C NMRs and relative to $CFCl_3$ in the ^{19}F NMR. High-resolution mass spectra were obtained using a Thermo Q Exactive™ Plus by the mass spectrometry facility at the University of Wisconsin. Chromatography was performed using an automated Isco Combiflash Rf® system with silica gel columns (Silicycle, particle size 40-63 µm, 230-400 mesh). Gas chromatography analyses were performed on a Shimadzu gas chromatograph (GC-2010 Plus) using a Beta DEXTM 225 fused silica capillary column (30 m x 0.25 mm x 0.25 µm film thickness) with a flame ionization detector.

General procedure for reaction optimization (Tables 1, S1, and S2)

A test tube was charged with a transition metal salt (0.01 mmol), *N*-hydroxyphthalimide (0.05 mmol-0.2 mmol), and a 1 M 3-ethylpyridine or 4-ethylpyridine solution in the appropriate solvent (1 mL). The test tube was placed in a parallel orbital-mixing "shaker" reactor pre-set to the given reaction temperature. The headspace was purged with O_2 for 5 min under orbital shaking (50 rpm). The reactor was sealed under an O_2 pressure slightly above 1 atm, and the reaction was subjected to orbital shaking for an additional 12 h. The test tube was removed from the reactor, and benzonitrile (103 µL, 1 mmol) was added to the reaction mixture as an external standard for GC analysis. An aliquot of the reaction mixture was diluted into EtOAc and analyzed by calibrated GC.

Table S1. Optimization of reaction conditions: full screening data.[a]

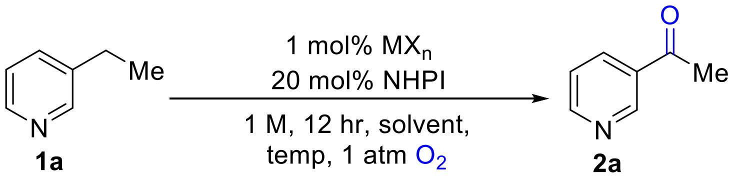

entry	MX_2	solvent	temp (ºC)	conv. (%)[b]	yield (%)[b]
1	$Co(OAc)_2•4H_2O$	EtOAc	70	60	59
2	--	EtOAc	70	<1	<1
3	$Co(OAc)_2•4H_2O$	MeCN	70	50	45
4	$Co(OAc)_2•4H_2O$	AcOH	70	35	30
5	$Co(OAc)_2•4H_2O$	BuOAc	70	61	59
6	$Co(OBz)_2$	BuOAc	70	59	57
7	$Co(Ethylhexanoate)_2$	BuOAc	70	60	58
8	$Co(NO_3)_2•6H_2O$	BuOAc	70	35	35
9	$Co(acac)_2$	BuOAc	70	<1	<1
10	$CoCl_2•6H_2O$	BuOAc	70	14	14
11	Cu(OAc)	BuOAc	70	5	4
12	$Cu(OAc)_2•H_2O$	BuOAc	70	5	5
13	$Mn(OAc)_2•4H_2O$	BuOAc	70	1	1
14	$Ni(OAc)_2•4H_2O$	BuOAc	70	<1	<1
15	$Fe(OAc)_2$	BuOAc	70	5	5
16	$Cr_3(OAc)_7(OH)_2$	BuOAc	70	1	1
17	$Co(OAc)_2•4H_2O$	BuOAc	80	95	89
18	**$Co(OAc)_2•4H_2O$**	**BuOAc**	**90**	**99**	**94**
19	$Co(OAc)_2•4H_2O$	BuOAc	100	98	93

[a]1 mmol scale, orbital mixing. [b]Evaluated using GC-FID with benzonitrile as an external standard.

Table S2. Optimization of NHPI loading.[a]

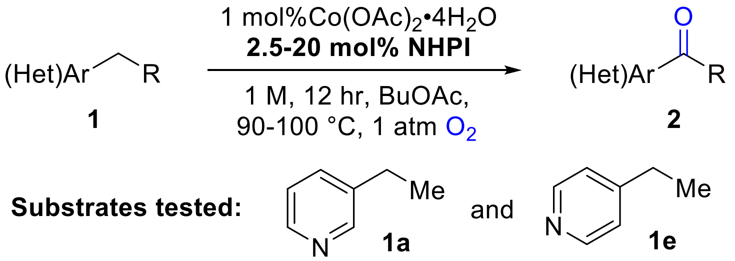

entry	substrate	mol% NHPI	temp (°C)	conv. (%)[b]	yield (%)[b]
1	**1a**	2.5	90	<1	<1
2	**1a**	5	90	63	62
3	**1a**	10	90	94	88
4	**1a**	20	90	99	88
5	**1e**	2.5	90	7	5
6	**1e**	5	90	34	29
7	**1e**	10	90	58	48
8	**1e**	20	90	73	60
9	**1e**	2.5	100	5	5
10	**1e**	5	100	34	30
11	**1e**	10	100	62	52
12	**1e**	20	100	81	66

[a]1 mmol scale, orbital mixing. [b]Evaluated using GC-FID with benzonitrile as an external standard.

Procedures for comparison of catalyst systems (Table 2)

Entry 1: A test tube was charged with $Co(OAc)_2 \cdot 4H_2O$ (24.9 mg, 0.1 mmol), 1 M HBr in AcOH (100 µL, 0.1 mmol HBr), and 1.11 M 3-ethylpyridine in AcOH (900 µL, 1 mmol). The test tube was placed in a parallel shaker reactor pre-set to 100 °C. The headspace was purged with O_2 for 5 min under orbital shaking (50 rpm). The reactor was sealed under an O_2 pressure slightly above 1 atm, and the reaction was subjected to orbital shaking for an additional 12 h. The test tube was removed from the reactor, and benzonitrile (103 µL, 1 mmol) was added to the reaction mixture as an external standard for GC analysis. An aliquot of the reaction mixture was diluted into EtOAc and analyzed by calibrated GC.

Entry 2: A test tube was charged with $Co(OAc)_2 \cdot 4H_2O$ (12.5 mg, 0.05 mmol), $Mn(OAc)_2 \cdot 4H_2O$ (12.2 mg, 0.05 mmol), 1 M HBr in AcOH (100 µL, 0.1 mmol HBr), and 1.11 M 3-ethylpyridine in AcOH (900 µL, 1 mmol). The test tube was placed in a parallel shaker reactor pre-set to 100 °C. The headspace was purged with O_2 for 5 min under orbital shaking (50 rpm). The reactor was sealed under an O_2 pressure slightly above 1 atm, and the reaction was subjected to orbital shaking for an additional 12 h. The test tube was removed from the reactor, and benzonitrile (103 µL, 1 mmol) was added to the reaction mixture as an external standard for GC analysis. An aliquot of the reaction mixture was diluted into EtOAc and analyzed by calibrated GC.

Entries 3 and 4: A test tube was charged with the appropriate metal salt (0.05 mmol), AcOH (29 µL, 0.5 mmol), 3-ethylpyridine (53.6 mg, 0.5 mmol), and DMSO (1 mL). The test tube was placed in a parallel shaker reactor pre-set to 100 °C. The headspace was purged with O_2 for 5 min under orbital shaking (50 rpm). The reactor was sealed under an O_2 pressure slightly above 1 atm, and the reaction was subjected to orbital shaking for an additional 12 h. The test tube was removed from the reactor, and benzonitrile (51.5 µL, 0.5 mmol) was added to the reaction mixture as an external standard for GC analysis. An aliquot of the reaction mixture was diluted into EtOAc and analyzed by calibrated GC.

General procedures for the oxygenation of (hetero)arenes (Table 3)

Most of the substrates were reacted in test tubes under orbital mixing:
A test tube was charged with $Co(OAc)_2{\cdot}4H_2O$ (2.5 mg, 0.0l mmol), *N*-hydroxyphthalimide (32.6 mg, 0.2 mmol), substrate (1 mmol), and BuOAc (1 mL). The test tube was placed in a parallel shaker reactor pre-set to 90 or 100 °C. The headspace was purged with O_2 for 5 min under orbital shaking (50 rpm). The reactor was sealed under an O_2 pressure slightly above 1 atm, and the reaction was subjected to orbital shaking for an additional 12 h.

*Substrates **1c** and **1g** were reacted in a vial to demonstrate the utility of these reaction conditions using common benchtop equipment:*
A disposable 4 mL vial was charged with $Co(OAc)_2{\cdot}4H_2O$ (2.5 mg, 0.0l mmol), *N*-hydroxyphthalimide (32.6 mg, 0.2 mmol), substrate (1 mmol), and BuOAc (1 mL). The vial was sealed with a PTFE-lined pierceable cap. The headspace of the vial was purged with an O_2-filled balloon for 2 min. The vial was transferred to a heating block pre-set to 90 or 100 °C with the O_2-filled balloon still attached, and the reaction mixture was vigorously stirred for 12 hr.

General workup procedure:
After cooling to rt, the reaction mixture was transferred to a separatory funnel using EtOAc (15 mL) to ensure quantitative transfer. The separatory funnel was also charged with 25% aqueous NaOH (1.5 mL) and Bu_4NBr (5 mg), and the contents were vigorously shaken for 6 min. After this initial treatment, H_2O (4 mL) was added, and the contents were vigorously shaken for an additional 1 min. The organic layer was separated, and the aqueous layer was extracted with EtOAc (2 x 15 mL). The combined organic layers were dried over $MgSO_4$, and the volatiles were removed under vacuum. The resultant residue was purified through column chromatography.

Procedure to explore the effect of pyridine on butylbenzene oxygenation (Tables 4 and S3)

A test tube was charged with $Co(OAc)_2{\cdot}4H_2O$ (2.5 mg, 0.0l mmol), *N*-hydroxyphthalimide (32.6 mg, 0.2 mmol), 2 M butylbenzene in BuOAc (500 µL), and the corresponding volumes of BuOAc (0-500 µL) and pyridine (0-500 µL). The test tube was placed in a parallel shaker reactor pre-set to 90 or 100 °C. The headspace was purged with O_2 for 5 min under orbital shaking (50 rpm). The reactor was sealed under an O_2 pressure slightly above 1 atm, and the reaction was subjected to orbital shaking for an additional 12 h. The test tube was removed from the reactor, and benzonitrile (103 µL, 1 mmol) was added to the reaction mixture as an external standard for GC analysis. An aliquot of the reaction mixture was diluted into EtOAc and analyzed by calibrated GC.

Table S3. Butylbenzene oxygenation with pyridine cosolvent: full screening data.[a]

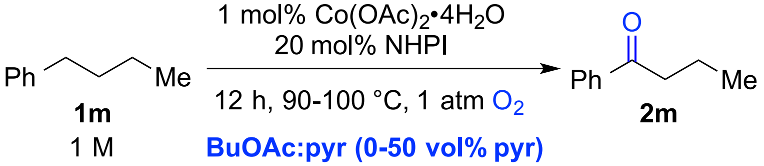

entry	Temp (°C)	vol% pyr	conv. (%)[b]	yield (%)[b]
1	90	0	>99	42
2	90	10	98	74
3	90	20	95	76
4	90	25	93	77
**5**	**90**	**30**	**90**	**77**
6	90	33	88	75
7	90	40	84	73
8	90	50	74	66
9	100	0	90	42
10	100	10	97	69
11	100	20	96	73
12	100	25	95	73
13	100	30	94	76
14	100	33	91	74
15	100	40	86	73
16	100	50	82	71

[a]1 mmol scale, orbital mixing. [b]Evaluated using GC-FID with benzonitrile as an external standard.

Procedure for comparing catalysis in BuOAc vs. 7:3 BuOAc:pyr (Tables 5 and S4)

A test tube was charged with $Co(OAc)_2 \cdot 4H_2O$ (2.5 mg, 0.01 mmol), *N*-hydroxyphthalimide (32.6 mg, 0.2 mmol), substrate (1 mmol), and either 7:3 BuOAc:pyr or 100% BuOAc (1 mL). The test tube was placed in a parallel shaker reactor pre-set to 90 °C, and the headspace was purged with O_2 for 5 min under orbital shaking (50 rpm). The reactor was sealed under an O_2 pressure slightly above 1 atm, and the reaction was subjected to orbital shaking for an additional 12 h.

^{1}H NMR yields were obtained after this reaction period using the following procedure:
The reaction mixture was charged with either 1,3,5-trimethoxybenzene (56.1 mg, 0.33 mmol) or diphenylmethane (83.6 µL, 0.50 mmol) as an external standard. An aliquot was removed from the mixture and filtered through a small plug of silica gel using 1:1 EtOAc:DCM as an eluent. The volatiles were removed from the filtrate under vacuum. The resultant residue was dissolved in $CDCl_3$ or $(CD_3)_2SO$ and analyzed by ^{1}H NMR (relaxation time = 25 s).

Isolated yields for the reactions in 7:3 BuOAc:pyr were obtained using the following procedure:
The reaction mixture and the ^{1}H NMR sample were transferred to a separatory funnel using EtOAc (15 mL) to ensure quantitative transfer. The separatory funnel was also charged with 25%

aqueous NaOH (1.5 mL) and Bu_4NBr (5 mg), and the contents were vigorously shaken for 6 min. After this initial treatment, H_2O (4 mL) was added, and the contents were vigorously shaken for an additional 1 min. The organic layer was separated, and the aqueous layer was extracted with EtOAc (2 x 15 mL). The combined organic layers were dried over $MgSO_4$, and the volatiles were removed under vacuum. The resultant residue was purified through column chromatography.

Procedure to study the inhibition of Co/NHPI catalysis by 2-acetylpyridine (Scheme 4)

Entry 1: A test tube was charged with $Co(OAc)_2{\cdot}4H_2O$ (2.5 mg, 0.0l mmol), *N*-hydroxyphthalimide (32.6 mg, 0.2 mmol), 2 M 4-ethylpyridine in BuOAc (500 μL), and BuOAc (500 μL).

Entries 2-3: A test tube was charged with $Co(OAc)_2{\cdot}4H_2O$ (2.5 mg, 0.0l mmol), *N*-hydroxyphthalimide (32.6 mg, 0.2 mmol), 2 M 4-ethylpyridine in BuOAc (500 μL), and the corresponding volumes of 1 M 4-acetylpyridine in BuOAc (100 μL or 250 μL) and BuOAc (400 μL or 250 μL).

Entries 4-5: A test tube was charged with $Co(OAc)_2{\cdot}4H_2O$ (2.5 mg, 0.0l mmol), *N*-hydroxyphthalimide (32.6 mg, 0.2 mmol), 2 M 4-ethylpyridine in BuOAc (500 μL), and the corresponding volumes of 1 M 2-acetylpyridine in BuOAc (100 μL or 250 μL) and BuOAc (400 μL or 250 μL).

Entries 1-5: The test tube with composition defined above was placed in a parallel shaker reactor pre-set to 100 °C. The headspace was purged with O_2 for 5 min under orbital shaking (50 rpm). The reactor was sealed under an O_2 pressure slightly above 1 atm, and the reaction was subjected to orbital shaking for an additional 12 h. The test tube was removed from the reactor, and chlorobenzene (108 μL, 1 mmol) was added to the reaction mixture as an external standard for GC analysis. An aliquot of the reaction mixture was diluted into EtOAc and analyzed by calibrated GC.

Procedures for the oxygenation of 1q and 1r (Schemes 4 and 5)

Co/NHPI oxygenation:
A test tube was charged with $Co(OAc)_2{\cdot}4H_2O$ (2.5 mg, 0.0l mmol), *N*-hydroxyphthalimide (32.6 mg, 0.2 mmol), substrate (1 mmol), BuOAc (1 mL). The test tube was placed in a parallel shaker reactor pre-set to 100 °C, and the headspace was purged with O_2 for 5 min under orbital shaking (50 rpm). The reactor was sealed under an O_2 pressure slightly above 1 atm, and the reaction was subjected to orbital shaking for an additional 12 h. The reaction mixture was charged with either 1,3,5-trimethoxybenzene (56.1 mg, 0.33 mmol) as an external standard. An aliquot was removed from the mixture and filtered through a small plug of silica gel using 1:1 EtOAc:DCM as an eluent. The volatiles were removed from the filtrate under vacuum. The resultant residue was dissolved in $CDCl_3$ or $(CD_3)_2SO$ and analyzed by ^1H NMR (relaxation time = 25 s).

General procedure for the electrochemical aerobic oxidation heterocyclic methylenes:

Bulk electrolysis experiments were performed in a 20 mL undivided cell equipped with a teflon stir bar, two blocks of reticulated vitreous carbon (RVC) as the working electrode, a platinum wire counter electrode, and a Ag/Ag+ reference electrode. The oxidation was performed at the oxidative peak potential of NHPI and was determined by measuring the oxidation of NHPI under these reaction conditions with a glassy carbon disk electrode. The cell was charged with heterocyclic methylene substrate (1mmol), *N*-hydroxyphthalimide (32.6 mg, 0.2 mmol), pyridine (16.2 µL, 0.2 mmol), CH_3CN (5 mL), acetone (5 mL), and tetrabutylammonium perchlorate (341 mg, 1 mmol). The reaction was purged with oxygen, capped with a septum and fitted with a balloon of oxygen. The reaction was heated to 50 °C and the oxidative potential was applied (0.65-0.75 V) overnight (18 h) while stirring. The crude mixture was concentrated and the product was isolated by column chromatography on silica gel. It should be noted that careful washing of the RVC was necessary to ensure all of the product was collected.

Table S4. Representative unsuccessful substrates.[a]

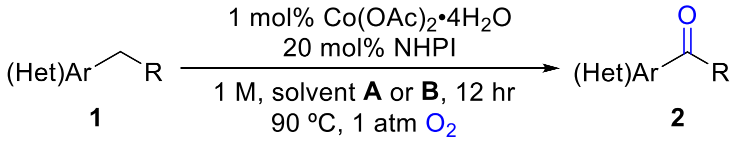

Solvents: **A:** 100% BuOAc; **B:** 7:3 BuOAc:pyr

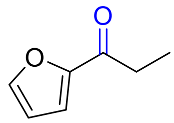	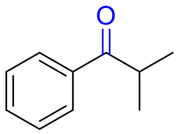	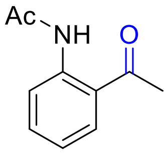
**A:** 0% (conv. >99%)	**A:** 26% (conv. >99%)	**A:** 31% (conv. 47%)
**B:** 7% (conv. >99%)	**B:** 41% (conv. >99%)	**B:** 9% (conv. 9%)

[a]1 mmol scale. Yields and conversions were determined by ^{1}H NMR spectroscopy.

Commentary on these unsuccessful reactions:
2-Propylfuran: A complex mixture of byproducts was observed in both reactions, and these byproducts were not further analyzed.

Isobutylbenzene: The major byproduct in both solvents is benzoic acid: (64% yield in BuOAc; 43% yield in 7:3 BuOAc:pyr). Since benzoic acid formation is more prevalent in the reactions of isobutylbenzene, compared to butylbenzene (Table 4), we propose that increasing substitution at the position next to the benzylic position renders alkyl cleavage more facile.

2-Ethylacetanilide: We hypothesize that the poor yields in these reactions area a result of product inhibition resulting from chelation of the 2-acetylacetanilide product to the Co center, as was proposed for **2q** and **2r** in Scheme 5.

Table S5. Robustness screen.[a]

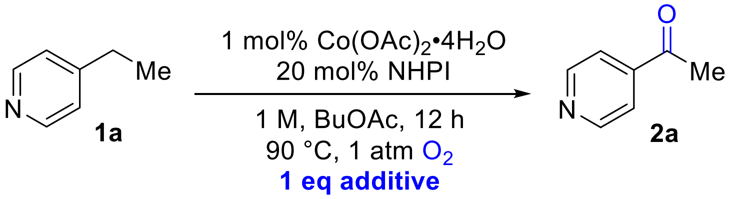

entry	additive	conv. (%)[b]	yield (%)[b]	additive remaining (%)[b]
1	--	98	89	--
2	AcOH	96	81	115
3	BuOH	74	67	<1
4	*N,N*-dimethylaniline	<1	<1	<1
5	*N*-methylindole	7	5	64
6	4-*tert*-butylstyrene	1	<1	63
7	phenylboronic acid pinacol ester	25[c]	<1[c]	99[c]
8	bromobenzene	98	81	>99
9	iodobenzene	40	34	>99
10	pyridine *N*-oxide (0.1 eq)	77	72	--

[a]1 mmol scale, orbital mixing. [b]Evaluated using GC-FID with benzonitrile as an external standard. [c]Evaluated by ^1H NMR.

General procedure for robustness screen:

A test tube was charged with $Co(OAc)_2 \cdot 4H_2O$ (2.5 mg, 0.01 mmol), *N*-hydroxyphthalimide (32.6 mg, 0.2 mmol), additive (1 mmol), 1 M 3-ethylpyridine in BuOAc (1 mL, 1 mmol). The test tube was placed in a parallel orbital-mixing "shaker" reactor pre-set to 90 °C. The headspace was purged with O_2 for 5 min under orbital shaking (50 rpm). The reactor was sealed under an O_2 pressure slightly above 1 atm, and the reaction was subjected to orbital shaking for an additional 12 h. The test tube was removed from the reactor, and benzonitrile (103 μL, 1 mmol) was added to the reaction mixture as an external standard for GC analysis. An aliquot of the reaction mixture was diluted into EtOAc and analyzed by calibrated GC.

Commentary on the robustness screen:

Entries 2-3: A small amount of AcOH is visible at the end of the reaction time in the Co/NHPI reactions, which were run at 90 and 100 °C and were analyzed by GC. Presumably, the AcOH forms from hydrolysis of the BuOAc solvent (we thank a reviewer for pointing out this possibility). The effect of exogenous AcOH and BuOH—the products of BuOAc hydrolysis—was therefore explored in Entries 2-3. Addition of exogenous AcOH has a very minor effect on catalysis, and the 115% yield of remaining additive is consistent with additional hydrolysis of BuOAc during the reaction to form additional AcOH. Addition of BuOH slows down catalysis, and the BuOH signal is not detectably by GC at the end of the reaction.

Entries 4-9: All of the functional groups tested, except the aryl bromide, shut down the Co/NHPI-mediated oxygenation of 3-ethylpyridine.

Entry 10: One reviewer suggested the possibility that some pyridine *N*-oxide might form during the reaction. A substoichiometric amount of pyridine *N*-oxide was added to explore the effect of possible *N*-oxide byproducts/intermediates. While the reaction did not go to completion, good conversion and mass balance was still observed.

Competition experiment for oxygenation of 3-benzylpyridine vs. 3-ethylpyridine

Table S6. Competition experiment.

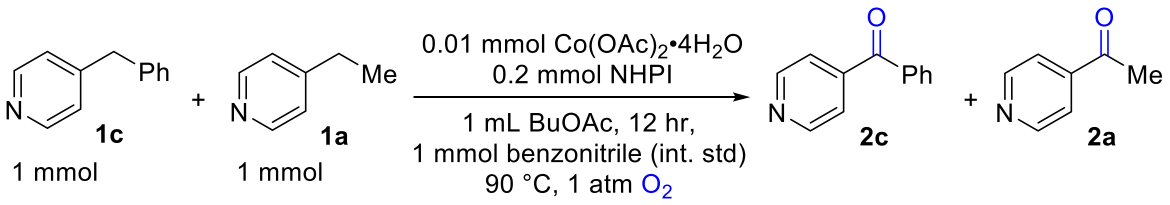

Time (h)	% **1c**[a]	% **2c**[a]	% **1a**[a]	% **2a**[a]
1	39	65	84	15
2	18	83	73	26
3	7	94	62	36
4	3	97	52	46
5	1	99	43	54
6	0.4	99	36	61
7	0.2	99	29	67
8	0.1	99	23	72

[a]Evaluated using GC-FID with benzonitrile as an external standard.

Figure S1. Time course of competition experiment in Table S6.

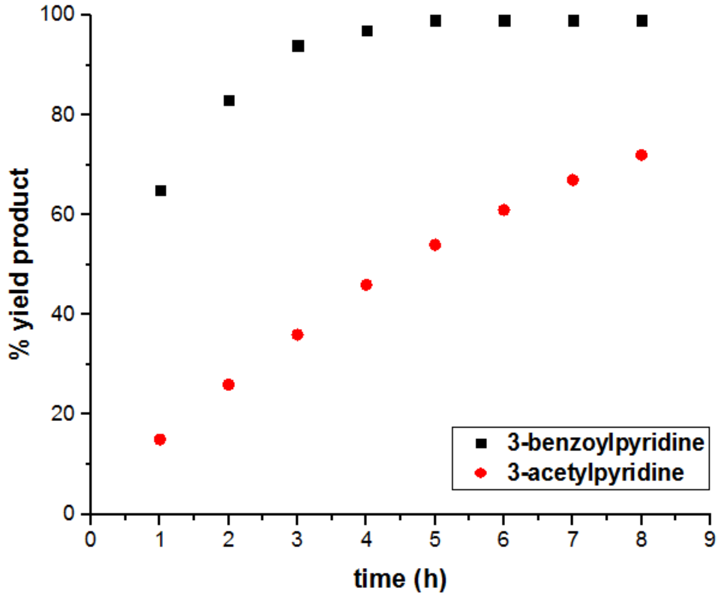

Procedure for competition experiment:
A test tube was charged with $Co(OAc)_2·4H_2O$ (2.5 mg, 0.01 mmol), *N*-hydroxyphthalimide (32.6 mg, 0.2 mmol), 3-benzylpyridine (173.6 mg, 1.03 mmol), 3-ethylpyridine (108.8 mg, 1.02 mmol), benzonitrile (102.5 mg, 0.99 mmol), and BuOAc (1 mL). The test tube was placed in a parallel orbital-mixing "shaker" reactor pre-set to 90 °C. The headspace was purged with O_2 for 5 min under orbital shaking (50 rpm). The reactor was sealed under an O_2 pressure slightly above 1 atm, and the reaction was subjected to orbital shaking for an additional 8 h. For each time point, the orbital-mixing was briefly paused, and a small aliquot of the homogeneous reaction mixture was removed using a syringe. This aliquot was diluted into EtOAc and analyzed by calibrated GC.

Synthesis and characterization of substrates

Substrates **1a**, **1b**, **1c**, **1d**, **1e**, **1f**, **1g**, **1l**, **1m**, **1n**, **1q**, and **1r** were purchased from commercial vendors. Substrates **1g**,[1] **1h**,[1] **1k**,[2] and **1o**[3] were prepared according to literature reports. Substrates that were not prepared via procedures reported in the literature are presented below. None of these procedures were optimized to maximize yield.

3-(4-Ethylphenyl)pyridine (1i)[4]

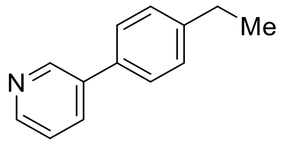

This procedure was adapted from reaction conditions that were previously reported:[5]
Under air, a Schlenk tube was charged with a magnetic stir bar, 3-bromopyridine (480 µL, 5.0 mmol), 4-ethylphenylboronic acid (825 mg, 5.5 mmol), sodium *tert*-butoxide (720 mg, 7.5 mmol), allyl[1,3-bis(mesityl)imidazol-2-ylidene]chloropalladium(II) (49 mg, 0.1 mmol), and dioxane (20 mL). The Schlenk tube was quickly sealed, and the reaction mixture was freeze-pump-thawed twice. After this, an N_2 atmosphere was introduced to the Schlenk tube, and the reaction mixture was stirred at 80 °C for 3 h. After cooling to rt, the reaction mixture was diluted into 40 mL EtOAc and washed with H_2O (2 x 10 mL) in a separatory funnel. The organic layer was dried over $MgSO_4$, and the volatiles were removed under vacuum. The resultant residue was purified through column chromatography (10→40% EtOAc/Hexanes, 1% triethylamine) to yield 3-(4-ethylphenyl)pyridine (467 mg, 2.55 mmol, 51% yield) as a pale yellow oil.
^{1}H NMR (500 MHz, C_6D_6) δ 9.00 (d, *J* = 1.8 Hz, 1H), 8.54 (dd, *J* = 4.7, 1.6 Hz, 1H), 7.43 – 7.37 (m, 1H), 7.26 (d, *J* = 8.2 Hz, 2H), 7.02 (d, *J* = 8.3 Hz, 2H), 6.78 (ddd, *J* = 7.9, 4.8, 0.7 Hz, 1H), 2.44 (q, *J* = 7.6 Hz, 2H), 1.09 (t, *J* = 7.6 Hz, 3H). **^{13}C NMR** (126 MHz, C_6D6) δ 148.88, 148.77, 144.15, 136.68, 135.85, 133.71, 128.80, 127.41, 123.46, 28.83, 15.77.

1-(4-Ethylphenyl)-1*H*-imidazole (1j)[6]

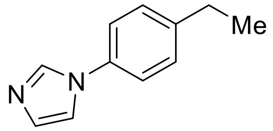

This procedure was adapted from reaction conditions that were previously reported:[7]
Under air, a Schlenk tube was charged with a magnetic stir bar, 1-bromo-4-ethylbenzene (924 mg, 4.99 mmol), imidazole (443 mg, 6.5 mmol), K_2CO_3 (760 mg, 5.5 mmol), *N*-methypyrrolidine (1 mL), and CuBr (18 mg, 0.13 mmol). The Schlenk tube was quickly sealed, and the reaction mixture was freeze-pump-thawed twice. After this, an N_2 atmosphere was introduced to the Schlenk tube, and the reaction mixture was stirred at 160 °C for 16 h. After cooling to rt, the reaction mixture was diluted into 40 mL EtOAc under air and washed with H_2O (2 x 10 mL) in a separatory funnel. The volatiles were removed under vacuum, and the resultant residue was dissolved in 0.5 M aqueous HCl (16 mL). The aqueous mixture was washed with hexanes (2 x 20 mL) in a separatory funnel, and the organic layers were discarded. The aqueous layer was next basified to a pH of ~10 using aqueous NaOH and extracted with EtOAc (2 x 20 mL). The combined organic layers were dried over $MgSO_4$, and the volatiles were removed under vacuum. The resultant residue was then purified through column chromatography (2% triethylamine in DCM) to yield 1-(4-Ethylphenyl)-1*H*-imidazole (628 mg, 3.65 mmol, 73% yield) as a pale yellow oil.
^{1}H NMR (500 MHz, $CDCl_3$) δ 7.82 (s, 1H), 7.30 (s, 4H), 7.25 (s, 1H), 7.19 (s, 1H), 2.70 (q, *J* = 7.6 Hz, 2H), 1.27 (t, *J* = 7.6 Hz, 3H). **^{13}C NMR** (126 MHz, $CDCl_3$) δ 143.94, 135.81, 135.32, 130.37, 129.33, 121.71, 118.52, 77.41, 28.50, 15.69.

1-methyl-2-(benzyl)benzimidazole (1p)[8]

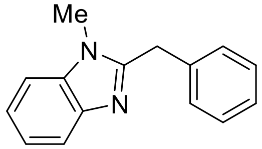

This procedure was adapted from reaction conditions that were previously reported:[9]
Under air, a round bottom flask was charged with a magnetic stir bar, 2-benzylbenzimidazole (1.045 g, 5.02 mmol), 50% aqueous NaOH (2 mL), MeI (340 µL, 5.5 mmol), and DMSO (1 mL). The flask was submerged into a warm water bath set to 40 °C, and the reaction mixture was stirred vigorously for 20 min. After this, the mixture was treated in a separatory funnel with EtOAc (50 mL) and H_2O (50 mL). The organic layer was dried over MgSO4, and the volatiles were removed under vacuum. The crude residue was purified through column chromatography (25→0% EtOAc/hexanes, 1% triethylamine) to yield 1-methyl-2-(benzyl)benzimidazole (799 mg, 3.59 mmol, 72% yield) as a white powder.
^{1}H NMR (500 MHz, $CDCl_3$) δ 7.80–7.74 (m, 1H), 7.32-7.20 (m, 8H), 4.33 (s, 2H), 3.58 (s, 3H). **^{13}C NMR** (126 MHz, $CDCl_3$) δ 153.39, 142.70, 136.31, 136.22, 128.97, 128.57, 127.05, 122.41, 122.04, 119.64, 109.13, 34.64, 30.19.

2-(4-Fluorobenzyl)benzimidazole (1s)

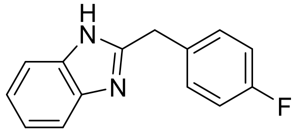

This procedure, adapted from previously reported reaction conditions,[1] was not optimized. A more-appealing protocol for the preparation of this compound was later identified but not used in the present study (see ref.12):

A round bottom flask was charged with a magnetic stir bar, *o*-phenylenediamine (10.8 g, 100 mmol), and 4-fluorophenylacetic acid (15.423 g, 100 mmol), and 5 N HCl (350 mL). A reflux condenser was attached, and the headspace was purged with N_2 for 5 min. The reaction mixture was stirred under reflux for 18 h. After cooling to rt, the reaction mixture was poured into cold water and neutralized with $NaHCO_3$. The resultant precipitate was isolated through filtration and recrystallized from hot ethanol/H_2O to yield 2-(4-fluorobenzyl)benzimidazole (5.04 g, 22.3 mmol, 22% yield) as an off-white solid.

^{1}H NMR (500 MHz, DMSO-d_6) 12.26 (s, 1H), 7.56-7.40 (br s, 2 H), 7.39-7.34 (m, 2H), 7.17-7.09 (m, 4H), 4.16 (s, 2H). **^{13}C NMR** (126 MHz, DMSO-d_6) δ 161.03 (d, J = 242.1 Hz), 153.40, 143.42, 134.36, 133.77 (d, J = 3.1 Hz), 130.64 (d, J = 8.1 Hz), 121.52, 121.08, 118.33, 115.16 (d, J = 21.2 Hz), 110.99, 34.03. **^{19}F NMR** (377 MHz, $CDCl_3$) δ -115.14. **HRMS (ESI)** Calcd. for $C_{14}H_{11}FN_2$ ($[M+H]^+$): 227.0985, found: 227.0978.

Characterization data for isolated products

3-Acetylpyridine (2a)[10]

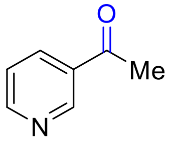

Reacted 3-ethylpyridine (111.5 mg, 1.04 mmol) according to the general procedure in a vortex shaker in BuOAc at 90 °C. Chromatography: 30→60% EtOAc/hexanes, 1% triethylamine.

Yield: 84% (105.7 mg). Yellow oil.

^{1}H NMR (500 MHz, $CDCl_3$) δ 9.19-9.14 (m, 1H), 8.78 (d, J = 4.7 Hz, 1H), 8.23 (d, J = 8.5 Hz, 1H), 7.42 (dd, J = 7.9, 4.8 Hz, 1H), 2.64 (s, 3H). **^{13}C NMR** (126 MHz, $CDCl_3$) δ 196.86, 153.71, 150.12, 135.58, 132.41, 123.76, 26.88.

2-Benzoylpyridine (2b)[10]

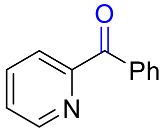

Reacted 2-benzylpyridine (174.4 mg, 1.03 mmol) according to the general procedure in a vortex shaker in BuOAc at 90 °C. Chromatography: 20→40% EtOAc/hexanes, 1% triethylamine.
Yield: 93% (175.2 mg). Pale yellow oil.
^{1}**H NMR** (500 MHz, $CDCl_3$) δ 8.73 (d, J = 4.1 Hz, 1H), 8.10-8.03 (m, 3H), 7.90 (td, J = 7.8, 1.7 Hz, 1H), 7.60 (t, J = 7.4 Hz, 1H), 7.52–7.44 (m, 3H). ^{13}C NMR (126 MHz, $CDCl_3$) δ 194.03, 155.24, 148.69, 137.19, 136.40, 133.06, 131.11, 128.30, 126.29, 124.76.

3-Benzoylpyridine (2c)[10]

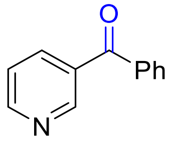

Reacted 3-benzylpyridine (166.4 mg, 0.98 mmol) according to the general procedure in a vial in BuOAc at 90 °C. Chromatography: 5→10% EtOAc/DCM.
Yield: 92% (165.5 mg). Yellow oil.
^{1}**H NMR** (500 MHz, $CDCl_3$) δ 8.99 (d, 1.5 Hz, 1H), 8.81 (dd, J = 4.8, 1.6 Hz, 1H), 8.12 (dt, J = 7.9, 2.0 Hz, 1H), 7.82 (dd, J = 8.3, 1.2 Hz, 2H), 7.64 (tt, J = 7.1, 1.3 Hz, 1H), 7.52 (d, J = 15.4 Hz, 2H), 7.46 (ddd, J = 7.9, 4.9, 0.8 Hz, 1H). ^{13}**C NMR** (126 MHz, $CDCl_3$) δ 195.03, 152.98, 151.09, 137.33, 136.85, 133.32, 130.17, 128.76, 123.51.

4-Benzoylpyridine (2d)[10]

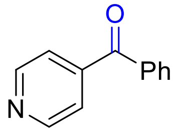

Reacted 4-benzylpyridine (174.2 mg, 1.03 mmol) according to the general procedure in a vortex shaker in BuOAc at 90 °C. Chromatography: 10→50% EtOAc/hexanes, 1% triethylamine.
Yield: 89% (168.8 mg). Pale yellow powder.
^{1}**H NMR** (500 MHz, $CDCl_3$) δ 8.80 (dd, J = 4.3, 1.6 Hz, 2H), 7.81 (dd, J = 8.3, 1.2 Hz, 2H), 7.63 (t, J = 7.4 Hz, 1H), 7.59 – 7.54 (m, 2H), 7.50 (t, J = 7.8 Hz, 2H). ^{13}**C NMR** (126 MHz, $CDCl_3$) δ 195.26, 150.46, 144.46, 135.99, 133.64, 130.24, 128.76, 122.97.

4-Acetylpyridine (2e)[10]

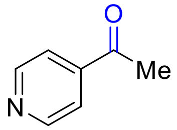

Reacted 4-ethylpyridine (107.8 mg, 1.01 mmol) according to the general procedure in a vortex shaker in BuOAc at 100 °C. Chromatography: 20→80% EtOAc/hexanes, 1% triethylamine.
Yield: 56% (68.8 mg). Yellow oil.

^{1}H NMR (500 MHz, $CDCl_3$) δ 8.81 (d, *J* = 5.6 Hz, 2H), 7.72 (d, *J* = 6.0 Hz, 2H), 2.63 (s, 3H). **^{13}C NMR** (126 MHz, $CDCl_3$) δ 197.45, 151.09, 142.85, 121.36, 26.79.

1-Pyridin-3-yl-butan-1-one (2f)[11]

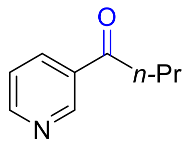

Reacted 3-butylpyridine (141.0 mg, 1.04 mmol) according to the general procedure in a vortex shaker in BuOAc at 100 °C. Chromatography: 10→40% EtOAc/hexanes, 1% triethylamine.
Yield: 60% (92.7 mg). Pale yellow oil.
^{1}H NMR (500 MHz, $CDCl_3$) δ 9.17 (d, *J* = 1.3 Hz, 1H), 8.77 (dd, *J* = 4.8, 1.5 Hz, 1H), 8.23 (dt, *J* = 7.9, 1.9 Hz, 1H), 7.42 (dd, *J* = 7.9, 4.9 Hz, 1H), 2.97 (t, *J* = 7.2 Hz, 2H), 1.79 (h, *J* = 7.4 Hz, 2H), 1.02 (t, *J* = 7.4 Hz, 3H). **^{13}C NMR** (126 MHz, $CDCl_3$) δ 199.25, 153.46, 149.75, 135.49, 132.39, 123.79, 77.41, 40.93, 17.59, 13.94.

2-Benzoyl-1*H*-benzimidazole (2g)[12]

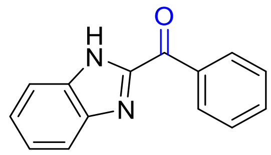

Reacted 2-benzylbenzimidazole (208.3 mg, 1.00 mmol) according to the general procedure in a vial in BuOAc at 100 °C. Chromatography: 100% DCM.
Yield: 94% (210.0 mg). White powder.
^{1}H NMR ^{1}H NMR (500 MHz, $CDCl_3$) δ 10.68 (s, 1H), 8.71 (d, *J* = 7.9 Hz, 2H), 7.98 (d, *J* = 8.2 Hz, 1H), 7.67 (t, *J* = 7.4 Hz, 1H), 7.62 – 7.54 (m, 3H), 7.47 – 7.43 (m, 1H), 7.41 – 7.35 (m, 1H). **^{13}C NMR** (126 MHz, $CDCl_3$) δ 184.16, 147.88, 144.12, 135.51, 134.05, 133.29, 131.46, 128.68, 126.61, 123.92, 122.45, 112.11.

1*H*-Benzimidazol-2-yl(4-methoxyphenyl)methanone (2h)[13]

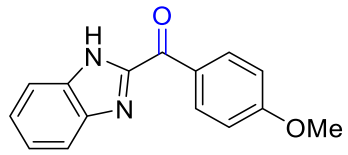

Reacted 2-(4-methoxybenzyl)benzimidazole (238.3 mg, 1.00 mmol) according to the general procedure in a vortex shaker in BuOAc at 90 °C. Chromatography: 0→10% EtOAc/DCM.
Yield: 92% (231.2 mg). Pale yellow powder.
^{1}H NMR (500 MHz, $CDCl_3$) δ 10.59 (s, 1H), 8.81 (d, *J* = 9.0 Hz, 2H), 7.97 (d, *J* = 8.2 Hz, 1H), 7.59 (d, *J* = 8.1 Hz, 1H), 7.43 (t, *J* = 7.1 Hz, 1H), 7.37 (t, *J* = 7.7 Hz, 1H), 7.09 – 7.01 (m, 2H),

3.92 (s, 3H). **^{13}C NMR** (126 MHz, $CDCl_3$) δ 182.18, 164.55, 148.34, 144.13, 134.10, 134.01, 133.13, 128.46, 126.30, 123.82, 122.29, 114.13, 113.97, 112.04, 55.72.

1-(4-(Pyridin-3-yl)phenyl)ethanone (2i)[14]

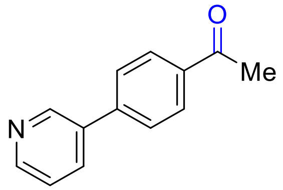

Reacted 3-(4-ethylphenyl)pyridine (183.0 mg, 1.00 mmol) according to the general procedure in a vortex shaker in BuOAc at 90 °C. Chromatography: 20→70% EtOAc/Hexanes, 1% triethylamine.
Yield: 87% (171.7 mg). White solid.
^{1}H NMR (500 MHz, $CDCl_3$) δ 8.90 (d, *J* = 1.9 Hz, 1H), 8.66 (dd, *J* = 4.8, 1.5 Hz, 1H), 8.09 (d, *J* = 8.4 Hz, 2H), 7.93 (dt, *J* = 7.9, 2.2 Hz, 1H), 7.70 (d, *J* = 8.4 Hz, 2H), 7.42 (dd, *J* = 8.6, 4.8 Hz, 1H), 2.67 (s, 3H). **^{13}C NMR** (126 MHz, $CDCl_3$) δ 197.64, 149.47, 148.47, 142.47, 136.63, 135.53, 134.58, 129.24, 127.40, 123.79, 26.82.

4′-(Imidazol-1-yl)acetophenone (2j)[15]

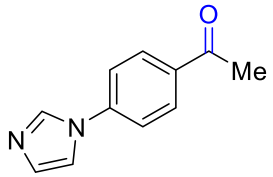

Reacted 1-(4-ethylphenyl)-1*H*-imidazole (172.5 mg, 1.00 mmol) in BuOAc in a vortex shaker at 100 °C. Chromatography: 2% triethylamine in EtOAc.
Yield: 66% (122.4 mg). Off-white solid.
^{1}H NMR (500 MHz, $CDCl_3$) δ 8.08 (d, *J* = 8.7 Hz, 2H), 7.95 (s, 1H), 7.49 (d, *J* = 8.7 Hz, 2H), 7.35 (s, 1H), 7.23 (s, 1H), 2.63 (s, 3H). ^{13}C NMR (126 MHz, $CDCl_3$) δ 196.65, 140.83, 135.88, 135.50, 131.24, 130.44, 120.83, 117.84, 26.74.

4-Acetamidoacetophenone (2k)[16]

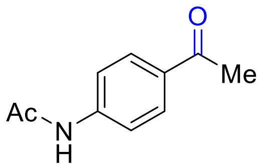

Reacted 4-ethylacetanilide (163.2 mg, 1.00 mmol) according to the general procedure in a vortex shaker in BuOAc at 90 °C. The reaction mixture was also charged with 3-chlorobenzoic acid (15.7 mg, 0.10 mmol) as a catalytic additive.[17] Chromatography: 20→80% EtOAc/Hexanes.
Yield: 74% (130.2 mg). Pale yellow solid.

^{1}H NMR (500 MHz, DMSO-d_6) δ 10.27 (s, 1H), 7.91 (d, J = 8.8 Hz, 2H), 7.70 (d, J = 8.7 Hz, 2H), 2.51 (s, 3H), 2.08 (s, 3H). **^{13}C NMR** (126 MHz, DMSO-d_6) δ 196.45, 168.91, 143.63, 131.48, 129.46, 118.11, 26.40, 24.18.

2-Hexanoylthiophene (2m)[18]

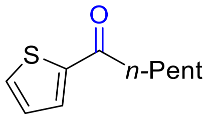

Reacted 2-hexylthiophene (171.0 mg, 1.02 mmol) according to the general procedure in a vortex shaker in 7:3 BuOAc:pyr at 90 °C. Chromatography: 0→5% EtOAc/Hexanes.
Yield: 65% (120.7 mg). Yellow oil.
^{1}H NMR (500 MHz, $CDCl_3$) δ 7.70 (dd, J = 3.8, 1.0 Hz, 1H), 7.61 (dd, J = 4.9, 1.0 Hz, 1H), 7.12 (dd, J = 4.9, 3.8 Hz, 1H), 2.89 (t, J = 7.4 Hz, 2H), 1.75 (p, J = 7.4 Hz, 2H), 1.40-1.30 (m, 4H), 0.91 (m, 3H). **^{13}C NMR** (126 MHz, $CDCl_3$) δ 193.70, 144.67, 133.43, 131.77, 128.15, 39.55, 31.65, 24.63, 22.62, 14.08.

3-Hexanoylthiophene (2n)[19]

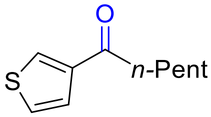

Reacted 3-hexylthiophene (169.2 mg, 1.005 mmol) according to the general procedure in a vortex shaker in 7:3 BuOAc:pyr at 90 °C. Chromatography: 0→5% EtOAc/Hexanes.
Yield: 48% (87.5 mg). White solid.
^{1}H NMR (500 MHz, $CDCl_3$) δ 8.04 (dd, J = 2.9, 1.2 Hz, 1H), 7.55 (dd, J = 5.1, 1.1 Hz, 1H), 7.31 (dd, J = 5.1, 2.9 Hz, 1H), 2.86 (t, J = 7.4 Hz, 2H), 1.73 (p, J = 7.4 Hz, 2H), 1.41–1.28 (m, 4H), 0.91 (t, J = 7.0 Hz, 3H). **^{13}C NMR** (126 MHz, $CDCl_3$) δ 195.14, 142.62, 131.78, 127.15, 126.37, 40.07, 31.69, 24.26, 22.67, 14.10.

2-benzoylbenzothiazole (2o)[12]

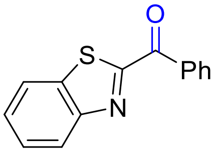

Reacted 2-benzylbenzothiazole (226.1 mg, 1.003 mmol) according to the general procedure in a vortex shaker in 7:3 BuOAc:pyr at 90 °C. Chromatography: 0→5% EtOAc/Hexanes. The solid that was obtained from chromatography was triturated with hexanes.
Yield: 64% (154.1 mg).
^{1}H NMR (500 MHz, $CDCl_3$) δ 8.56 (d, J = 7.2 Hz, 2H), 8.25 (d, J = 7.6 Hz, 1H), 8.03 (d, J = 7.6 Hz, 1H), 7.68 (t, J = 7.4 Hz, 1H), 7.63 – 7.50 (m, 4H). **^{13}C NMR** (126 MHz, $CDCl_3$) δ 185.54, 167.26, 154.04, 137.16, 135.12, 134.06, 131.43, 128.66, 127.78, 127.08, 125.89, 122.33.

1-methyl-2-benzoylbenzimidazole (2p)[20]

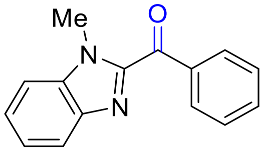

Reacted 1-methyl-2-(benzyl)benzimidazole (222.3 mg, 1.00 mmol) according to the general procedure in a vortex shaker in 7:3 BuOAc:pyr at 90 °C. Chromatography: 5→25% EtOAc/Hexanes.
Yield: 80% (190 mg).
^{1}H NMR (500 MHz, $CDCl_3$) δ 8.35 (d, *J* = 7.9 Hz, 2H), 7.93 (d, *J* = 8.2 Hz, 1H), 7.64 (t, *J* = 7.4 Hz, 1H), 7.57–7.44 (m, 4H), 7.39 (t, *J* = 7.5 Hz, 1H), 4.17 (s, 3H). **^{13}C NMR** (126 MHz, $CDCl_3$) δ 186.60, 146.80, 142.01, 137.00, 136.67, 133.65, 131.34, 128.51, 125.85, 123.76, 122.23, 110.56, 32.39.

2-acetylpyridine (2q)[10]

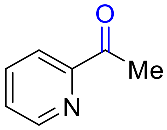

This reaction was performed according to the general procedure for the electrochemical aerobic oxidation using 2-ethylpyridine. The crude product was purified by column chromatography on silica gel (Hexanes/EtOAc gradient 90/10 to 70/30) to give 82% of the product (87.9 mg, 0.82 mmol) as a light yellow liquid.
^{1}H NMR (400 MHz, DMSO-d_6): δ 8.67–8.65 (m, 1H); 8.00 (d, 1H, *J* = 7.6 Hz); 7.80 (dt, 1H, *J* = 1.6 Hz, *J* = 7.6 Hz); 7.46-7.43 (m, 1H); 2.71 (s, 3H) **^{13}C NMR** (CDCl3, 125 MHz) δ 200.1, 153.5, 148.9, 136.8, 127.0, 121.6, 25.7.

2-acetylbenzimidazole (2r)[21]

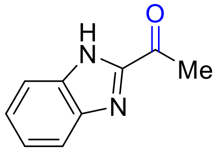

This reaction was performed according to the general procedure for the electrochemical aerobic oxidation using 2-ethylbenzimidazole. The crude product was purified by column chromatography on silica gel (Hexanes/EtOAc gradient 90/10 to 70/30) to give 70% of the product (112.1 mg, 0.70 mmol) as an off-white solid.
^{1}H NMR (400 MHz, DMSO-d_6): δ 13.29 (br s, 1H), 7.82 (d, *J* = 8.1 Hz, 1H), 7.55 (d, *J* = 8.1, 1H), 7.38 (dt, *J* = 1.0, *J* = 7.1, 1H), 7.31 (dt, *J* = 1.0, *J* = 7.1, 1H), 2.70 (s, 3H). **^{13}C NMR** (125 MHz, DMSO-d_6): δ 182.6, 139.9, 134.4, 126.1, 117.6, 115.3, 112.2, 104.3.

Multi-gram synthesis of 2-(4-fluorobenzoyl)benzimidazole (2s, Scheme 5)

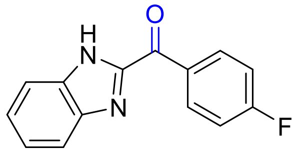

A 100 mL Parr reactor was charged with $Co(OAc)_2 \cdot 4H_2O$ (22.0 mg, 0.088 mmol, 0.01 equiv), NHPI (288.4 mg, 1.77 mmol, 0.2 equiv), 2-(4-fluorobenzyl)benzimidazole (2.002 g, 8.85 mmol), EtOAc (8.8 mL), and a Teflon stir bar. The reactor was sealed and pressurized with 9% O_2 in N_2 (500 psi), and the reaction mixture was stirred in an oil bath set to 90 °C for 12 h. After the reaction mixture cooled to rt, the reactor was depressurized. The reaction mixture was diluted with additional EtOAc (30 mL) and was treated with 25% NaOH (8 mL) for 6 min in a separatory funnel. The aqueous layer was diluted with additional water (8 mL), and the extraction was continued for 1 min. The organic layer was separated, and the aqueous layer was treated with EtOAc (30 mL). The combined organic fractions were next filtered through a silica gel plug, which was then washed with EtOAc (20 mL). The volatiles were removed under reduced vacuum to yield **2r** (2.017 g, 8.40 mmol, 95% yield) as a pale yellow powder.
^{1}H NMR (500 MHz, $CDCl_3$) δ 10.65 (s, 1H), 8.82 (dd, J = 8.7, 5.6 Hz, 2H), 7.97 (d, J = 8.2 Hz, 1H), 7.59 (d, J = 8.1 Hz, 1H), 7.45 (t, J = 7.5 Hz, 1H), 7.39 (t, J = 7.6 Hz, 1H), 7.24 (apparent triplet, J = 9.1 Hz, 2H). ^{13}C NMR (126 MHz, $CDCl_3$) δ 182.33, 166.57 (d, J = 256.7 Hz), 147.73, 144.07 , 134.39 (d, J = 9.5 Hz), 133.25, 131.84 (d, J = 2.8 Hz), 126.72, 124.02, 122.40, 115.90 (d, J = 21.8 Hz), 112.11. **^{19}F NMR** (377 MHz, $CDCl_3$) δ -103.39. **HRMS (ESI)** Calcd. for $C_{14}H_{11}FN_2$ ($[M+H]^+$): 241.0772, found: 241.0767.

E-factor analysis

Nitrile addition route (Scheme 6B)

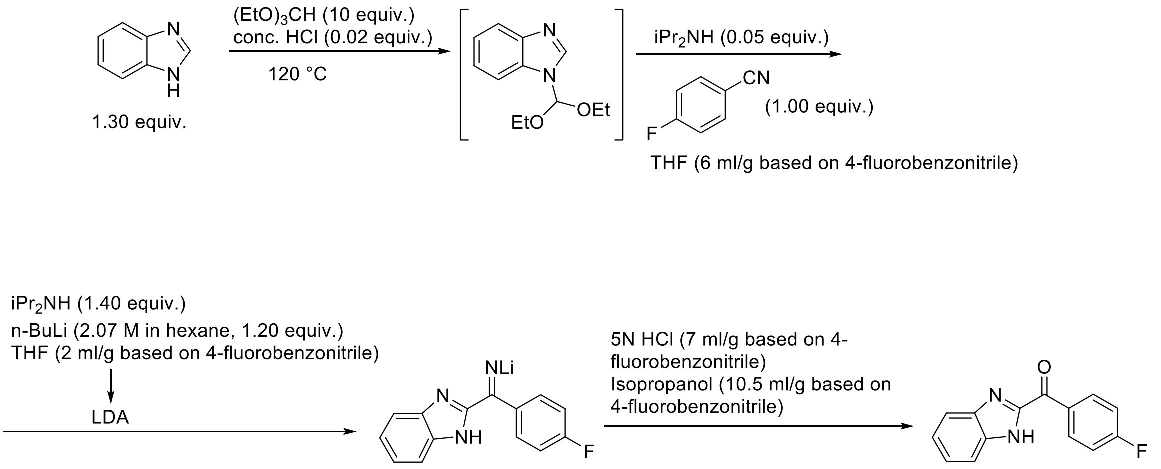

**Compound**	**MW**	**Equiv**	**Yield**	**mmol**	**Mass (g)**	**Volume (ml)**	**Density**	**Molarity**
Benzimidazole	118.14	1.30		536.70	63.41			
Triethylorthoformate	148.20	10.00		4128.48	611.84	686.69	0.891	
Conc. Hydrochloric acid	36.46	0.02		8.26	0.84	0.71	1.18	11.60
Diisopropylamine	101.19	0.05		20.64	2.09	2.94	0.71	
4-Fluorobenzonitrile	121.11	1.00		412.85	50.00			
THF	72.11				269.70	300.00	0.899	
Diisopropylamine	101.19	1.40		577.99	58.49	82.38	0.71	
n-BuLi	35.98	1.20		495.42	164.91	239.00	0.69	2.07
THF	72.11				89.90	100.00	0.899	
5 N HCl	36.46				371.00	350.00	1.06	
Isopropanol	60.10				412.65	525.00	0.786	
Product **2t**	240.23		80.66	333.01	80.00			

Total E-Factor	25.19
Total E-Factor without quench/isolation	15.39

Co/NHPI oxidation route (Scheme 5c)

For the purpose of estimating the E-factor for the two-step condensation/oxidation sequence, we assumed a quantitative yield for the condensation step (for justification, see ref. **Error! Bookmark not defined.** in which a near-quantitative yield was obtained for preparation of the parent 2-benzylbenzimidazole substrate via a neat condensation reaction). Solvent use for isolation was not included in the analysis.

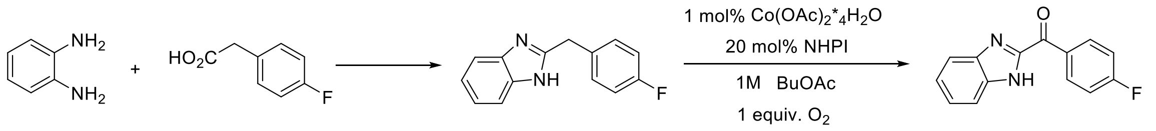

Compound	MW	Equiv	Yield	mmol	Mass (g)	Volume (ml)	Density	Molarity
Benzene-1,2-diamine	108.14	1.00		9.247	1.000			
2-(4-Fluorophenyl)acetic acid	154.14	1.00		9.247	1.425			
2-(4-Fluorobenzyl)-1H-benzo[d]imidazole	226.25		100	9.247	2.092			

E-Factor step 1 | 0.16

Compound	MW	Equiv	Yield	mmol	Mass (g)	Volume (ml)	Density	Molarity
2-(4-fluorobenzyl)-1H-benzo[d]imidazole	226.25	1.00		9.247	2.092			1 M in BuOAc
Oxygen	32.00	1.00		9.247	0.296			
Cobalt(II)acetate tetrahydrate	241.02	0.01		0.092	0.022			
NHPI	163.13	0.20		1.85	0.30			
Ethyl acetate	88.11				8.34	9.247	0.902	
**Compound 2t**	240.23		95.00	8.78	2.11			

E-Factor step 1 | 4.23

E-Factor combined for steps 1&2 | 4.39

References

1 O. Algul, A. Kaessler, Y. Apcin, A. Yilmaz and J. Jose, *Molecules*, 2008, **13**, 736.
2 F. Luo, J. Yang, Z. Li, H. Xiang and X. Zhou, *Eur. J. Org. Chem.*, 2015, **2015**, 2463.
3 X. Wang, Q. Liu, H. Yan, Z. Liu, M. Yao, Q. Zhang, S. Gong and W. He, *Chem. Commun.*, 2015, **51**, 7497.
4 T. Iwasawa, D. Ajami and J. Rebek, Jr., *Org. Lett.*, 2006, **8**, 2925.
5 M. S. Viciu, R. F. Germaneau, O. Navarro-Fernandez, E. D. Stevens and S. P. Nolan, *Organometallics*, 2002, **21**, 5470.
6 K. Swapna, A. Vijay Kumar, V. Prakash Reddy and K. Rama Rao, *J. Org. Chem.*, 2009, **74**, 7514.
7 E. Sperotto, J. G. de Vries, G. P. M. van Klink and G. van Koten, *Tetrahedron Lett.*, 2007, **48**, 7366.
8 H. Matsushita, S.-H. Lee, M. Joung, B. Clapham and K. D. Janda, *Tetrahedron Lett.*, 2004, **45**, 313.
9 B. Pilarski, *Liebigs Ann. Chem.*, 1983, 1078.
10 Spectroscopic data obtained for the isolated product matched the data contained in online spectroscopic databases (either Sigma Aldrich or the Spectral Database for Organic Compounds, SDBS).
11 A. Takemiya and J. F. Hartwig, *J. Am. Chem. Soc.*, 2006, **128**, 14800.
12 A. Dos Santos, L. El Kaim and L. Grimaud, *Org. Biomol. Chem.*, 2013, **11**, 3282.
13 H. Yang, F. N. Murigi, Z. Wang, J. Li, H. Jin and Z. Tu, *Bioorg. Med. Chem. Lett.*, 2015, **25**, 919.
14 C. L. Cioffi, W. T. Spencer, J. J. Richards and R. J. Herr, *J. Org. Chem.*, 2004, **69**, 2210.
15 V. K. Marrapu, V. Chaturvedi, S. Singh, S. Singh, S. Sinha and K. Bhandari, *Eur. J. Med. Chem.*, 2011, **46**, 4302.
16 C.-H. Park and R. S. Givens, *J. Am. Chem. Soc.*, 1997, **119**, 2453.
17 T. Iwahama, Y. Yoshino, T. Keitoku, S. Sakaguchi and Y. Ishii, *J. Org. Chem.*, 2000, **65**, 6502.
18 J. Ruan, O. Saidi, J. A. Iggo and J. Xiao, *J. Am. Chem. Soc.*, 2008, **130**, 10510.
19 J. Liu, X. Zhou, H. Rao, F. Xiao, C.-J. Li and G.-J. Deng, *Chem. - Eur. J.*, 2011, **17**, 7996.
20 A. Miyashita, Y. Suzuki, I. Nagasaki, C. Ishiguro, K.-I. Iwamoto and T. Higashino, *Chem. Pharm. Bull.*, 1997, **45**, 1254.
21 Reddy, V. M.; Reddy, K. R. *Chin. Chem. Lett.* **2010**, *21*, 1145.

^{1}H NMR, ^{13}C NMR, and ^{19}F NMR spectra

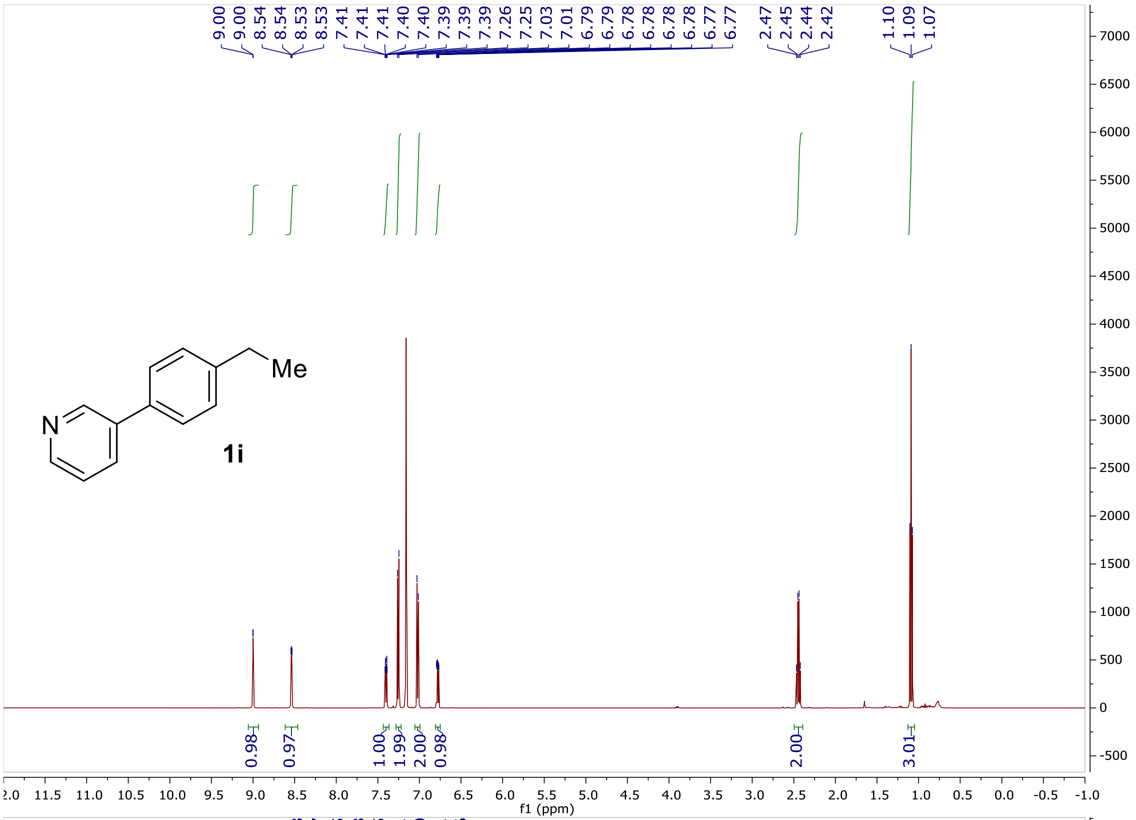

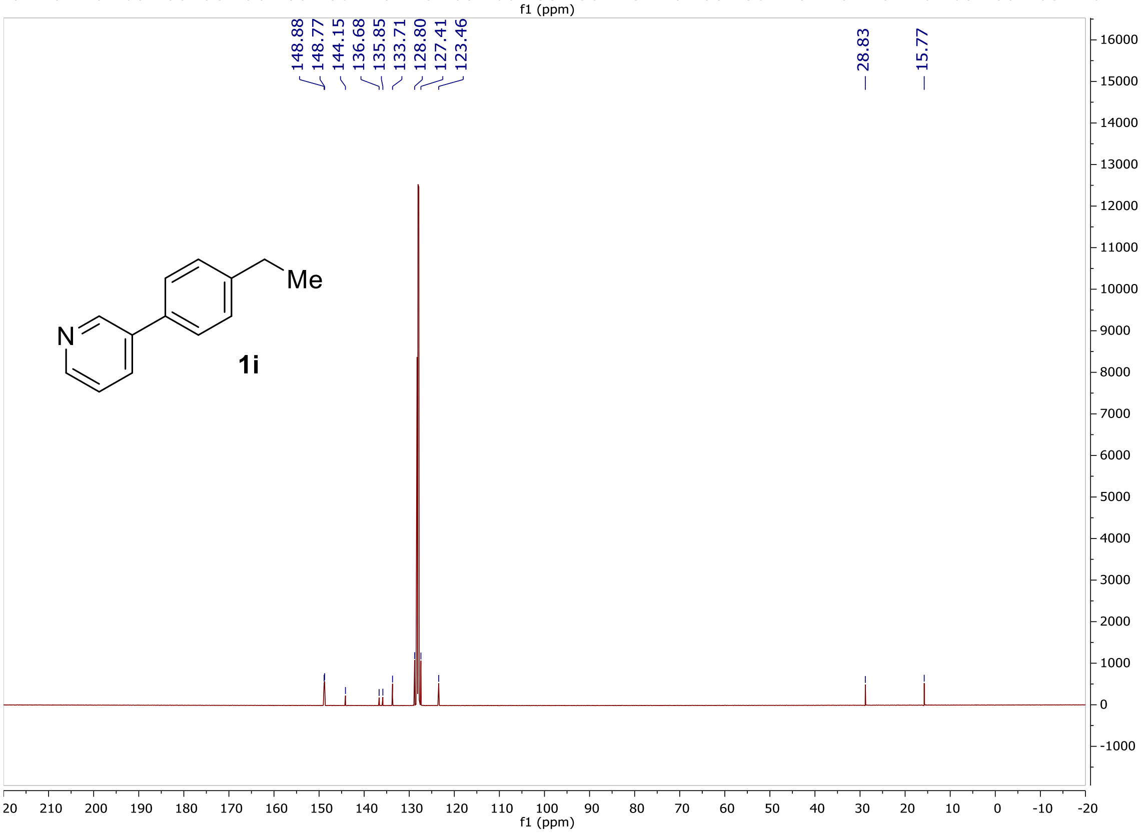

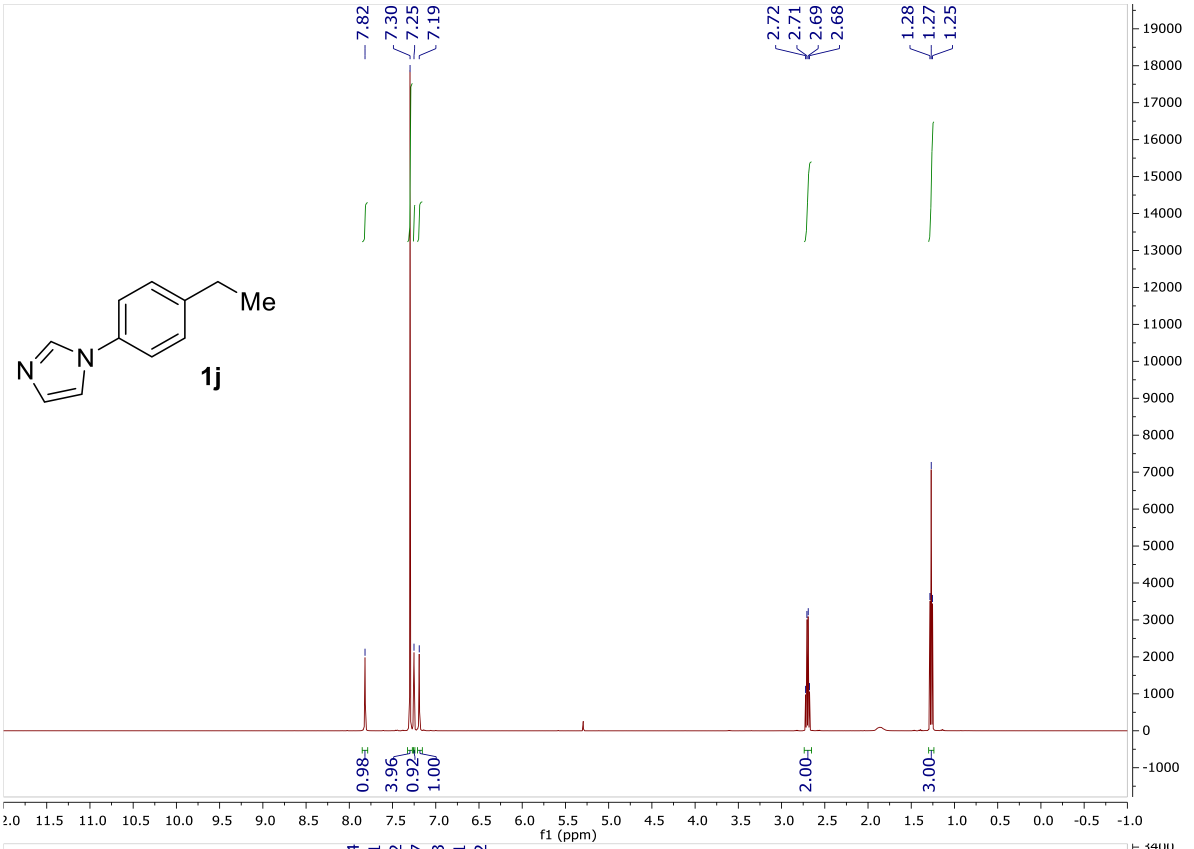

7.82
7.30
7.25
7.19
2.72
2.71
2.69
2.68
1.28
1.27
1.25
Me
1j
0.98
3.96
0.92
1.00
2.00
3.00
f1 (ppm)

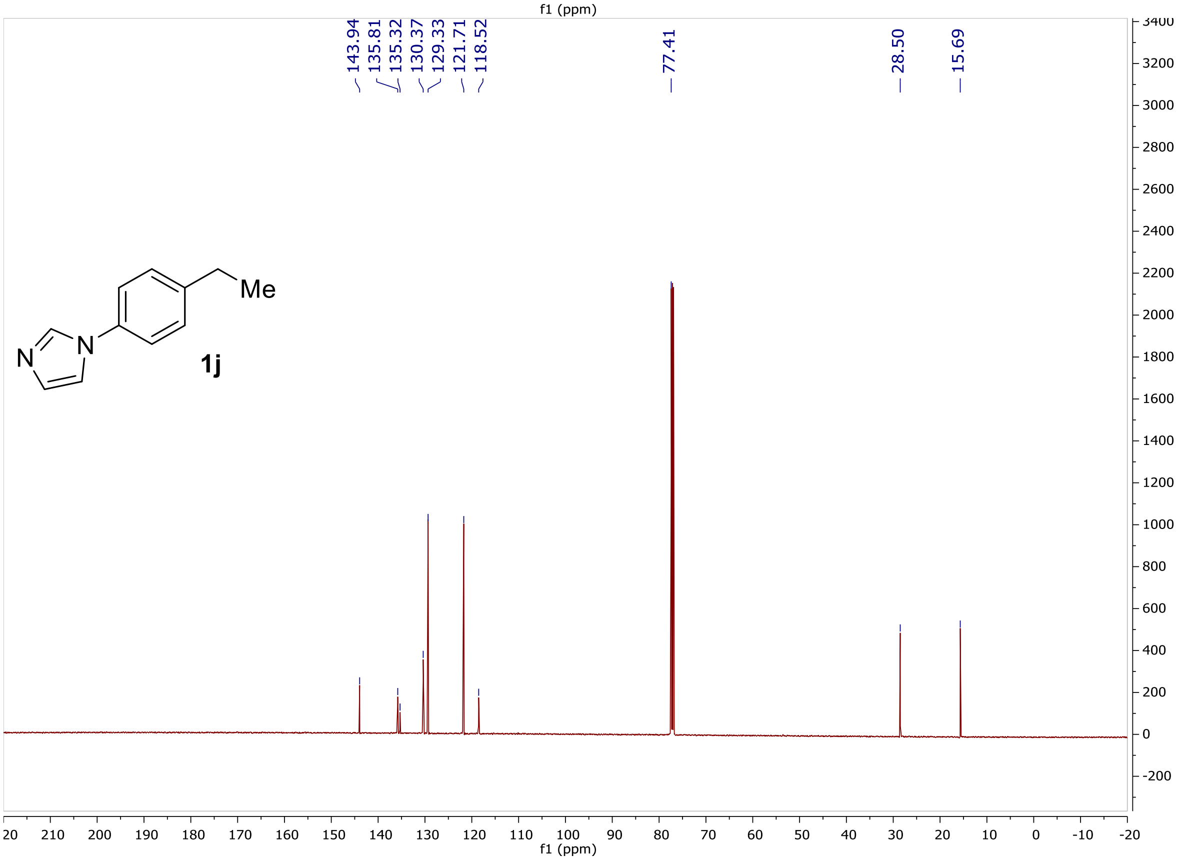

143.94
135.81
135.32
130.37
129.33
121.71
118.52
77.41
28.50
15.69
Me
1j
f1 (ppm)

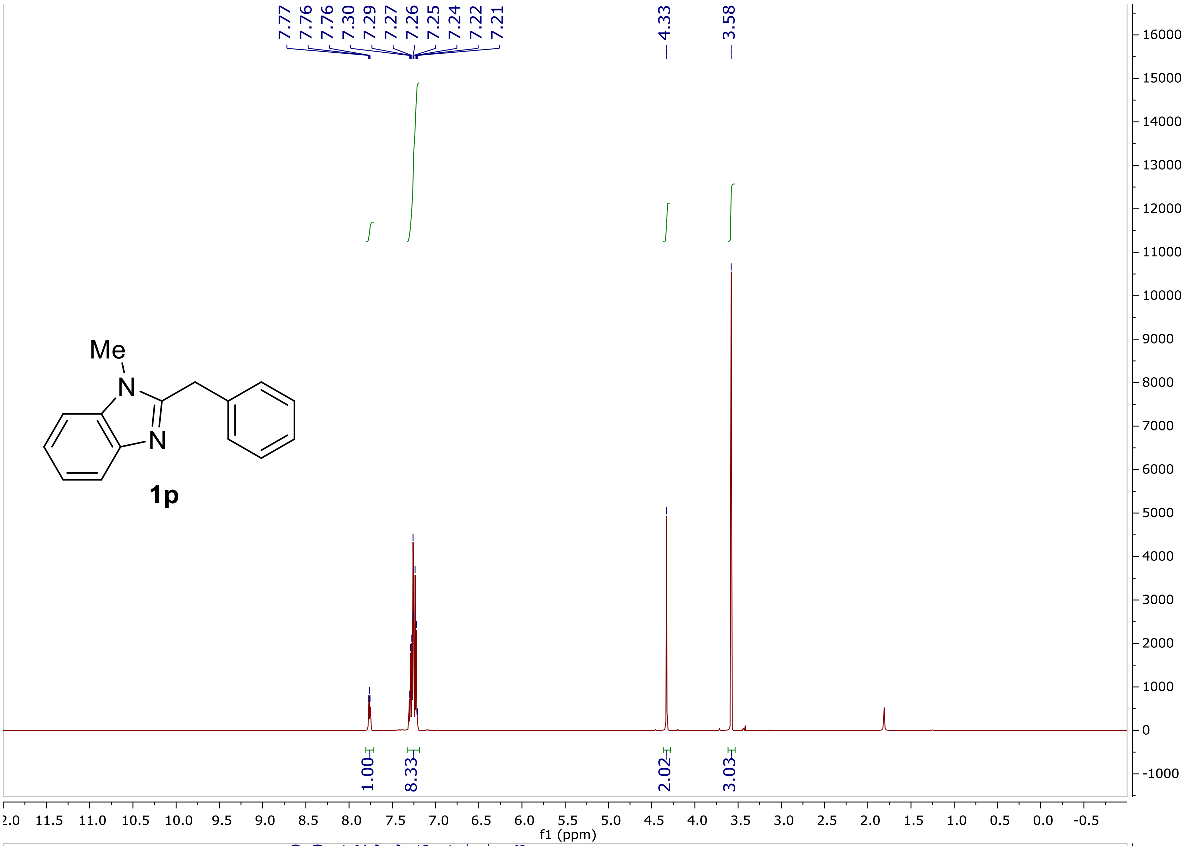

7.77
7.76
7.76
7.30
7.29
7.27
7.26
7.25
7.24
7.22
7.21
4.33
3.58
Me
N
N
1p
1.00
8.33
2.02
3.03
f1 (ppm)

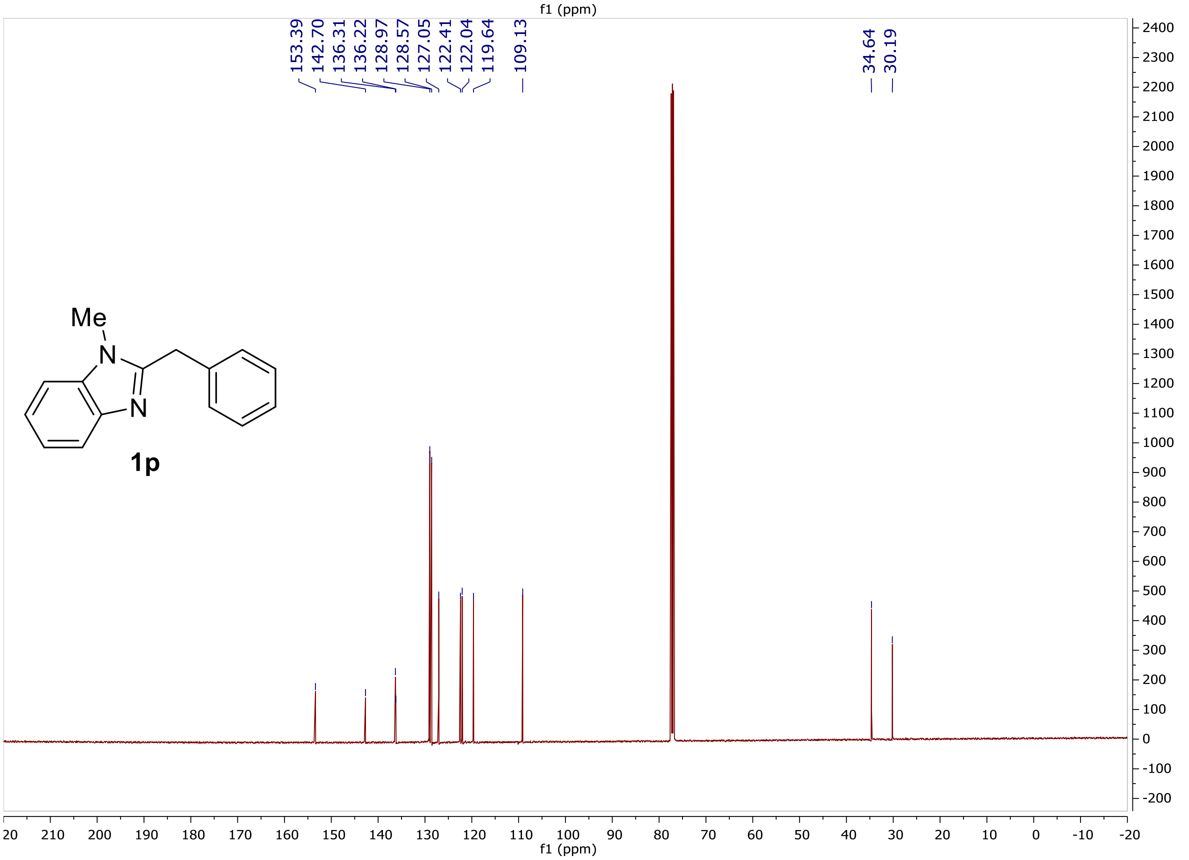

153.39
142.70
136.31
136.22
128.97
128.57
127.05
122.41
122.04
119.64
109.13
34.64
30.19
Me
N
N
1p
f1 (ppm)

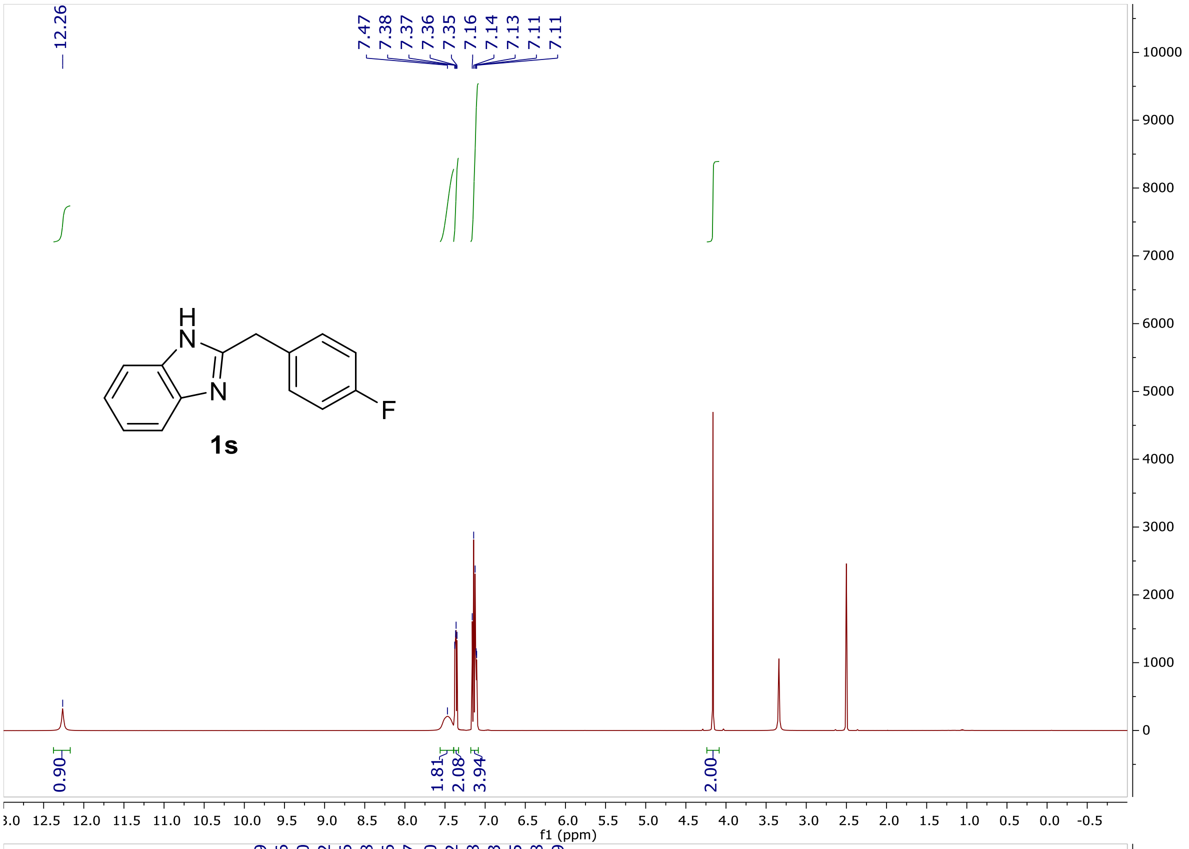

12.26
7.47
7.38
7.37
7.36
7.35
7.16
7.14
7.13
7.11
7.11
1s
F
0.90
1.81
2.08
3.94
2.00
f1 (ppm)

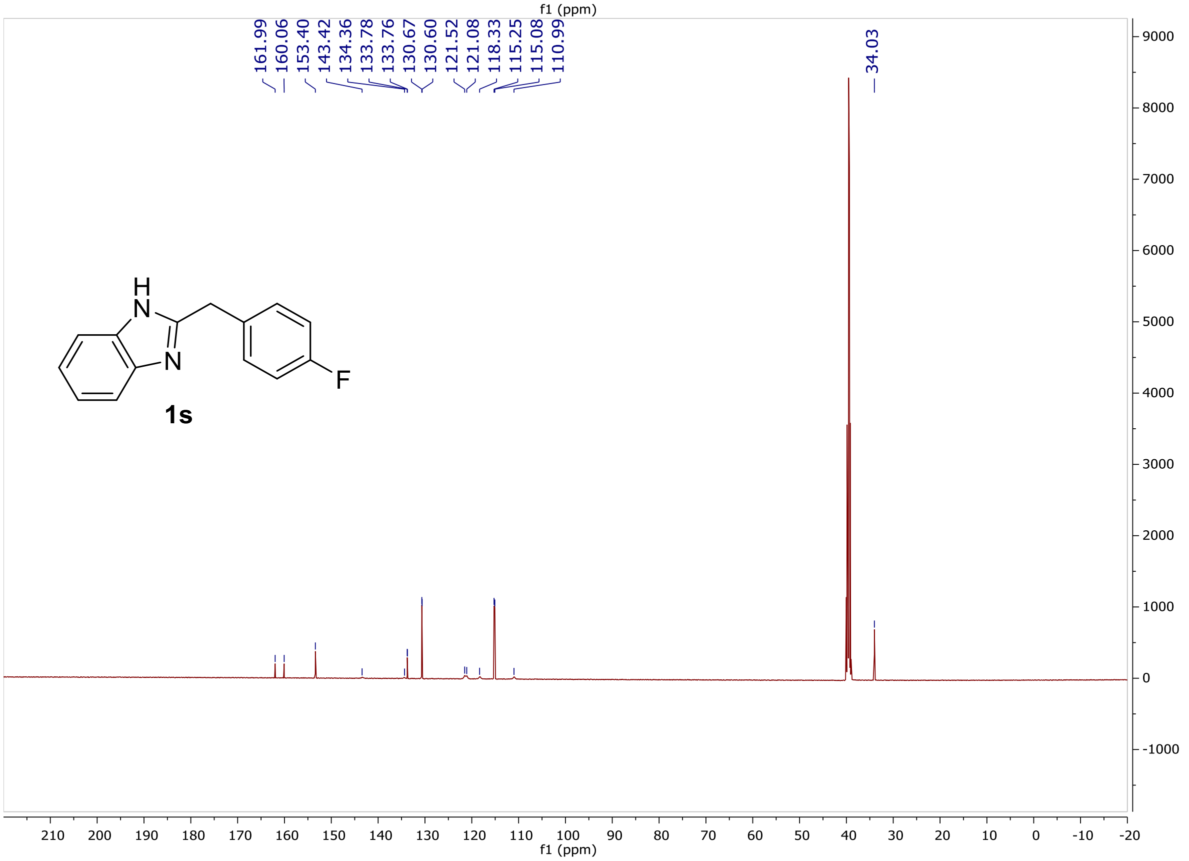

161.99
160.06
153.40
143.42
134.36
133.78
133.76
130.67
130.60
121.52
121.08
118.33
115.25
115.08
110.99
34.03
1s
F
f1 (ppm)

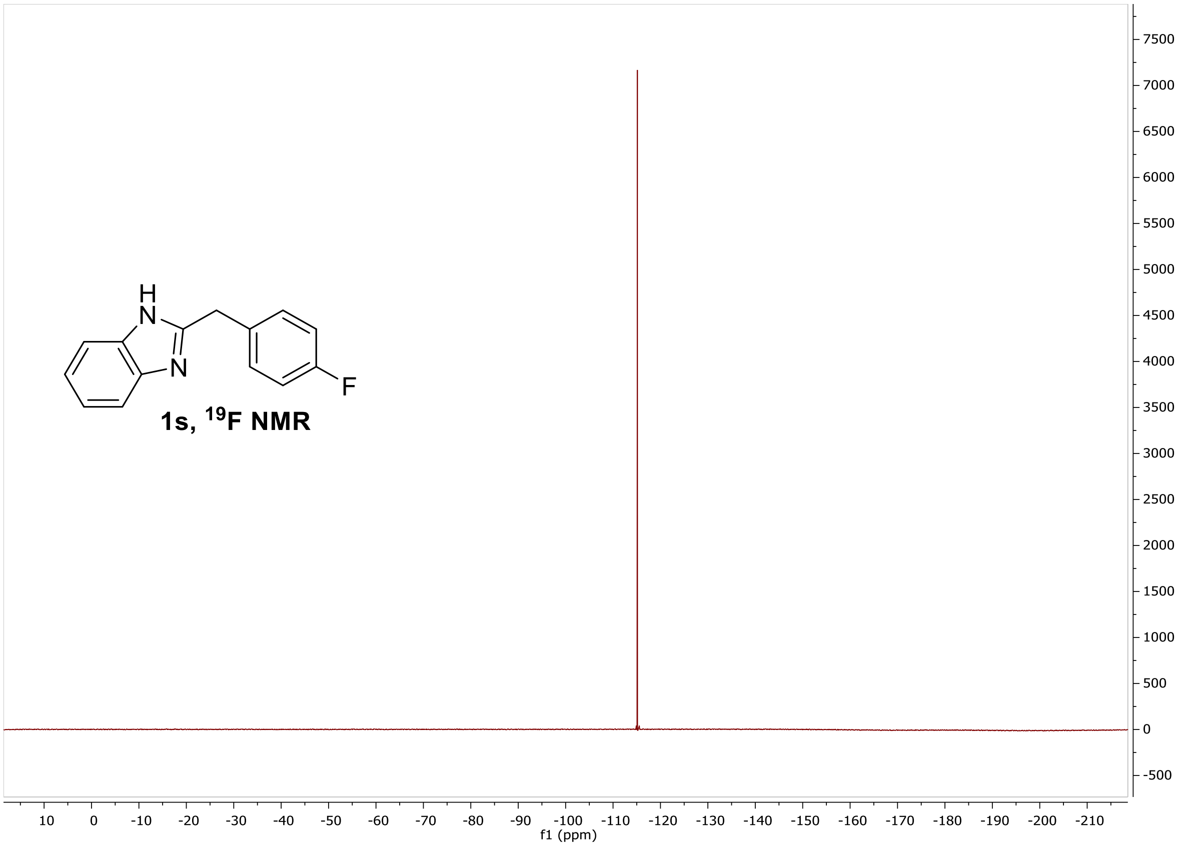
1s, ^{19}F NMR
f1 (ppm)

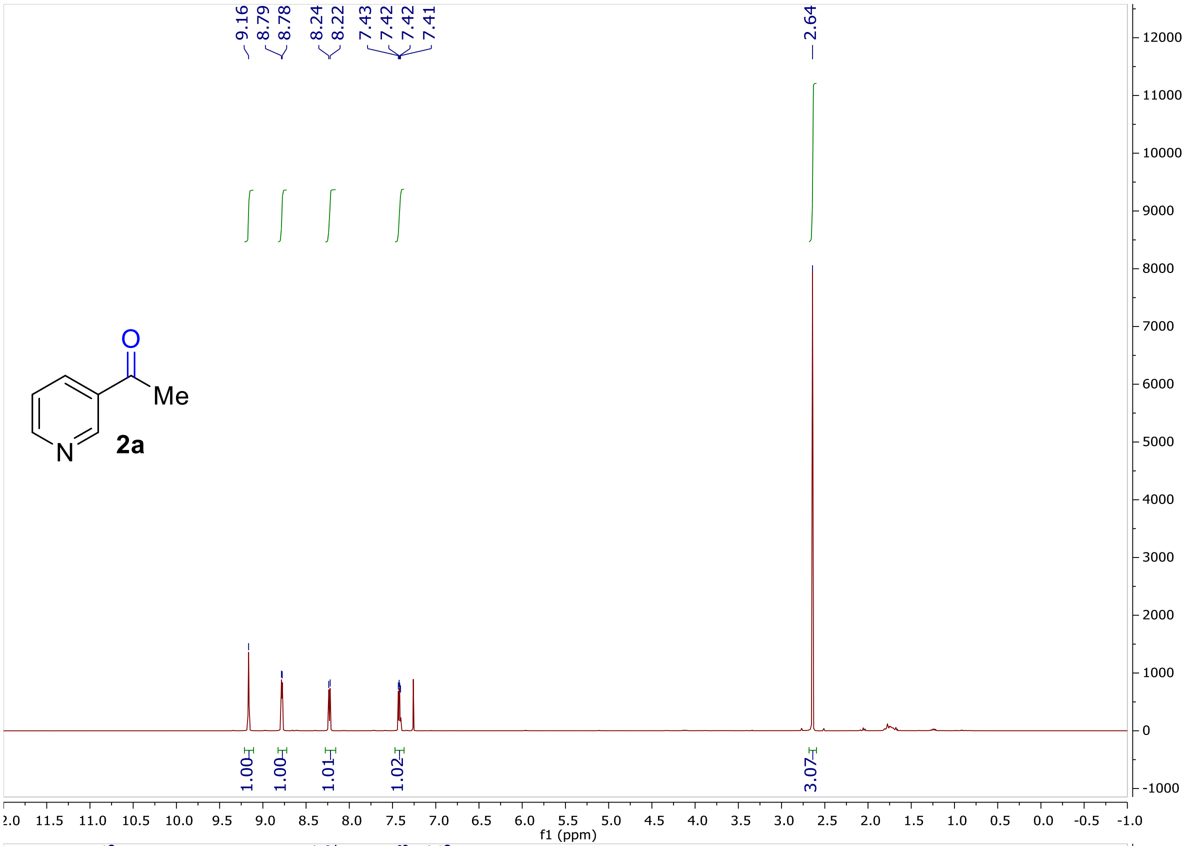
9.16
8.79
8.78
8.24
8.22
7.43
7.42
7.42
7.41
2.64
1.00
1.00
1.01
1.02
3.07
O
Me
N
2a
f1 (ppm)

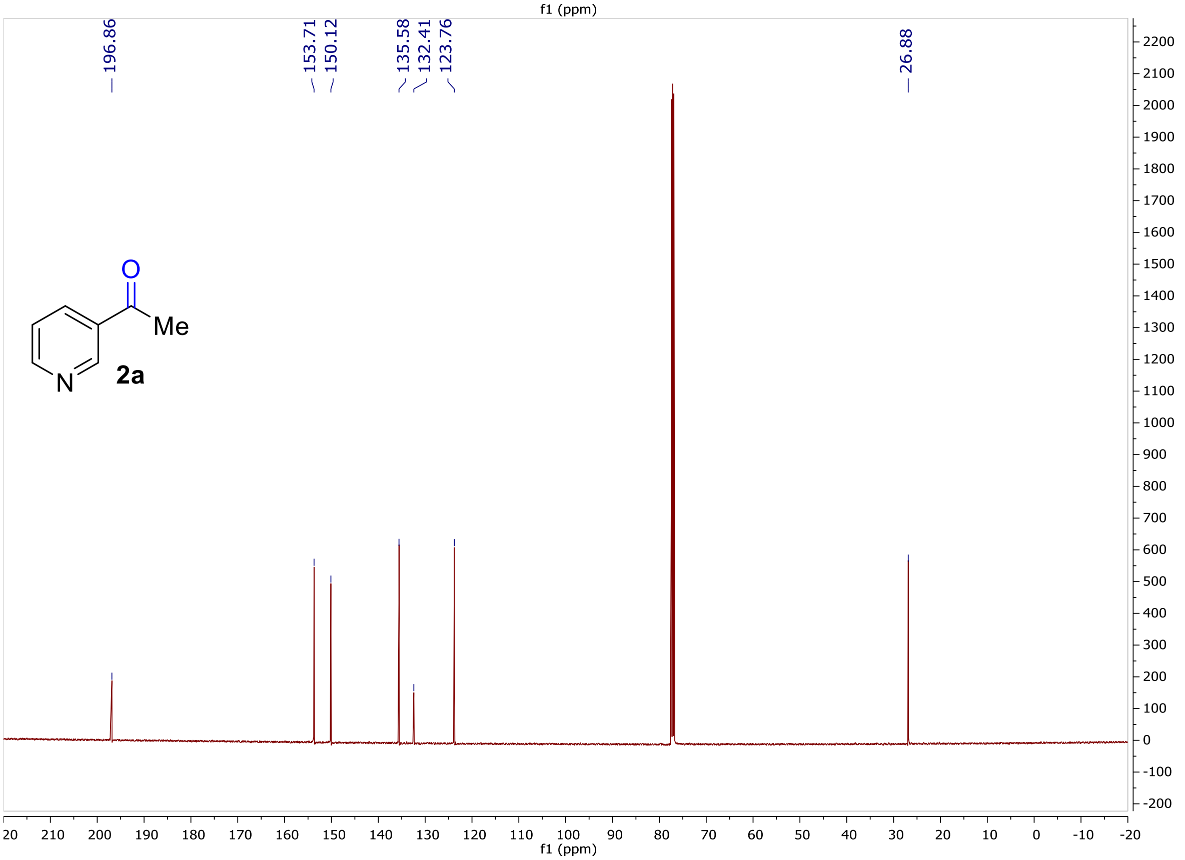
196.86
153.71
150.12
135.58
132.41
123.76
26.88
O
Me
N
2a
f1 (ppm)

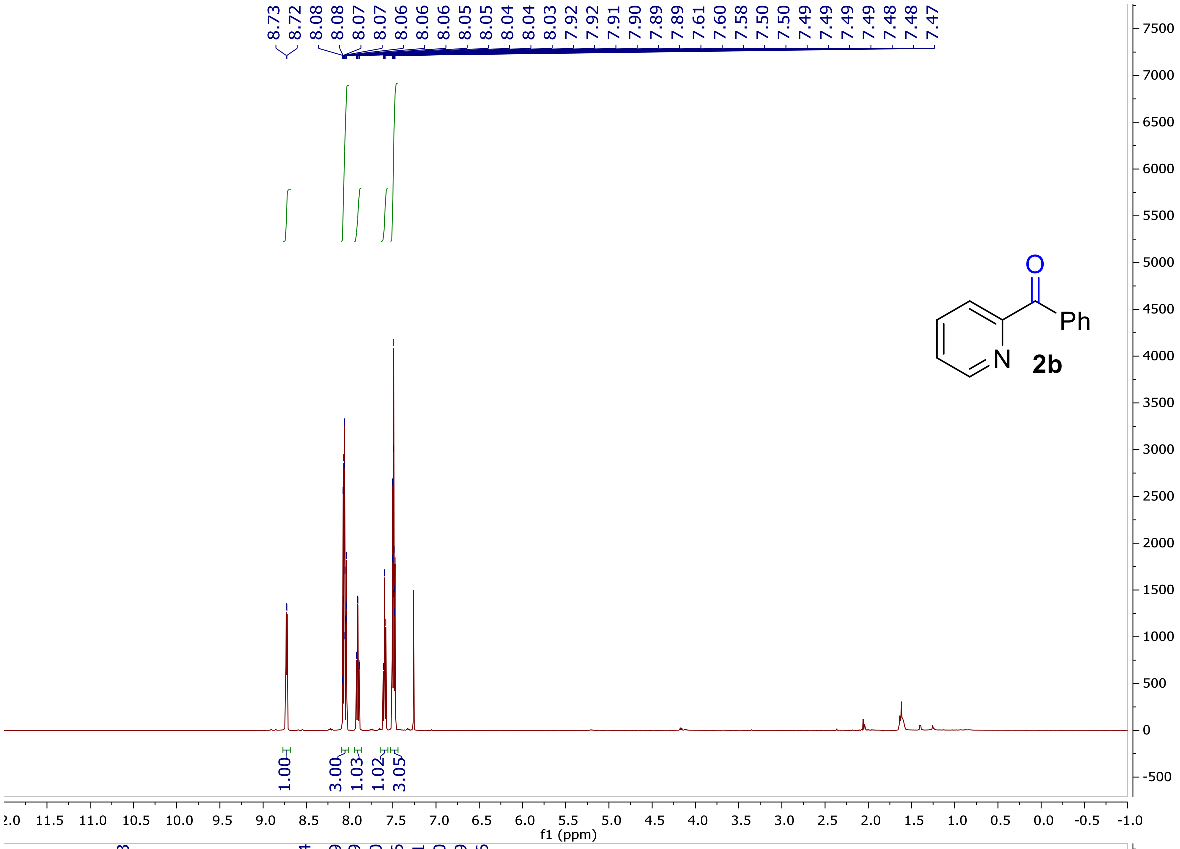
8.73
8.72
8.08
8.08
8.07
8.07
8.06
8.06
8.06
8.05
8.05
8.04
8.04
8.03
7.92
7.92
7.91
7.90
7.89
7.89
7.61
7.60
7.58
7.50
7.50
7.49
7.49
7.49
7.49
7.48
7.47
1.00
3.00
1.03
1.02
3.05
Ph
N
2b
f1 (ppm)

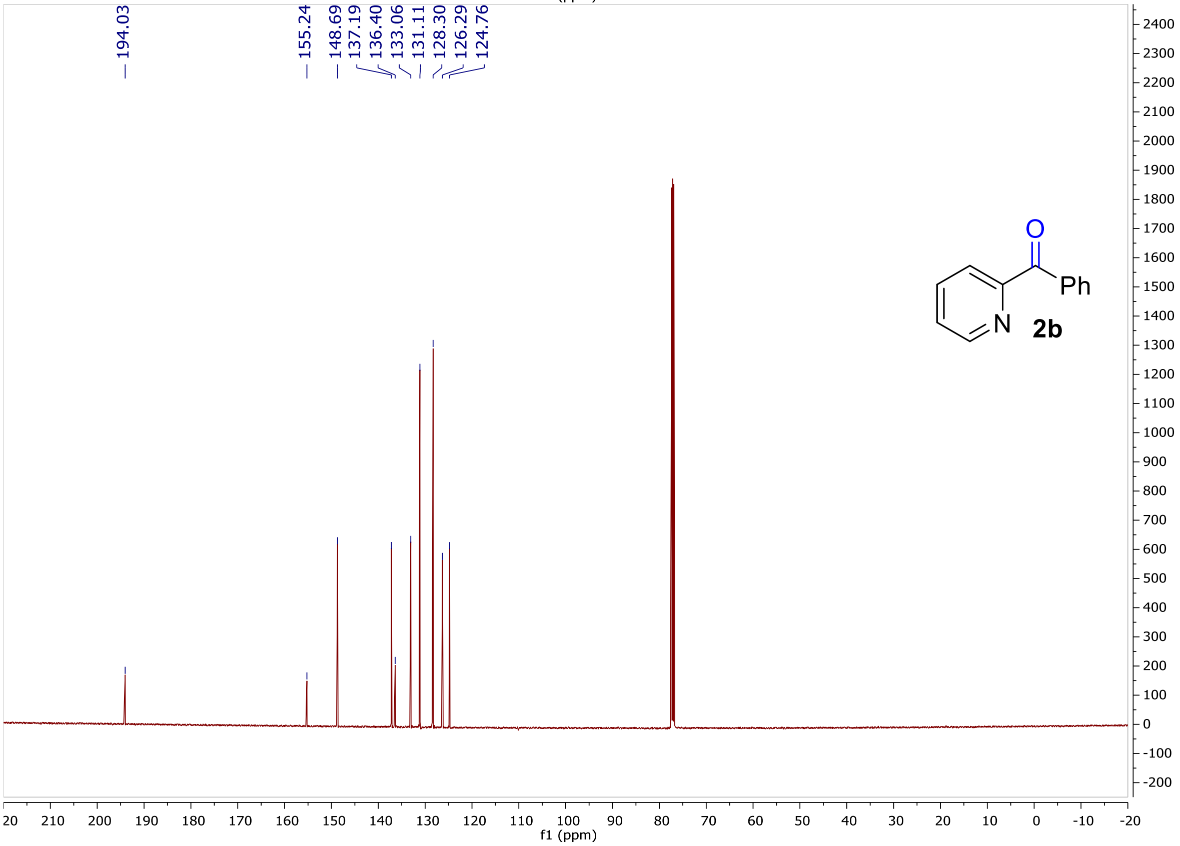
194.03
155.24
148.69
137.19
136.40
133.06
131.11
128.30
126.29
124.76
Ph
N
2b
f1 (ppm)

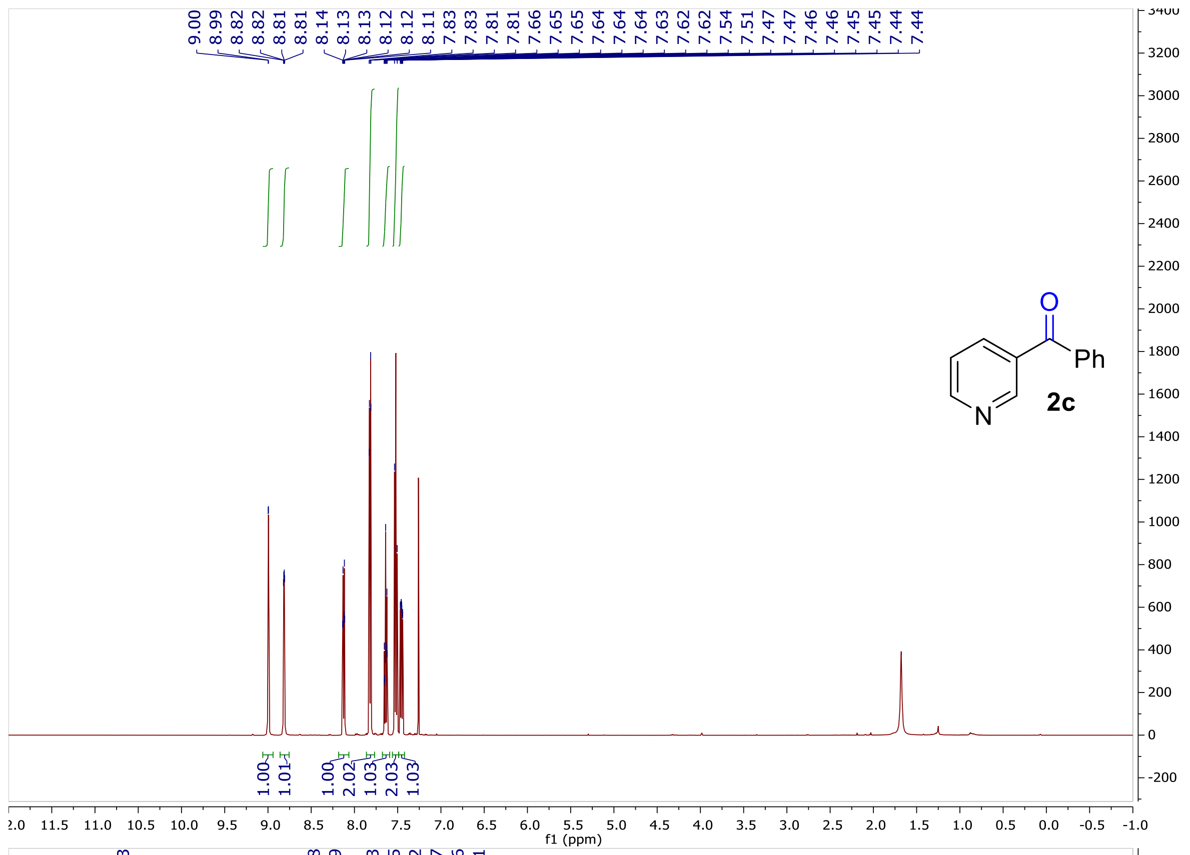

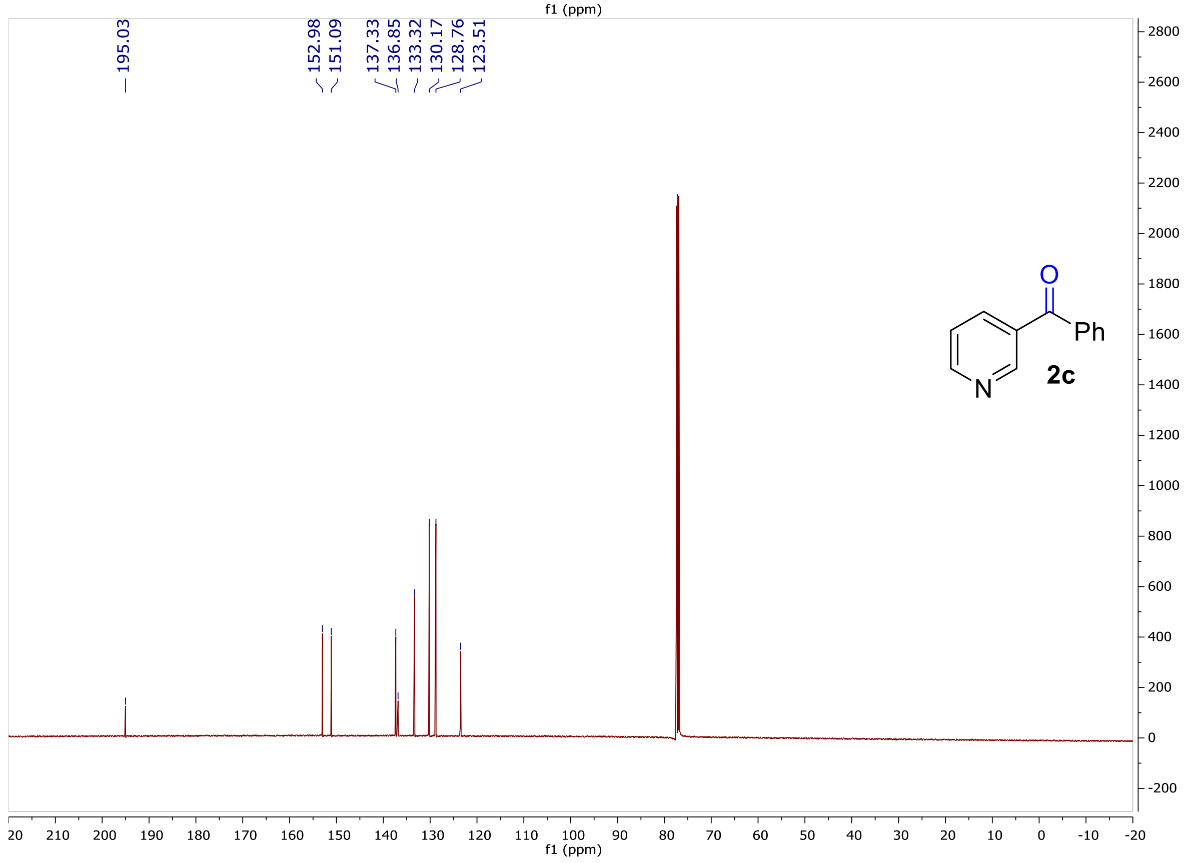

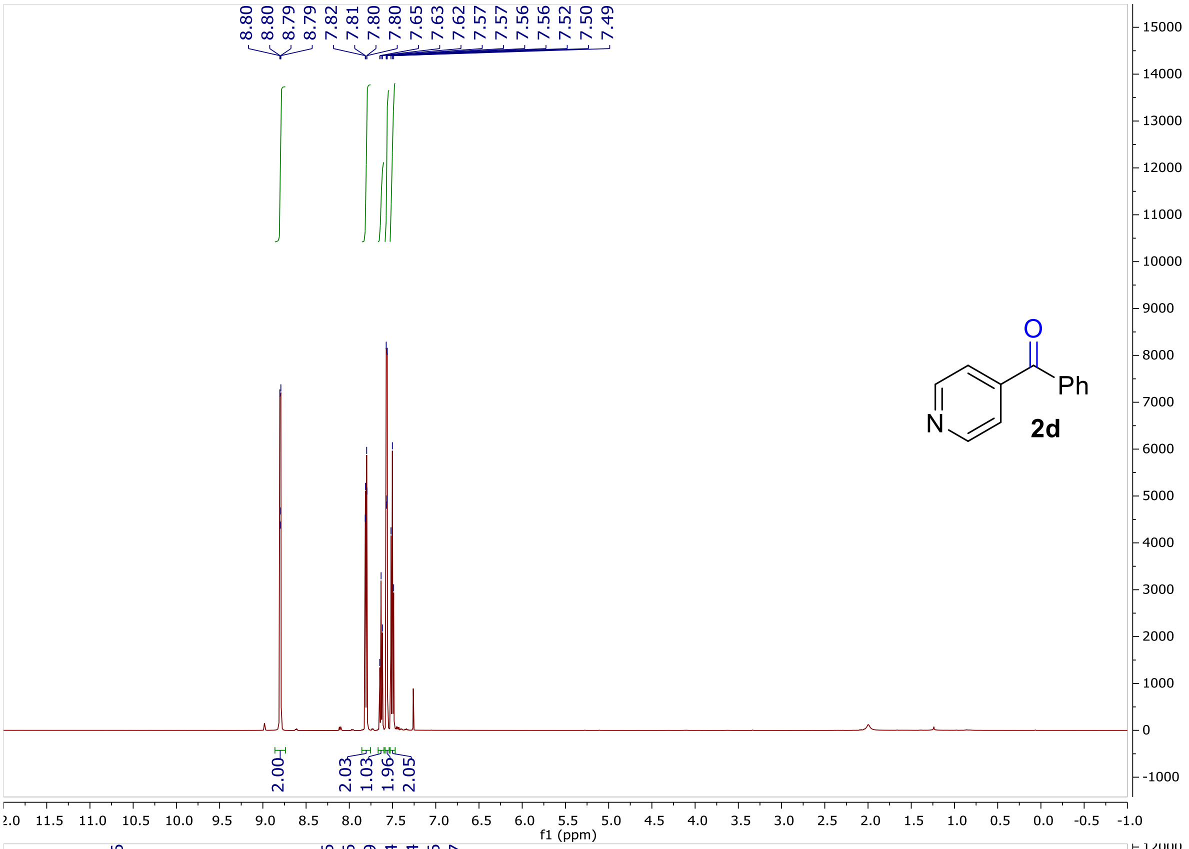
8.80
8.80
8.79
8.79
7.82
7.81
7.80
7.80
7.65
7.63
7.62
7.57
7.57
7.56
7.56
7.52
7.50
7.49
2.00
2.03
1.03
1.96
2.05
f1 (ppm)
Ph
N
2d

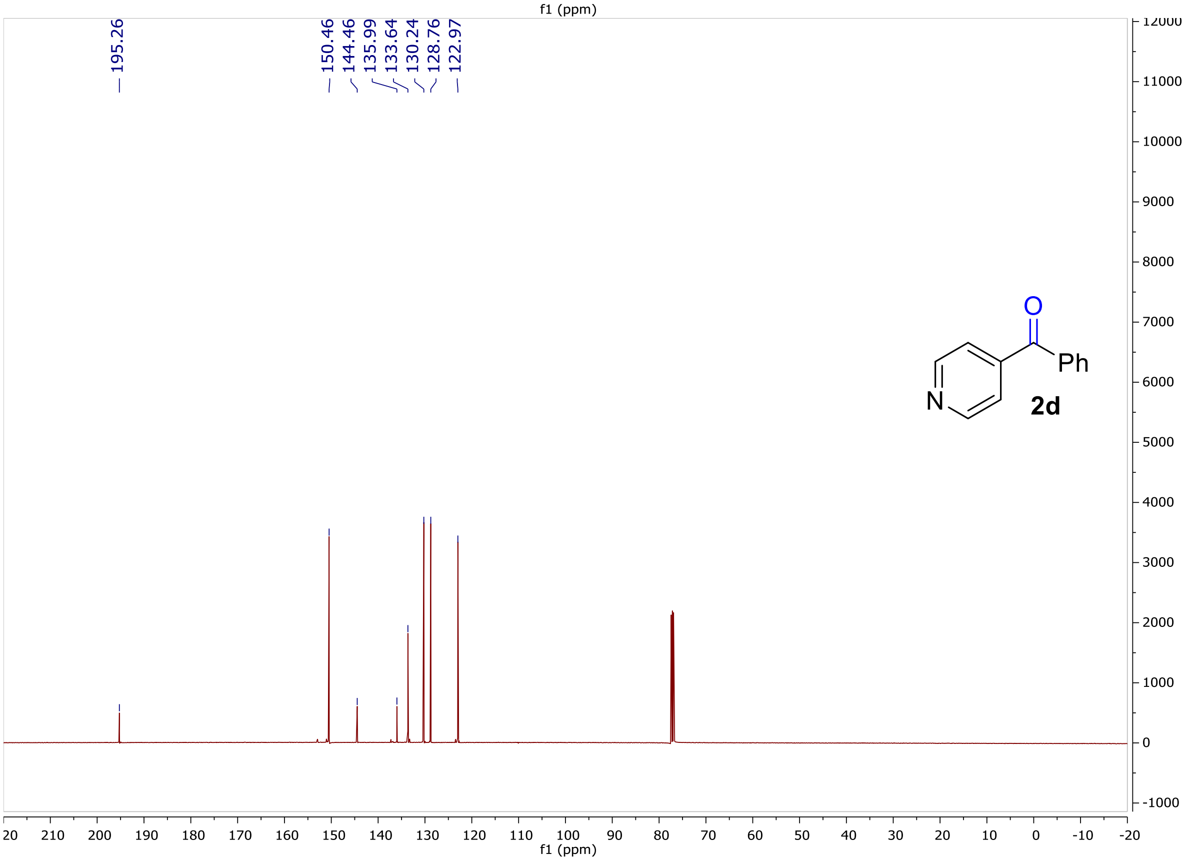
195.26
150.46
144.46
135.99
133.64
130.24
128.76
122.97
f1 (ppm)
Ph
N
2d

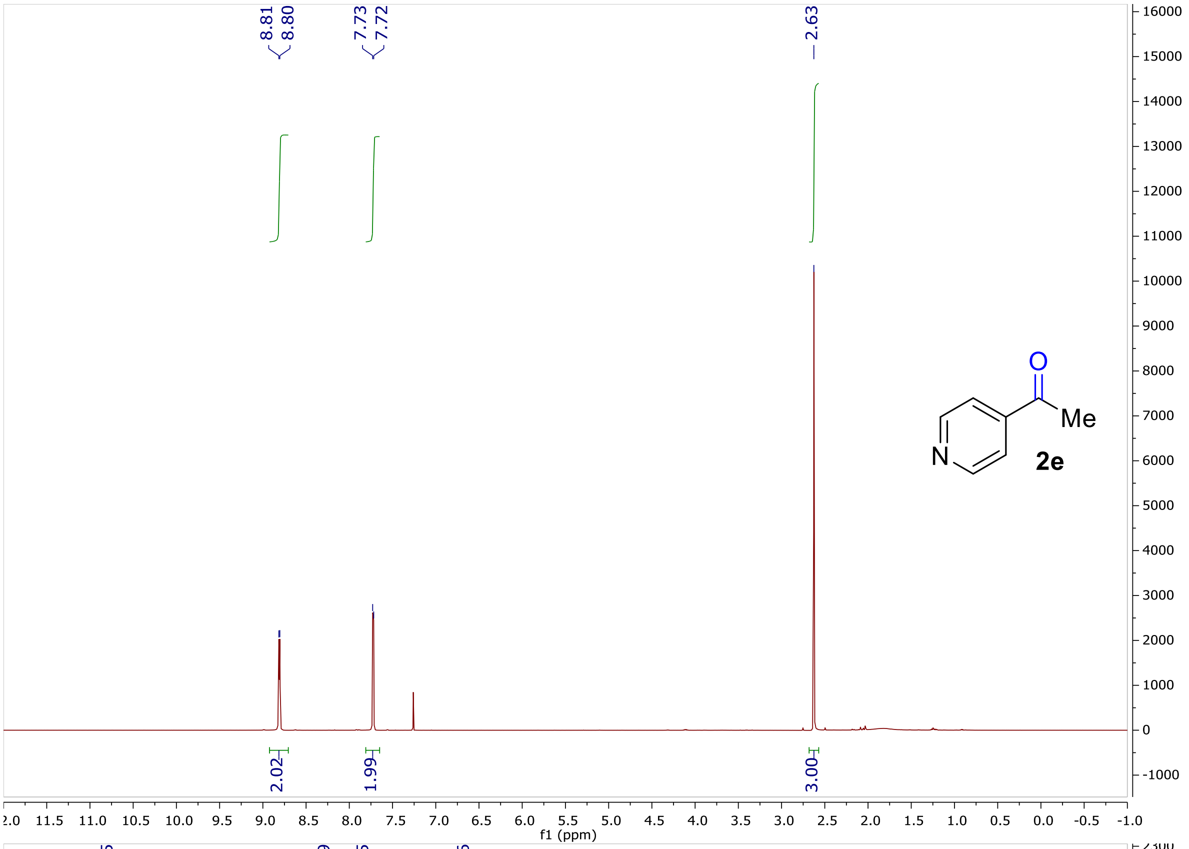
8.81
8.80
7.73
7.72
2.63
2.02
1.99
3.00
f1 (ppm)
O
Me
N
2e

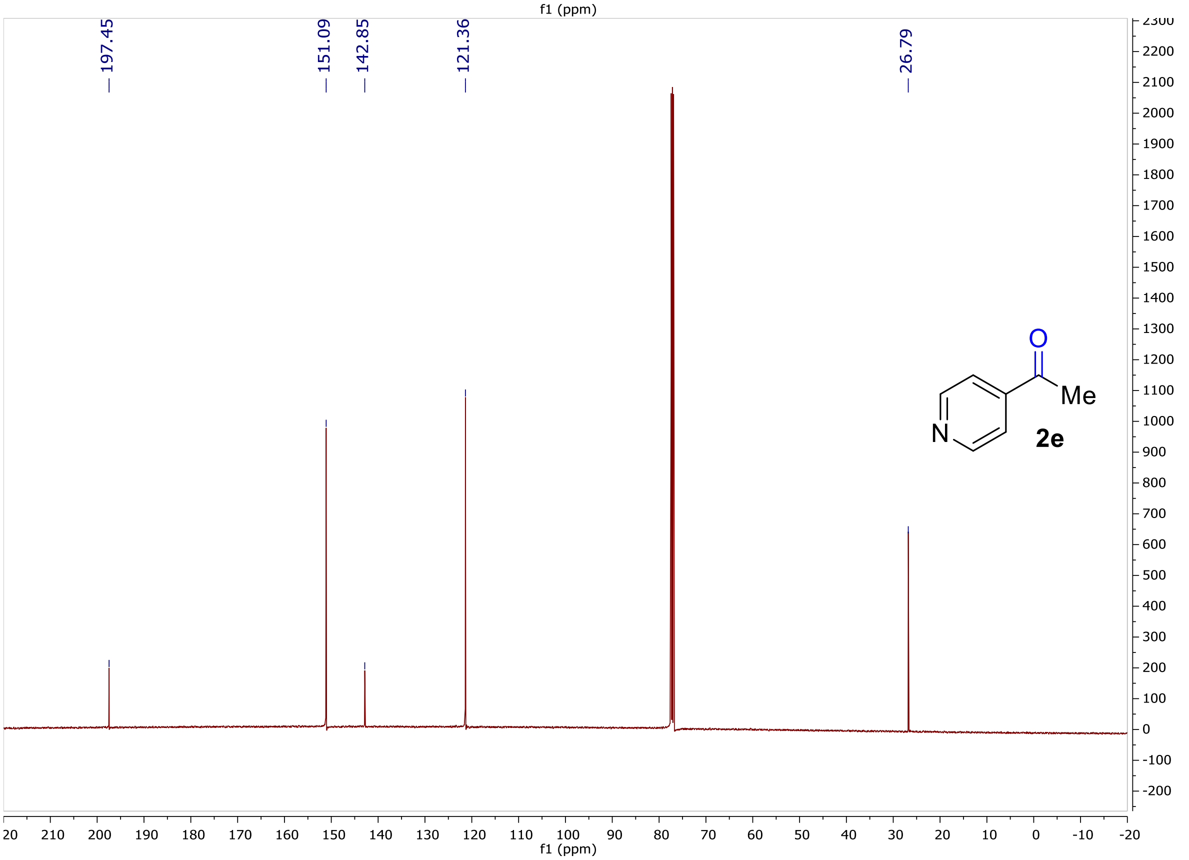
197.45
151.09
142.85
121.36
26.79
f1 (ppm)
O
Me
N
2e

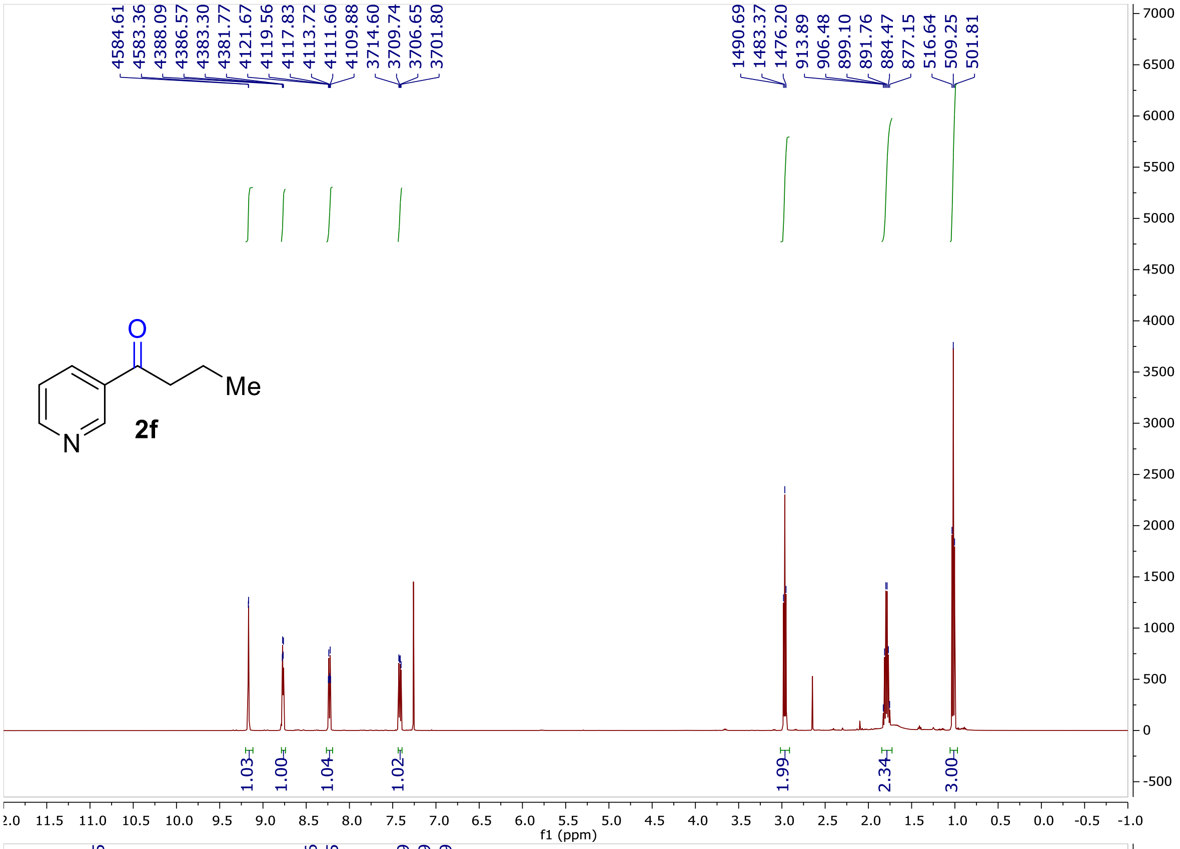
4584.61
4583.36
4388.09
4386.57
4383.30
4381.77
4121.67
4119.56
4117.83
4113.72
4111.60
4109.88
3714.60
3709.74
3706.65
3701.80
1490.69
1483.37
1476.20
913.89
906.48
899.10
891.76
884.47
877.15
516.64
509.25
501.81
Me
2f
N
O
1.03
1.00
1.04
1.02
1.99
2.34
3.00
f1 (ppm)

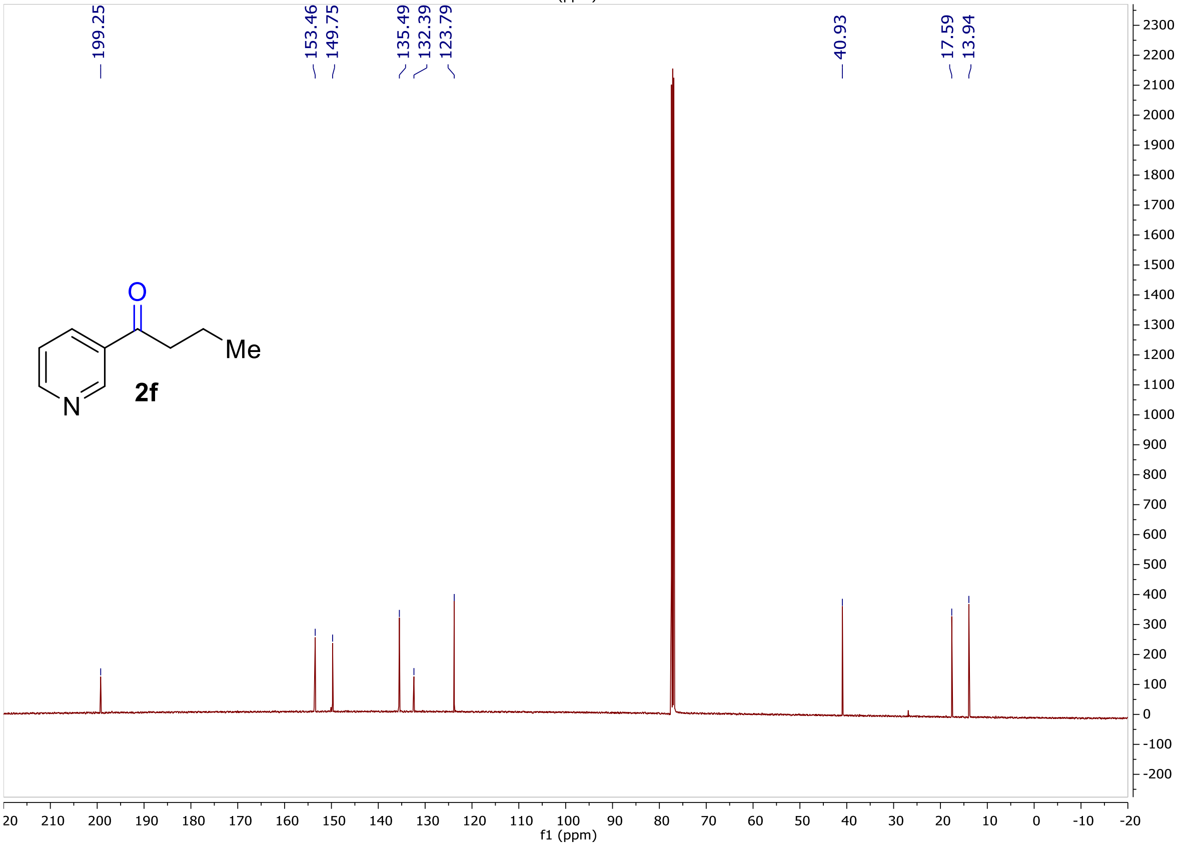
199.25
153.46
149.75
135.49
132.39
123.79
40.93
17.59
13.94
Me
2f
N
O
f1 (ppm)

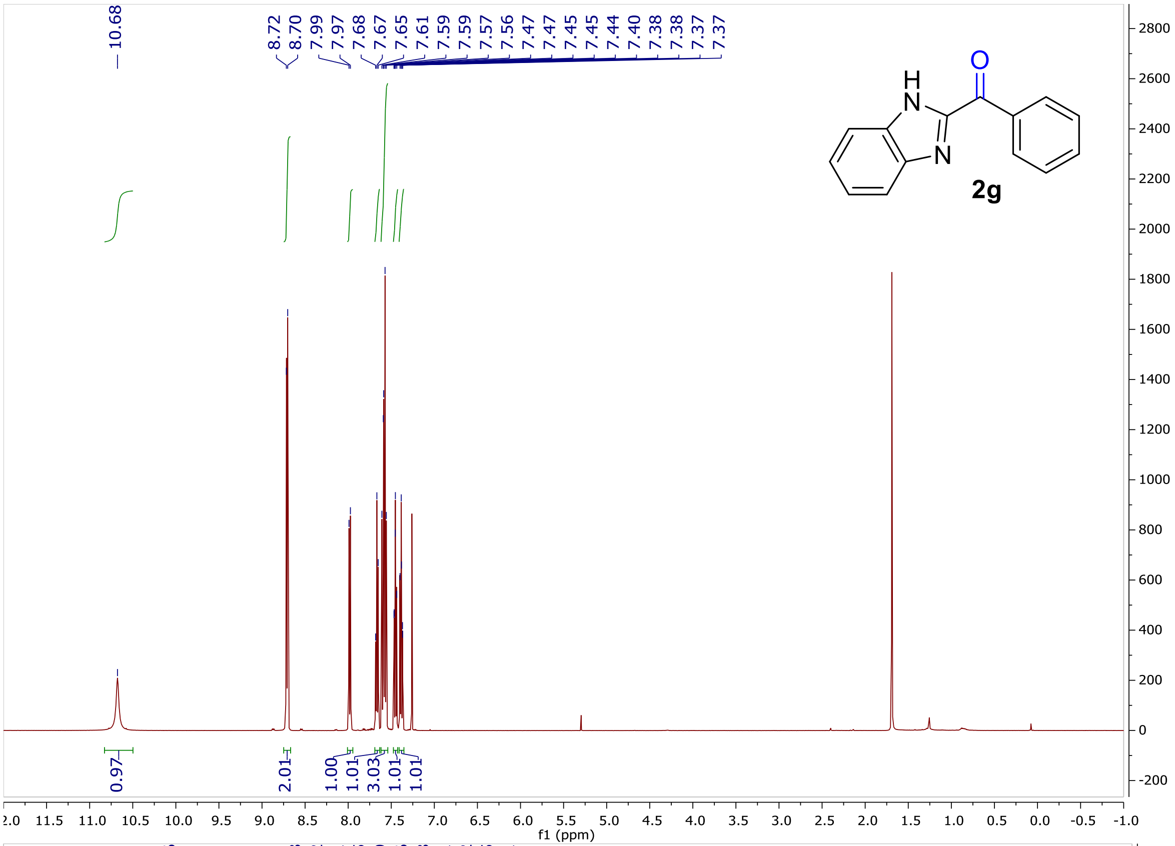
10.68
8.72
8.70
7.99
7.97
7.68
7.67
7.65
7.61
7.59
7.59
7.57
7.56
7.47
7.47
7.45
7.45
7.44
7.40
7.38
7.38
7.37
7.37
2g
0.97
2.01
1.00
1.01
3.03
1.01
1.01
f1 (ppm)

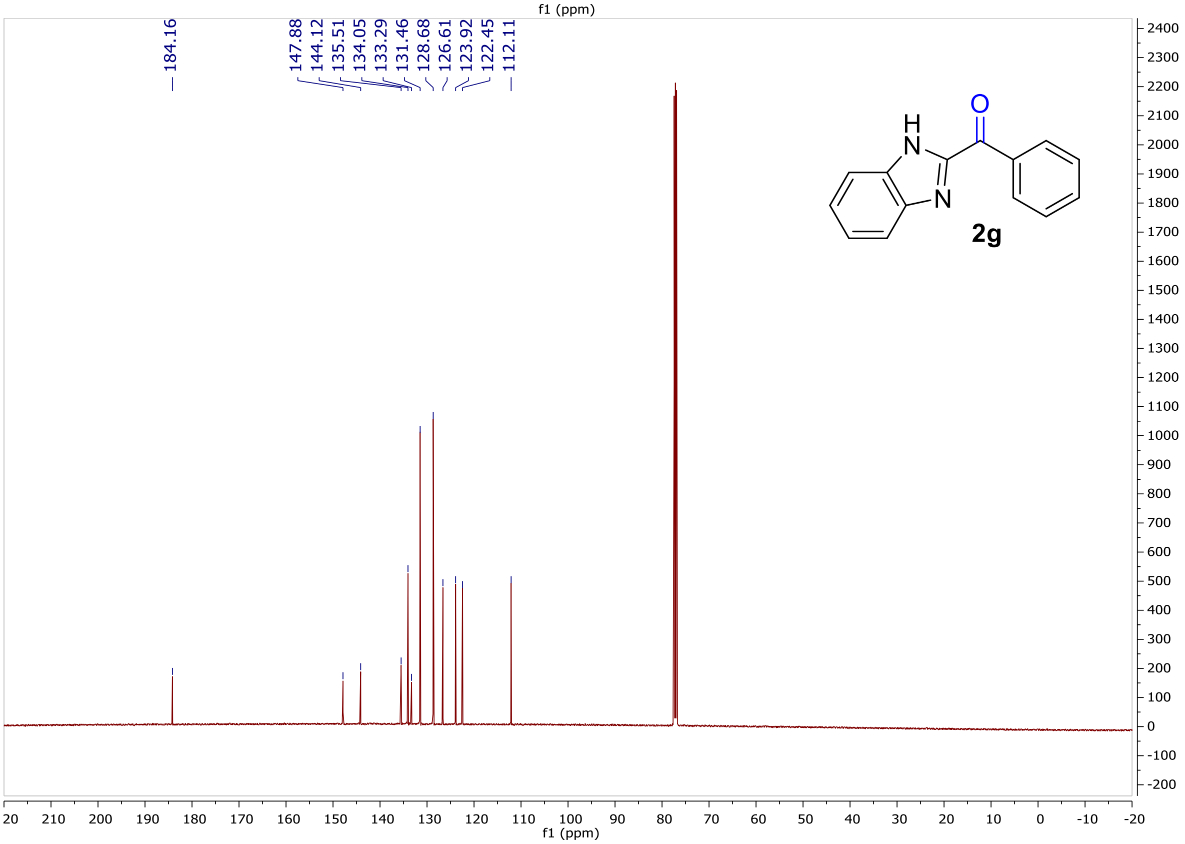
184.16
147.88
144.12
135.51
134.05
133.29
131.46
128.68
126.61
123.92
122.45
112.11
2g
f1 (ppm)

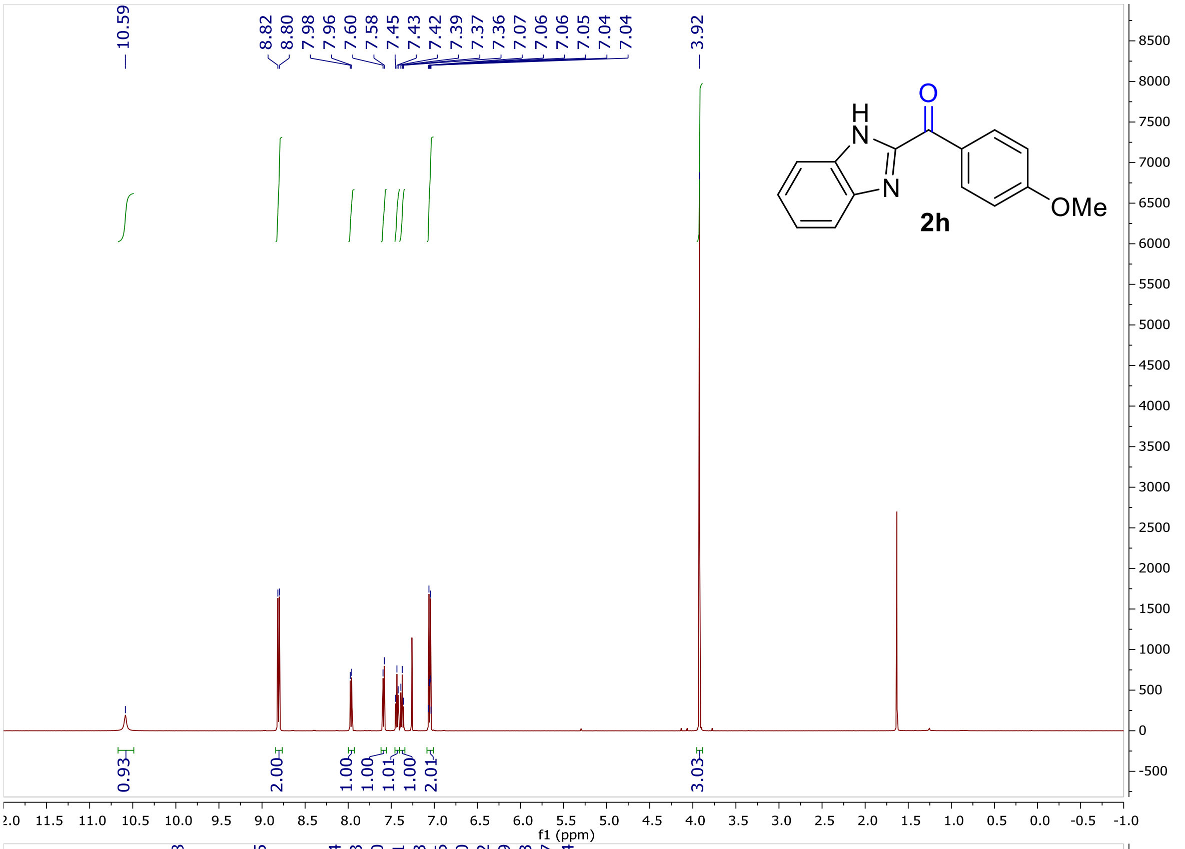
10.59
8.82
8.80
7.98
7.96
7.60
7.58
7.45
7.43
7.42
7.39
7.37
7.36
7.07
7.06
7.05
7.04
7.04
3.92
0.93
2.00
1.00
1.00
1.01
1.00
2.01
3.03
H
N
N
O
OMe
2h
f1 (ppm)

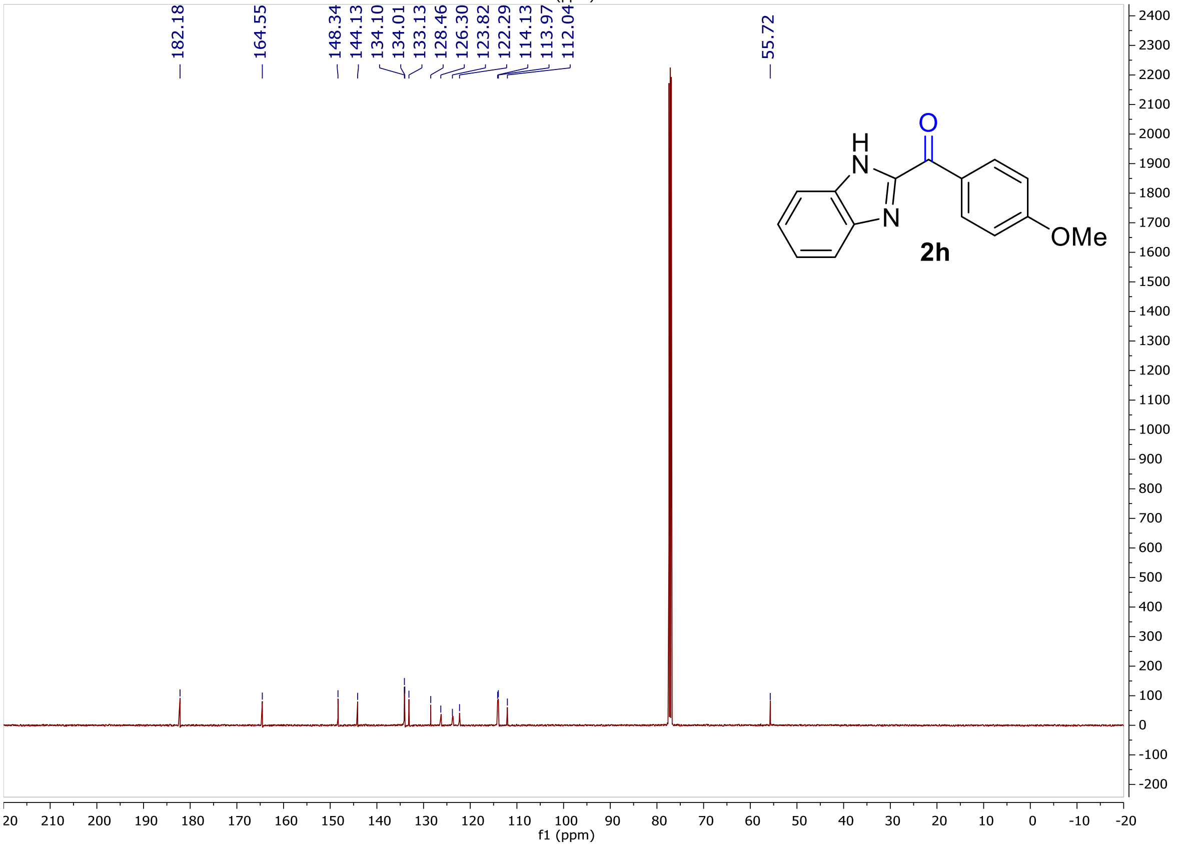
182.18
164.55
148.34
144.13
134.10
134.01
133.13
128.46
126.30
123.82
122.29
114.13
113.97
112.04
55.72
H
N
N
O
OMe
2h
f1 (ppm)

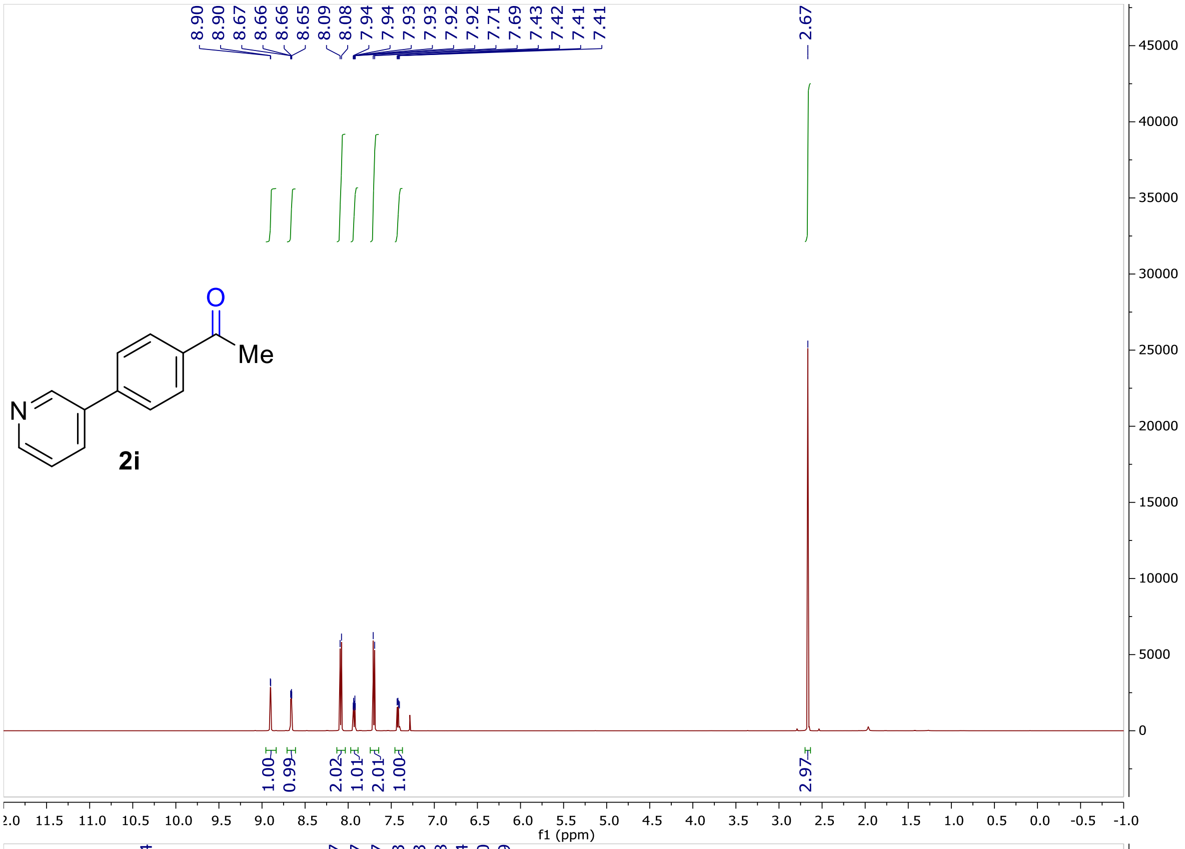
8.90
8.90
8.67
8.66
8.66
8.65
8.09
8.08
7.94
7.94
7.93
7.93
7.92
7.92
7.71
7.69
7.43
7.42
7.41
7.41
2.67
O
Me
N
2i
1.00
0.99
2.02
1.01
2.01
1.00
2.97
f1 (ppm)

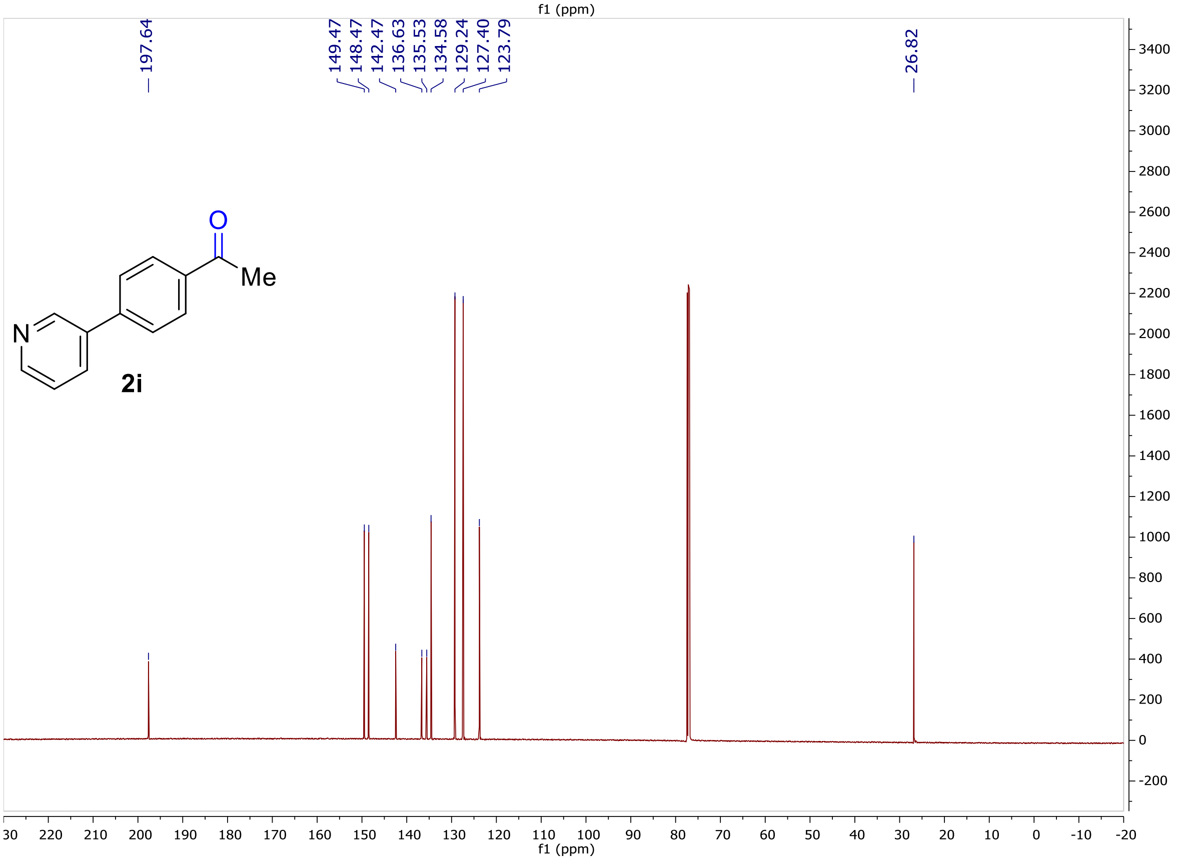
197.64
149.47
148.47
142.47
136.63
135.53
134.58
129.24
127.40
123.79
26.82
O
Me
N
2i
f1 (ppm)

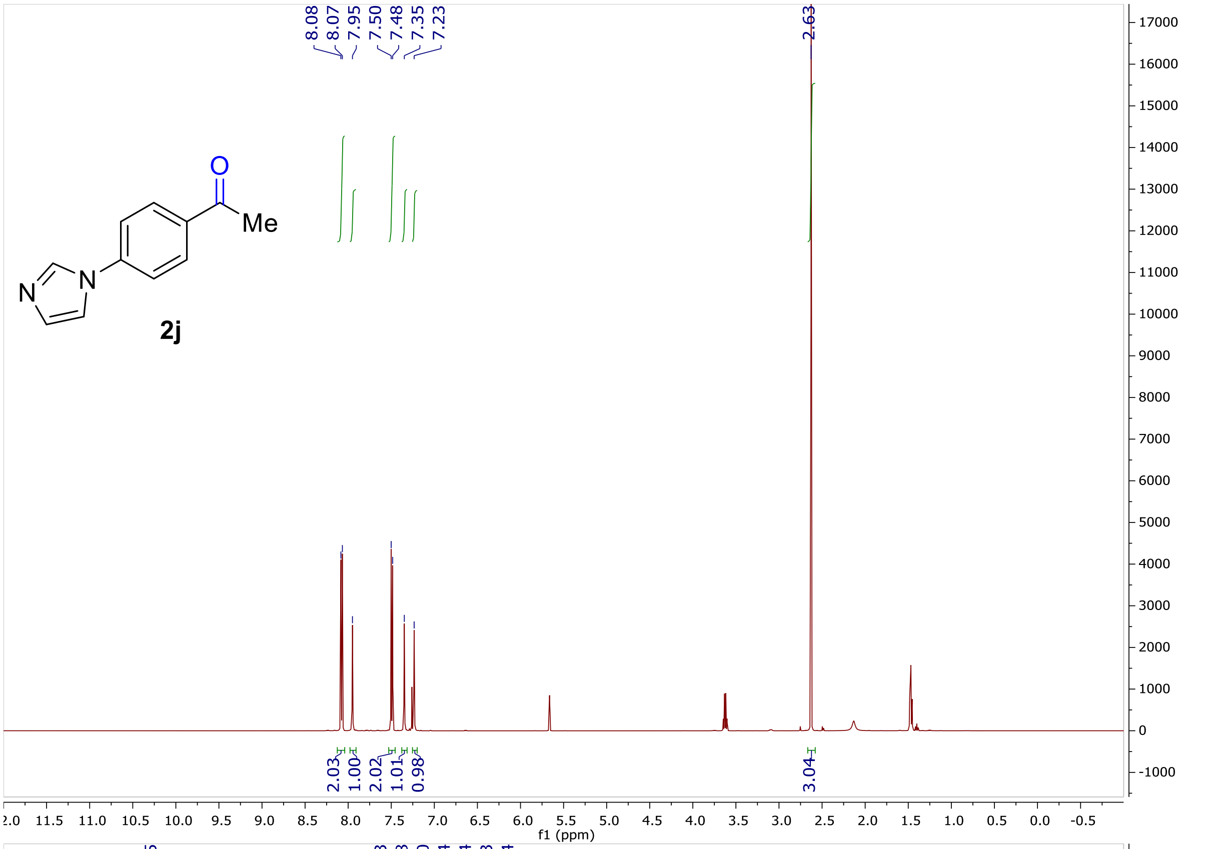
8.08
8.07
7.95
7.50
7.48
7.35
7.23
2.63
Me
2j
2.03
1.00
2.02
1.01
0.98
3.04
f1 (ppm)

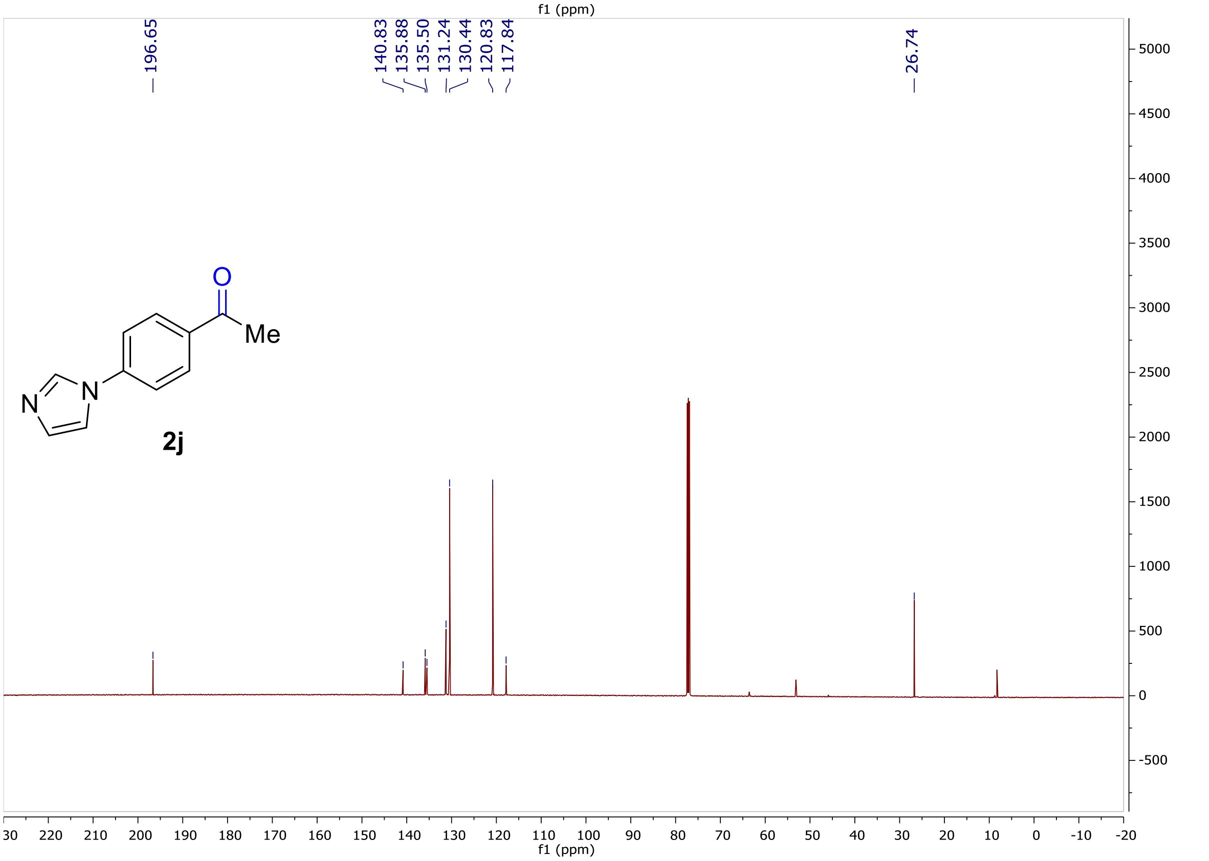
196.65
140.83
135.88
135.50
131.24
130.44
120.83
117.84
26.74
Me
2j
f1 (ppm)

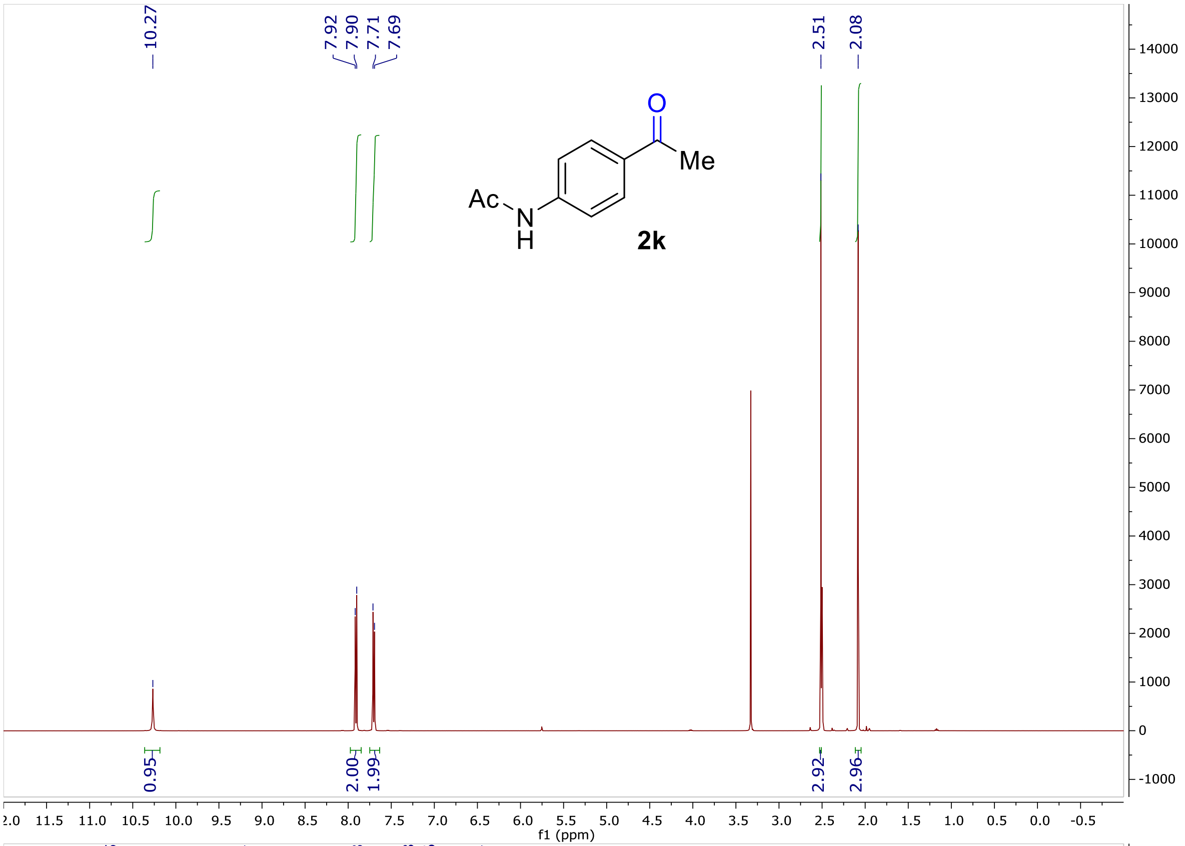

10.27
7.92
7.90
7.71
7.69
2.51
2.08
Ac
N
H
O
Me
2k
0.95
2.00
1.99
2.92
2.96
f1 (ppm)

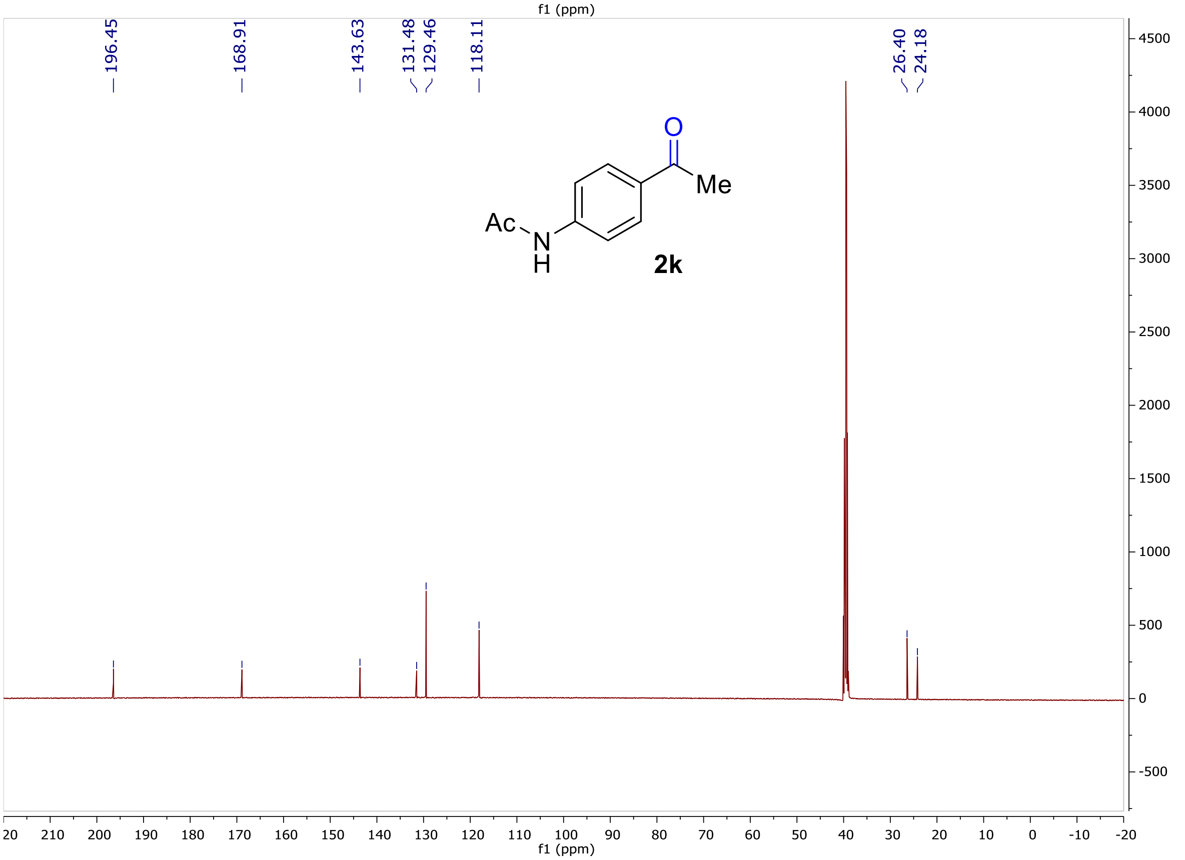

196.45
168.91
143.63
131.48
129.46
118.11
26.40
24.18
Ac
N
H
O
Me
2k
f1 (ppm)

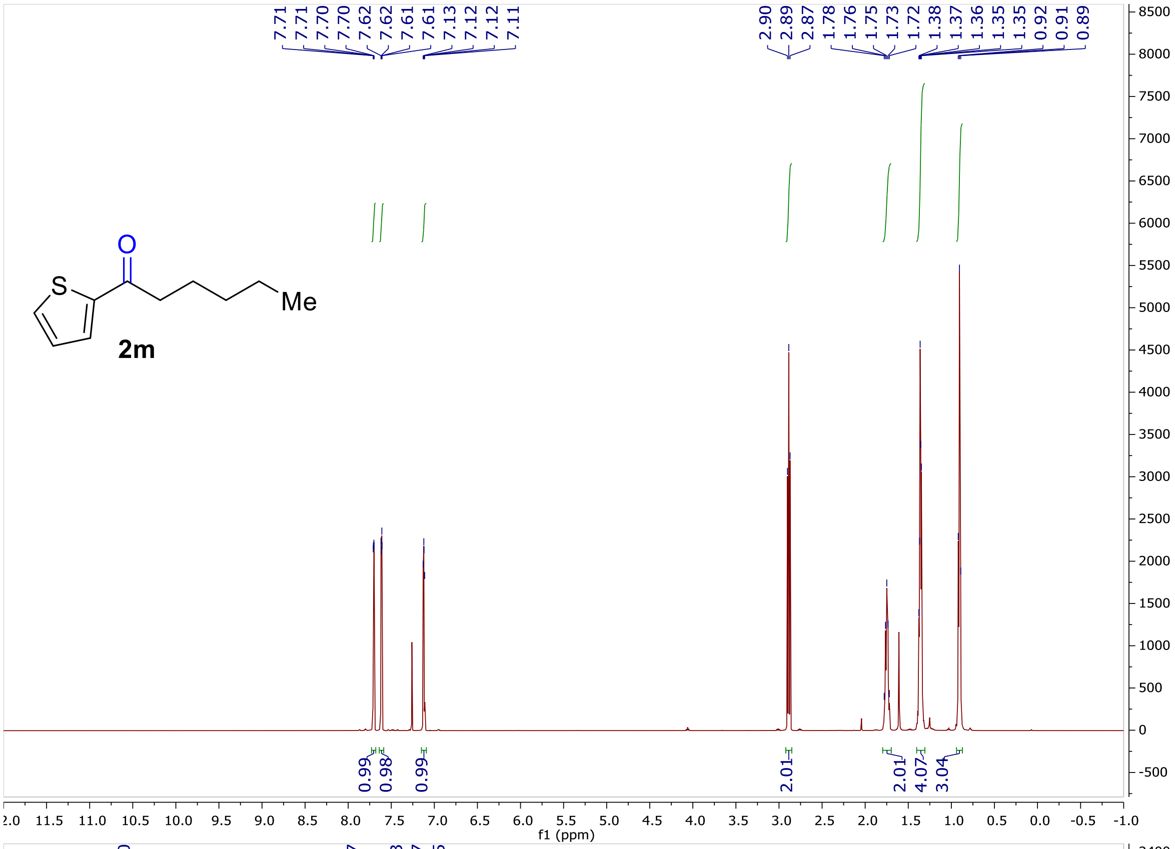
7.71
7.71
7.70
7.70
7.62
7.62
7.61
7.61
7.13
7.12
7.12
7.11
2.90
2.89
2.87
1.78
1.76
1.75
1.73
1.72
1.38
1.37
1.36
1.35
1.35
0.92
0.91
0.89
O
S
Me
2m
0.99
0.98
0.99
2.01
2.01
4.07
3.04
f1 (ppm)

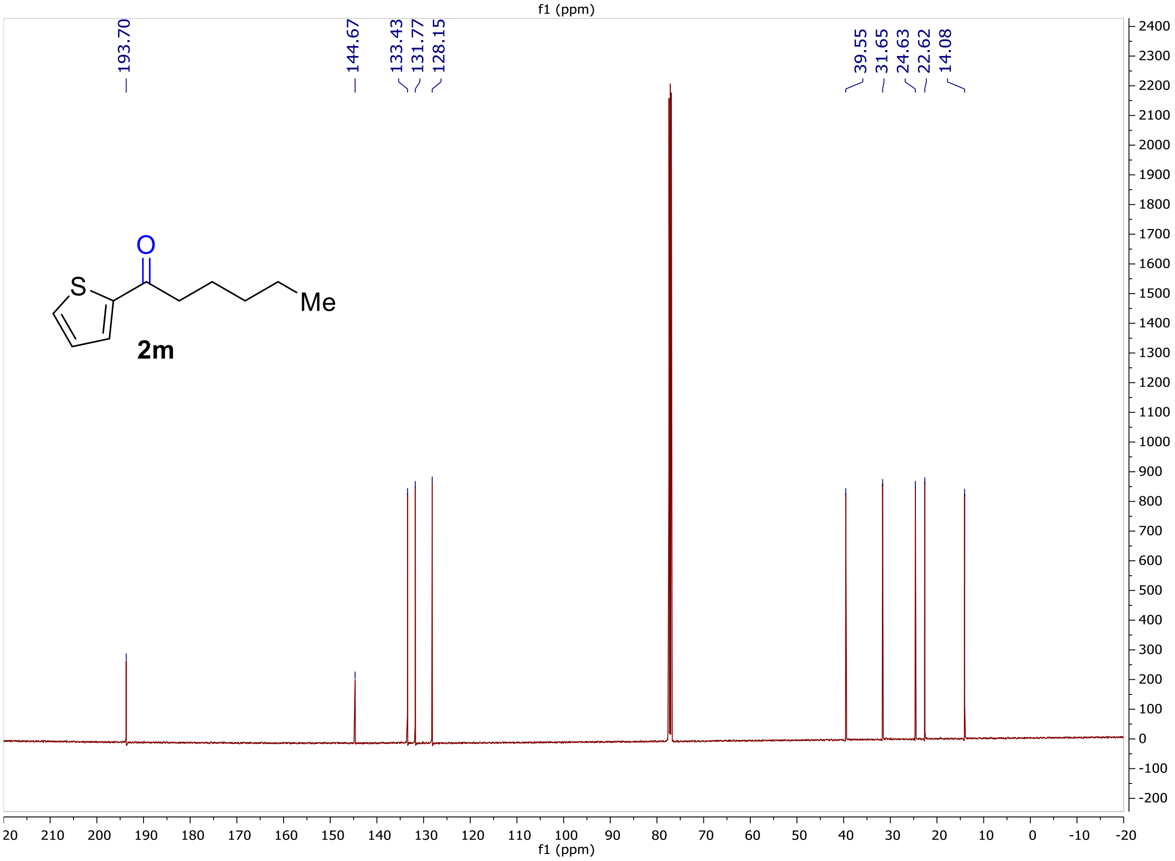
193.70
144.67
133.43
131.77
128.15
39.55
31.65
24.63
22.62
14.08
O
S
Me
2m
f1 (ppm)

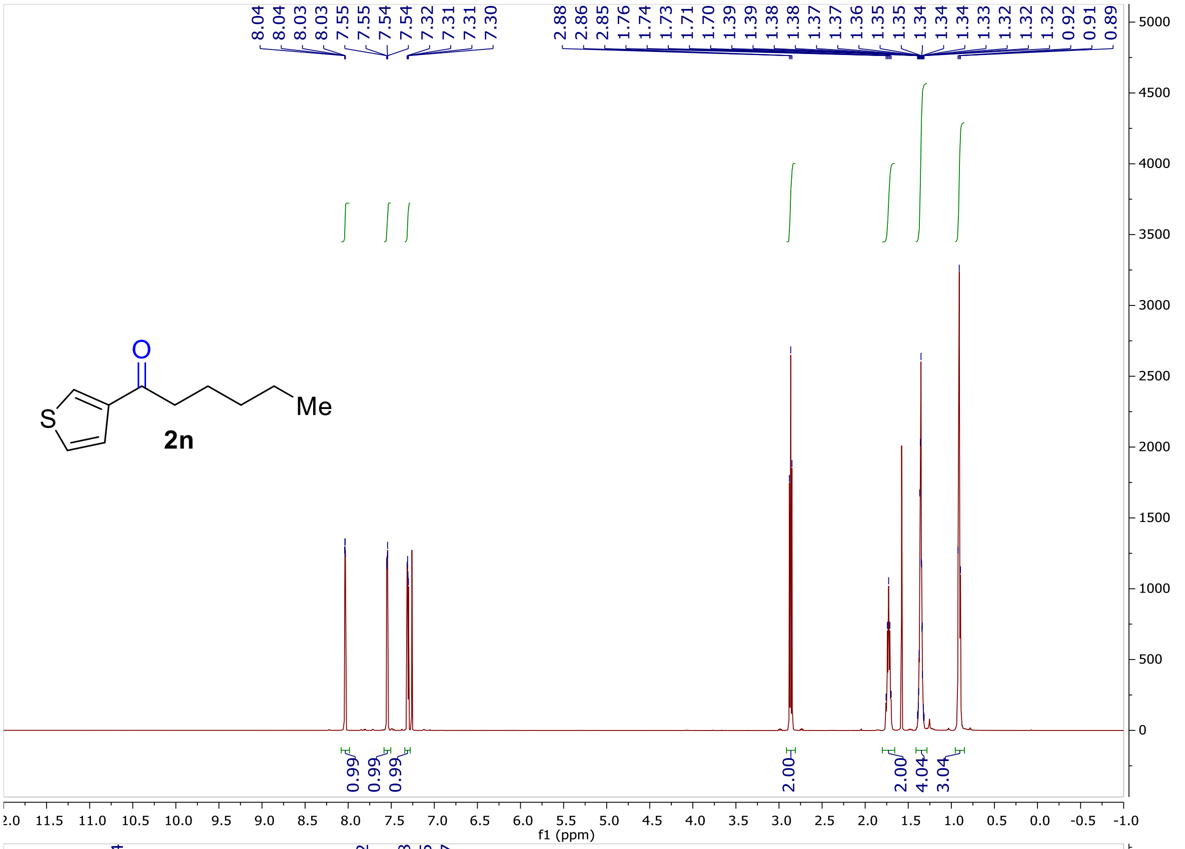

8.04
8.04
8.03
8.03
7.55
7.55
7.54
7.54
7.32
7.31
7.31
7.30
2.88
2.86
2.85
1.76
1.74
1.73
1.71
1.70
1.39
1.39
1.38
1.38
1.37
1.37
1.36
1.35
1.35
1.34
1.34
1.34
1.33
1.32
1.32
1.32
0.92
0.91
0.89
O
S
Me
2n
0.99
0.99
0.99
2.00
2.00
4.04
3.04
f1 (ppm)

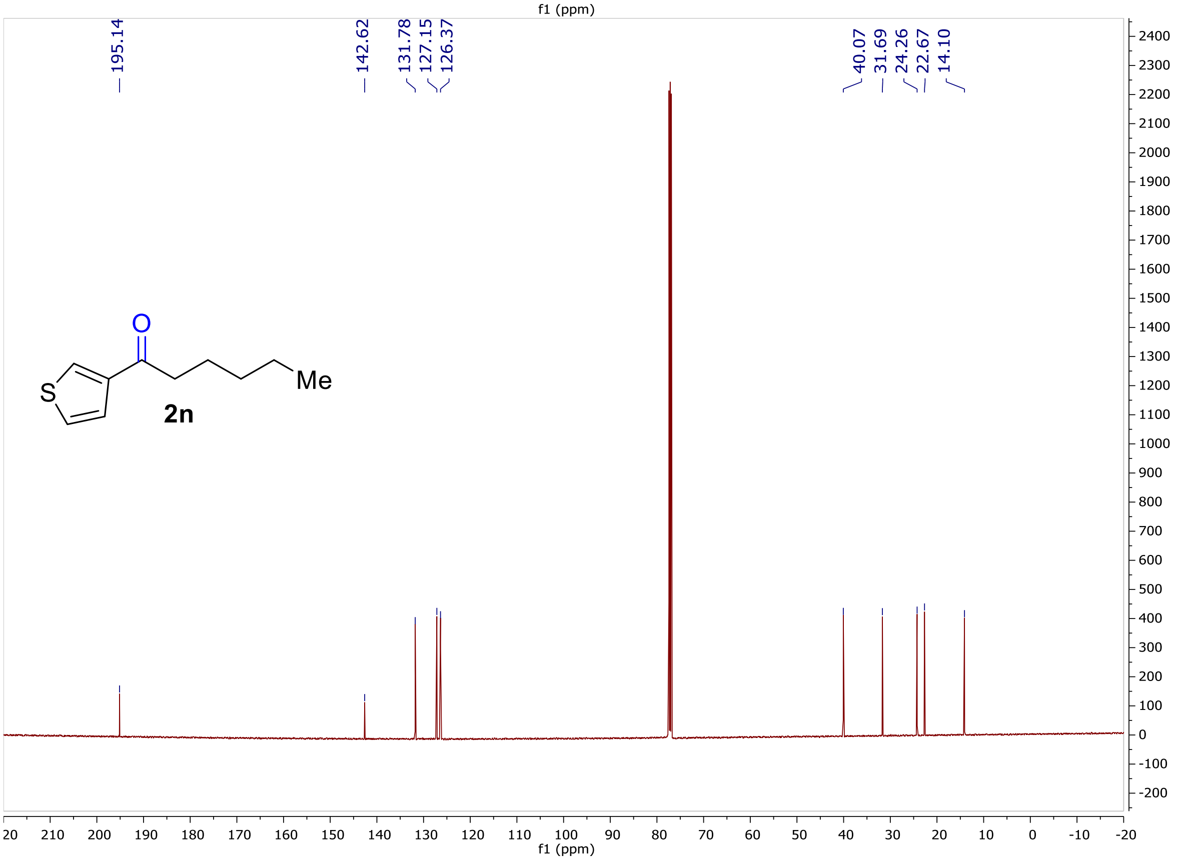

195.14
142.62
131.78
127.15
126.37
40.07
31.69
24.26
22.67
14.10
O
S
Me
2n
f1 (ppm)

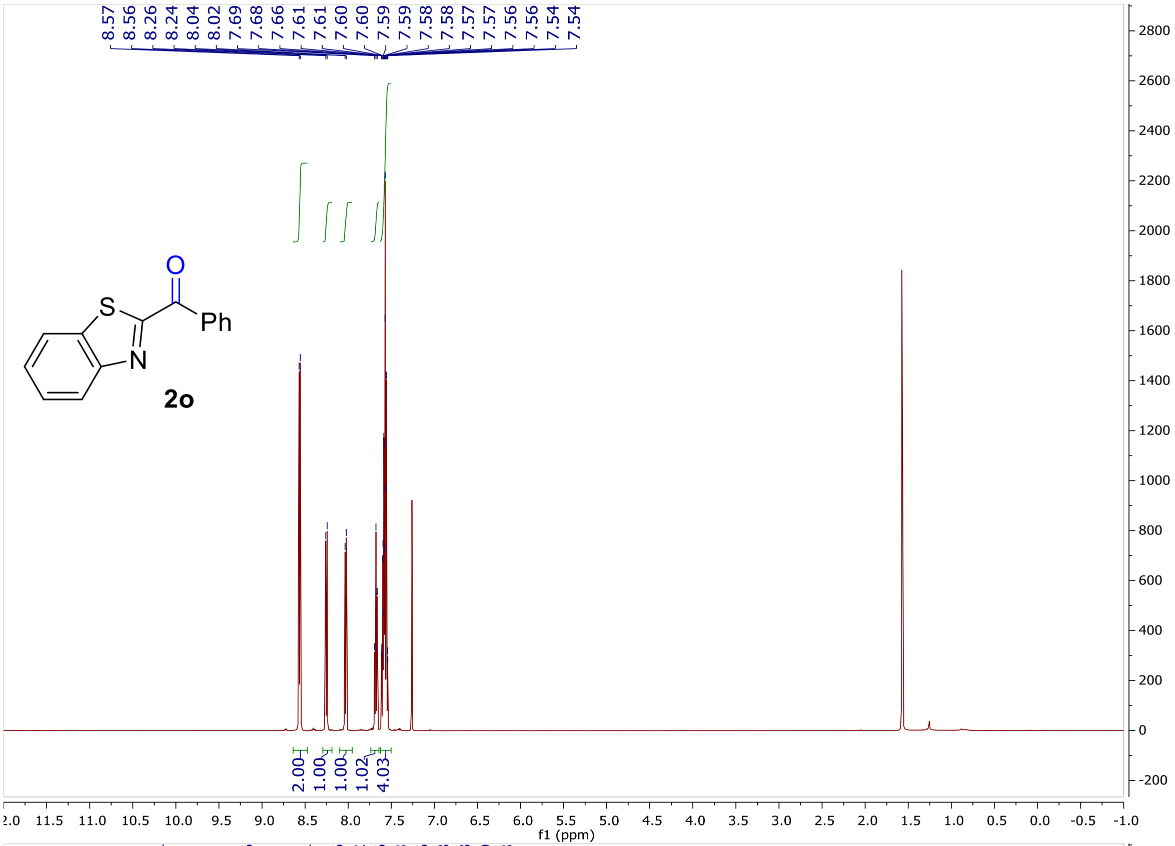
8.57 8.56 8.26 8.24 8.04 8.02 7.69 7.68 7.66 7.61 7.61 7.60 7.60 7.59 7.59 7.58 7.58 7.57 7.57 7.56 7.54 7.54
2.00 1.00 1.00 1.02 4.03
Ph
2o
f1 (ppm)

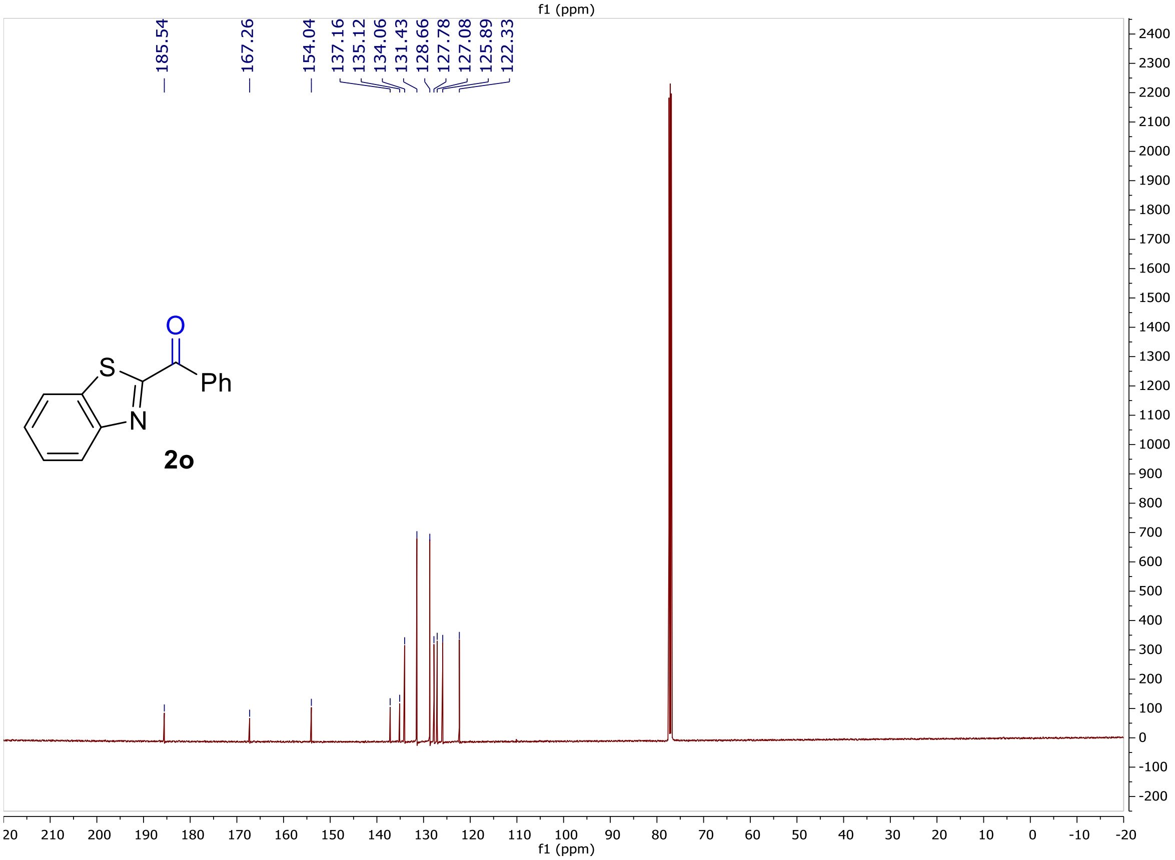
185.54 167.26 154.04 137.16 135.12 134.06 131.43 128.66 127.78 127.08 125.89 122.33
Ph
2o
f1 (ppm)

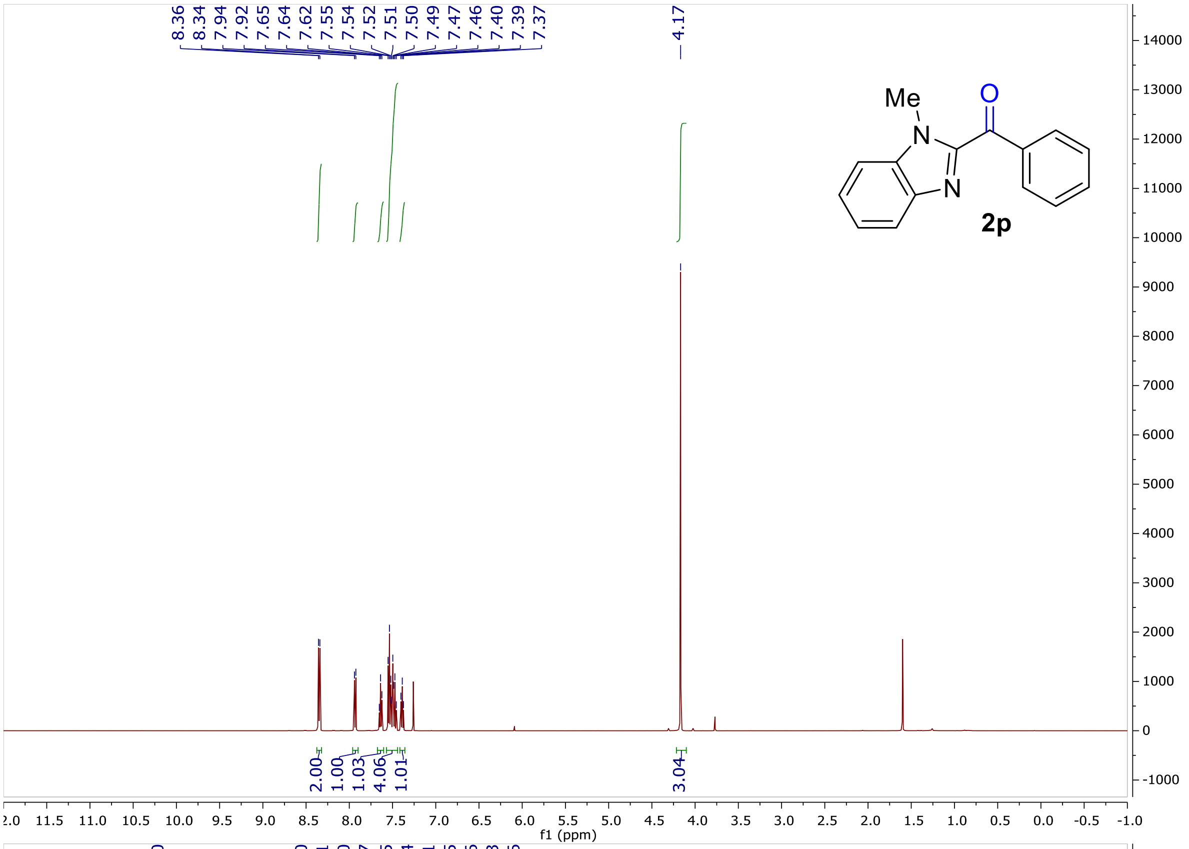
8.36
8.34
7.94
7.92
7.65
7.64
7.62
7.55
7.54
7.52
7.51
7.50
7.49
7.47
7.46
7.40
7.39
7.37
4.17
Me
N
O
N
2p
2.00
1.00
1.03
4.06
1.01
3.04
f1 (ppm)

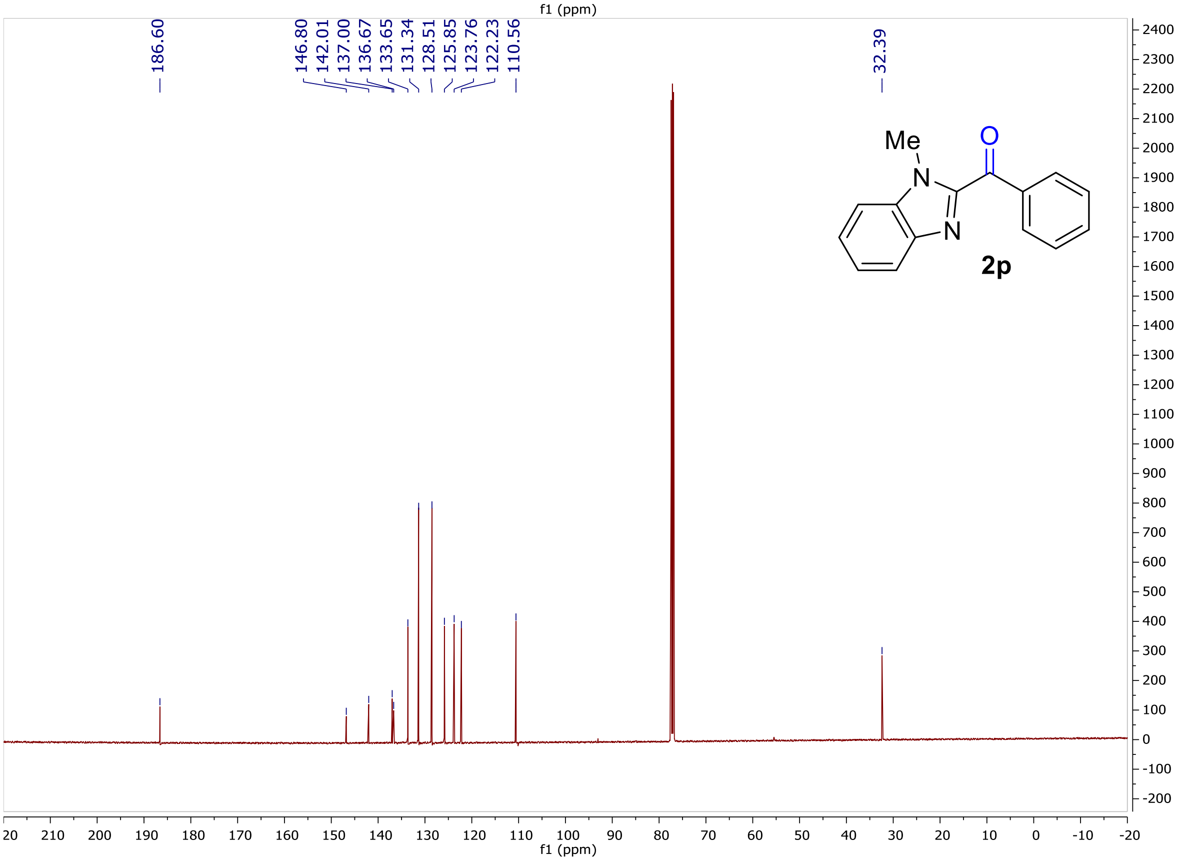
186.60
146.80
142.01
137.00
136.67
133.65
131.34
128.51
125.85
123.76
122.23
110.56
32.39
Me
N
O
N
2p
f1 (ppm)

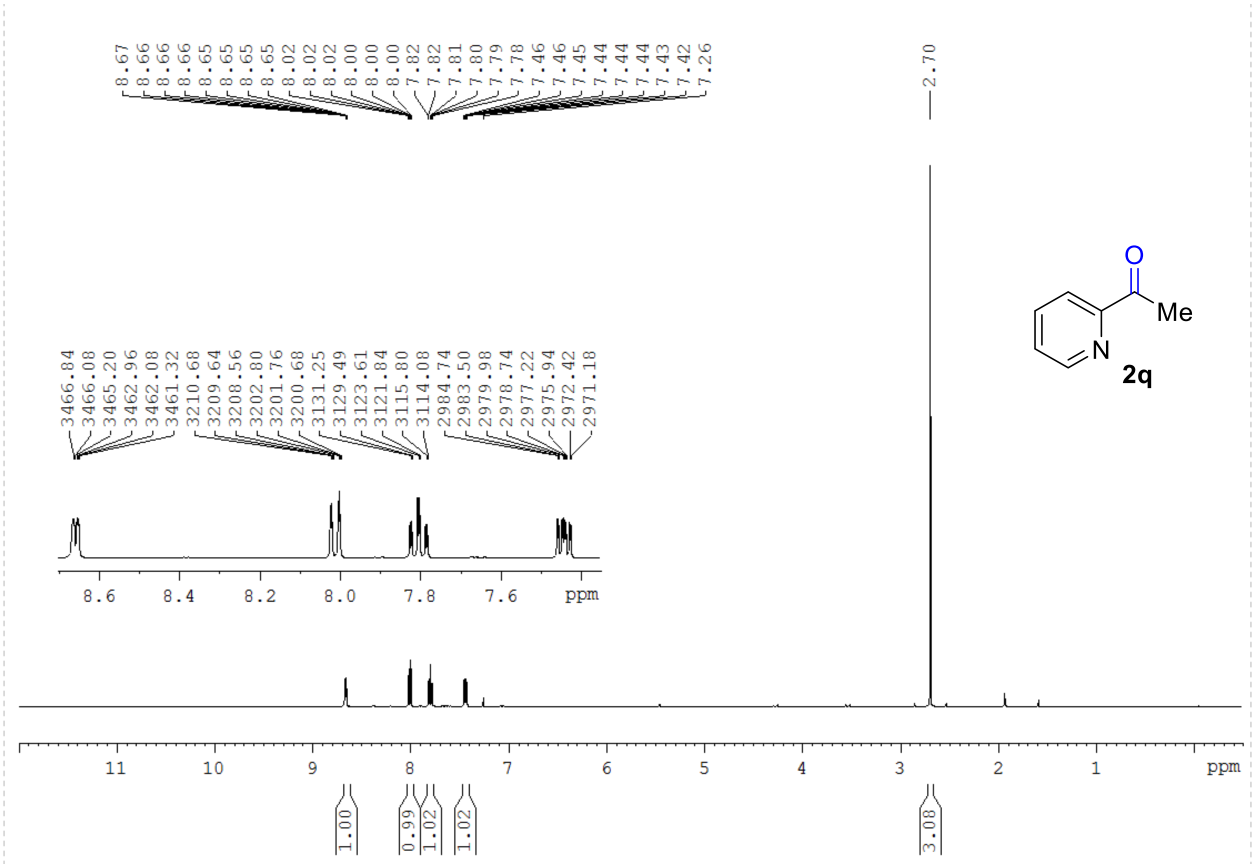

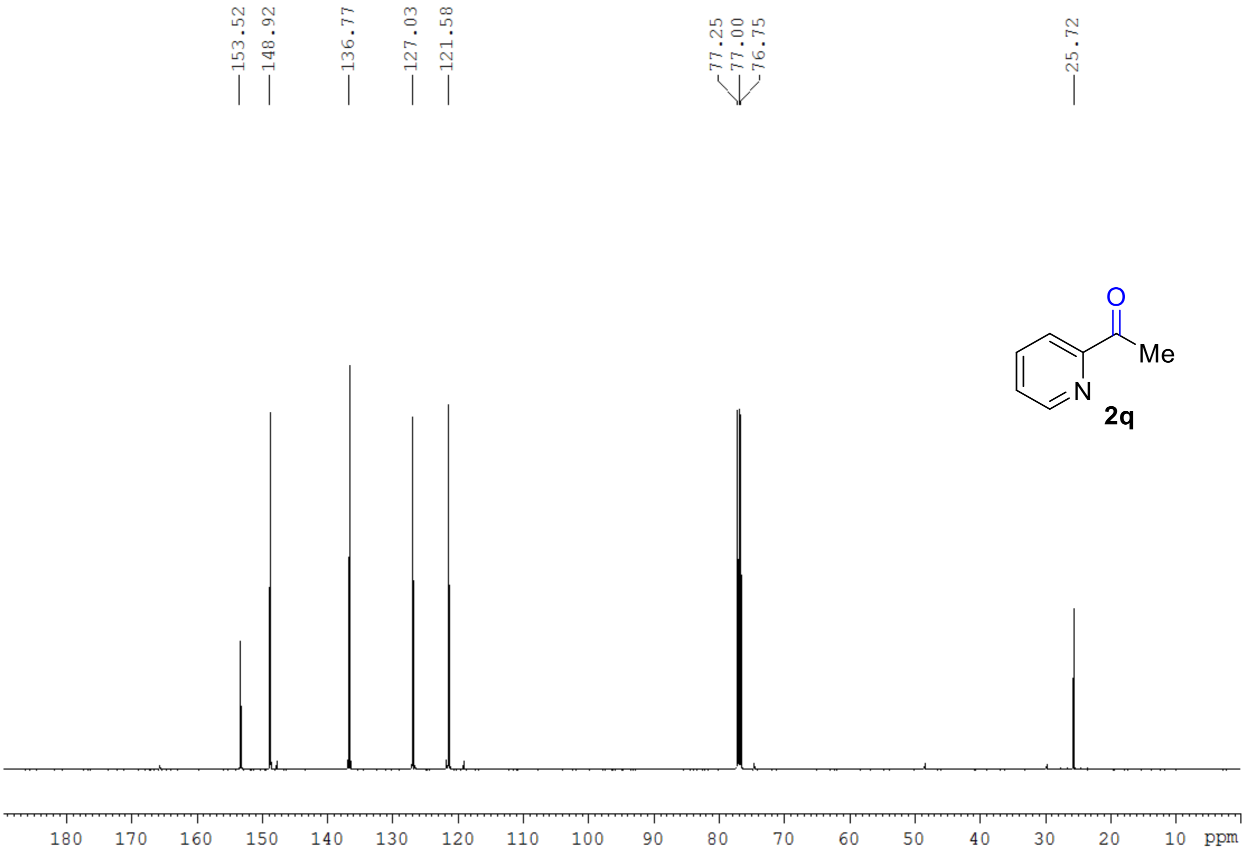

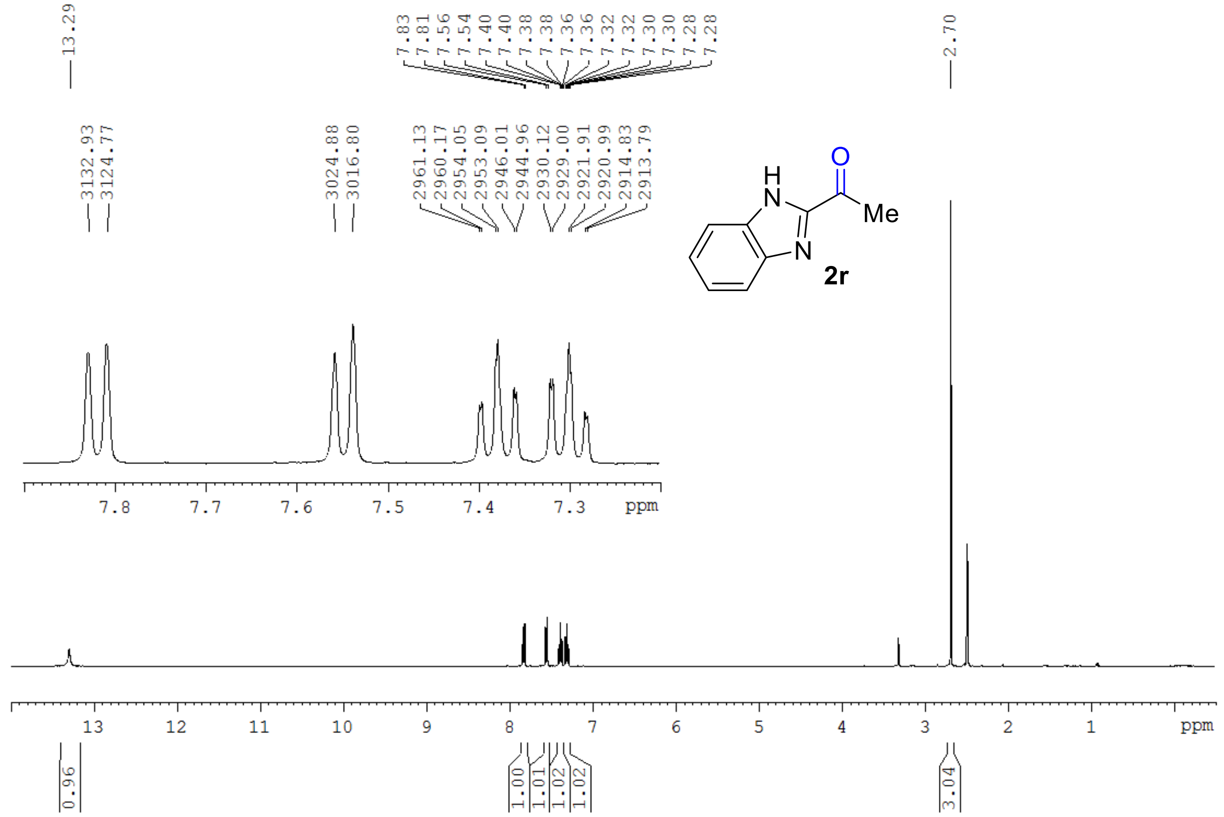
13.29
7.83
7.81
7.56
7.54
7.40
7.40
7.38
7.38
7.36
7.36
7.32
7.32
7.30
7.30
7.28
7.28
2.70
3132.93
3124.77
3024.88
3016.80
2961.13
2960.17
2954.05
2953.09
2946.01
2944.96
2930.12
2929.00
2921.91
2920.99
2914.83
2913.79
H
N
O
Me
N
2r
7.8
7.7
7.6
7.5
7.4
7.3
ppm
13
12
11
10
9
8
7
6
5
4
3
2
1
ppm
0.96
1.00
1.01
1.02
1.02
3.04

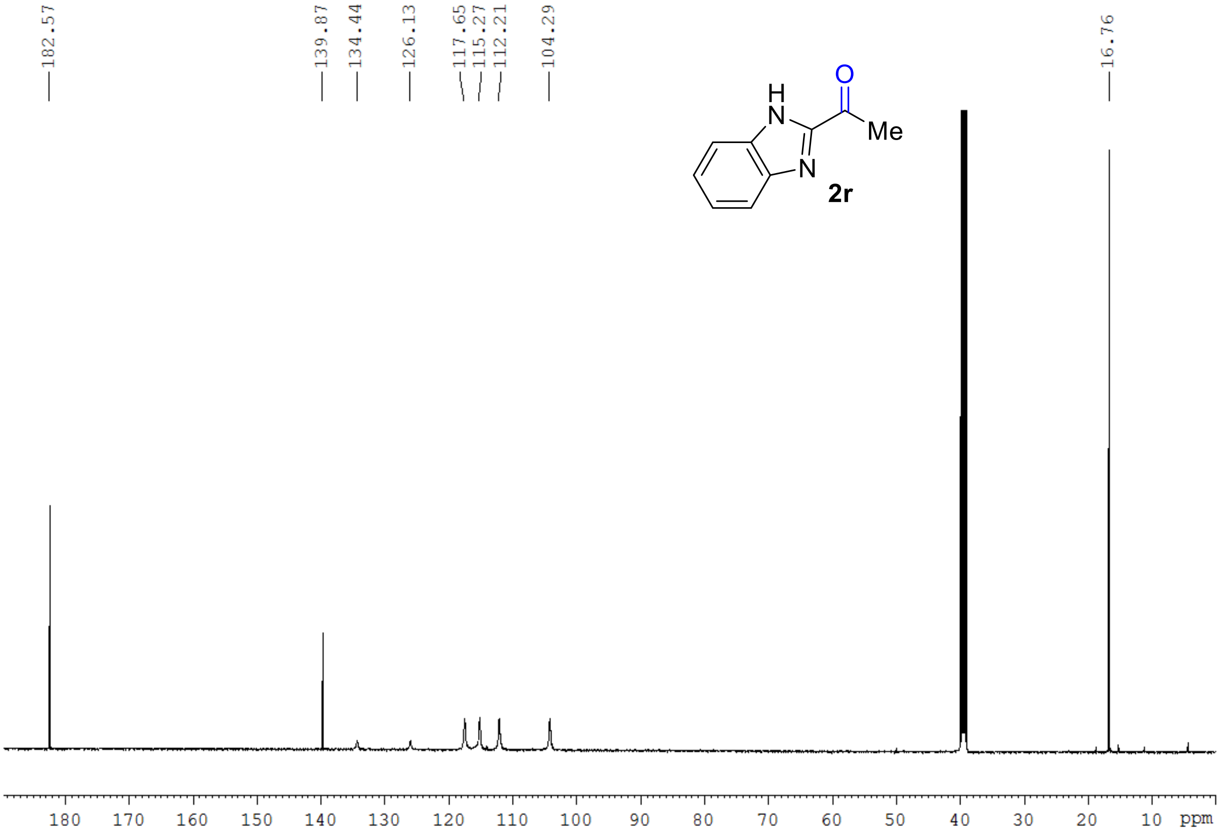
182.57
139.87
134.44
126.13
117.65
115.27
112.21
104.29
16.76
H
N
O
Me
N
2r
180
170
160
150
140
130
120
110
100
90
80
70
60
50
40
30
20
10
ppm

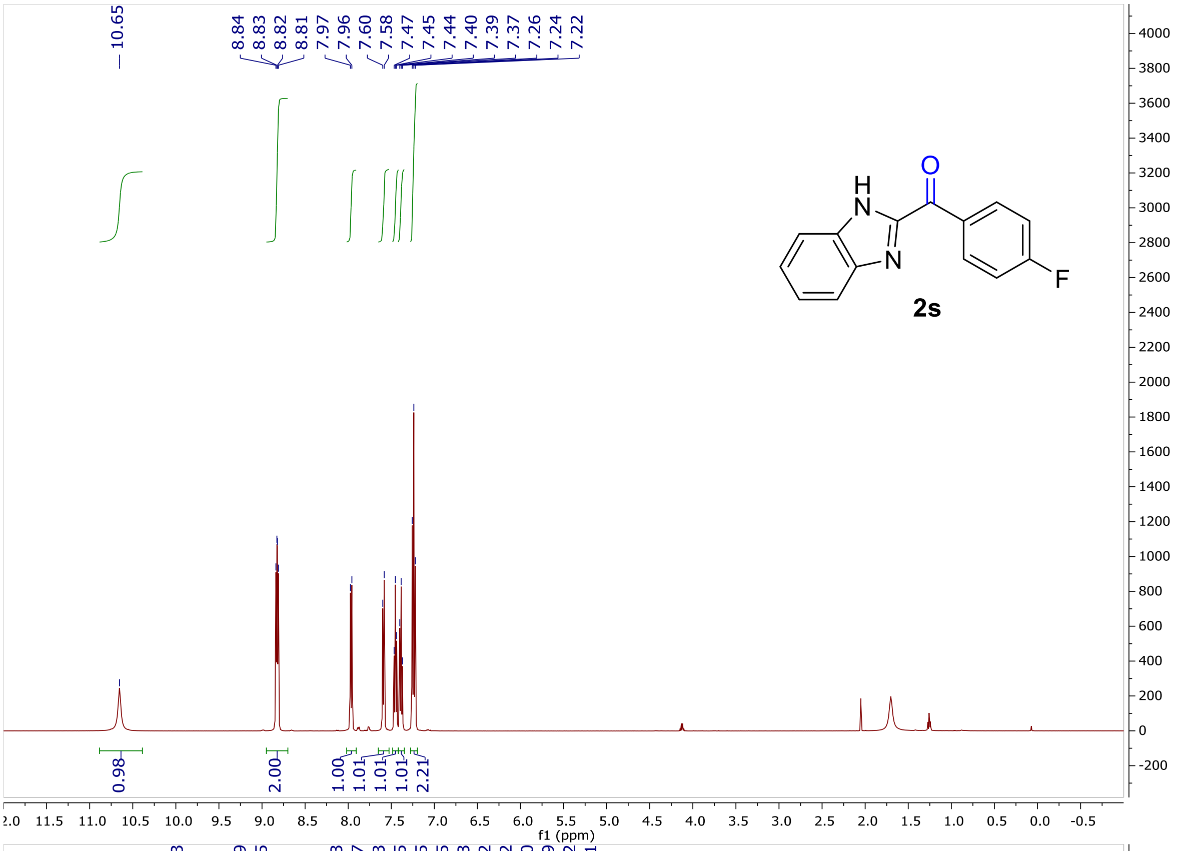
10.65
8.84
8.83
8.82
8.81
7.97
7.96
7.60
7.58
7.47
7.45
7.44
7.40
7.39
7.37
7.26
7.24
7.22
0.98
2.00
1.00
1.01
1.01
1.01
2.21
2s
f1 (ppm)

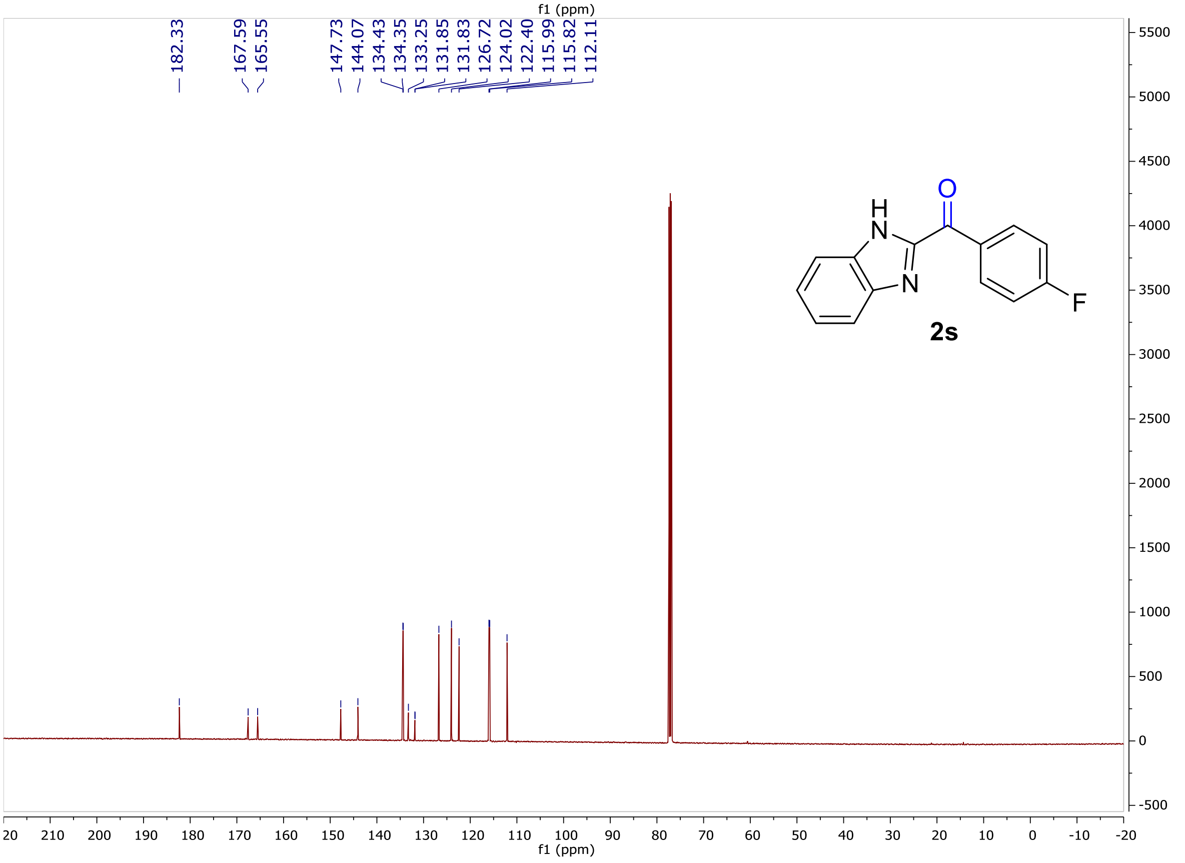
182.33
167.59
165.55
147.73
144.07
134.43
134.35
133.25
131.85
131.83
126.72
124.02
122.40
115.99
115.82
112.11
2s
f1 (ppm)

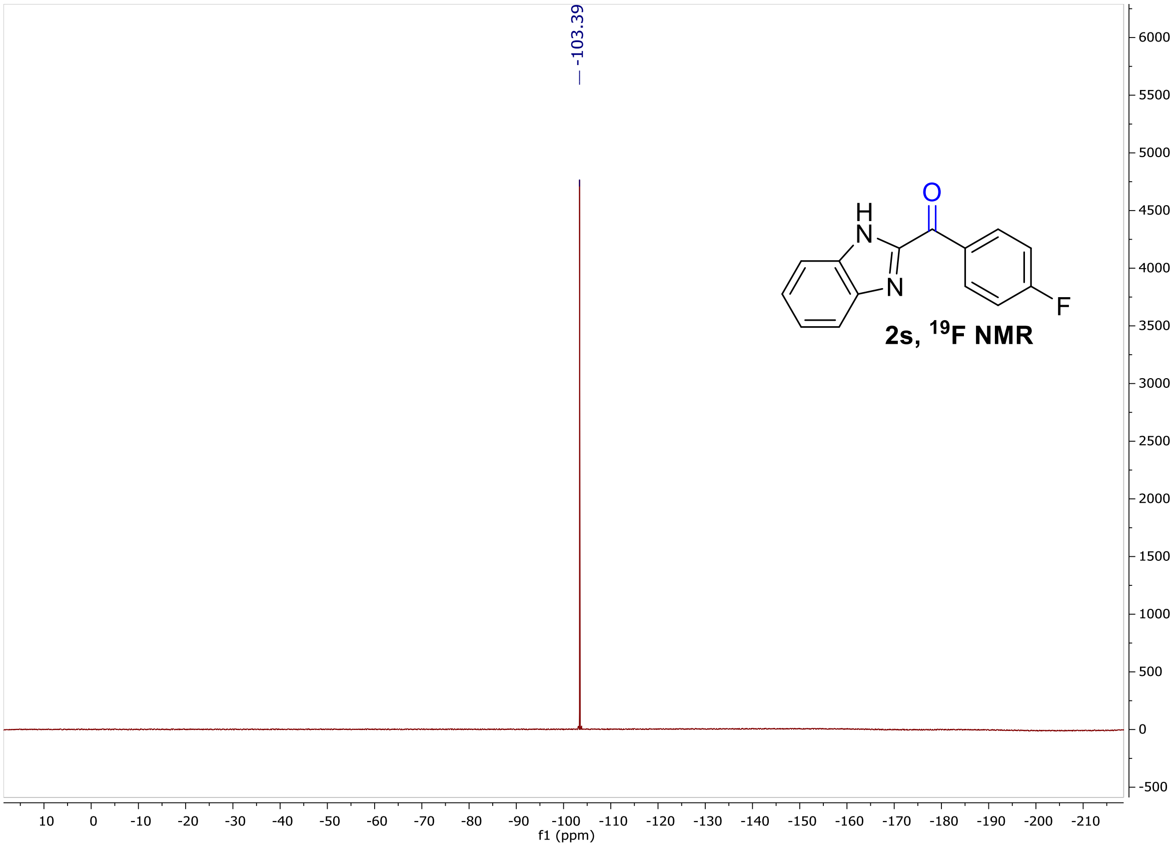
-103.39
2s, ¹⁹F NMR
f1 (ppm)
